# Human Exposures to Bisphenol A, Bisphenol F and Chlorinated Bisphenol A Derivatives and Thyroid Function

**DOI:** 10.1371/journal.pone.0155237

**Published:** 2016-10-26

**Authors:** Xanthi D. Andrianou, Stephanie Gängler, Andra Piciu, Pantelis Charisiadis, Christina Zira, Kyriacos Aristidou, Doina Piciu, Russ Hauser, Konstantinos C. Makris

**Affiliations:** 1 Cyprus International Institute for Environmental and Public Health, Cyprus University of Technology, Limassol, Cyprus; 2 Endocrinology Department, Archbishop Makarios III Hospital, Nicosia, Cyprus; 3 Radiology Department, Archbishop Makarios III Hospital, Nicosia, Cyprus; 4 Medical Oncology Department, Prof. Dr. Ion Chiricuta Institute of Oncology, Cluj-Napoca, Romania; 5 Iuliu Hatieganu University of Medicine and Pharmacy, Cluj-Napoca, Romania; 6 Nuclear Medicine Department, Prof. Dr. Ion Chiricuta Institute of Oncology, Cluj-Napoca, Romania; 7 Department of Environmental Health, Harvard T.H. Chan School of Public Health, Harvard University, Boston, United States of America; Universidade Federal do Rio de Janeiro, BRAZIL

## Abstract

Although the increasing prevalence of thyroid nodular disease (TND) has been partially attributed to the more frequent usage of improved diagnostics, environmental factors, such as exposures to thyroid-disrupting chemicals may contribute to TND and altered thyroid function. We investigated the association between exposures to bisphenol A (BPA), its chlorinated derivatives (ClxBPA), and bisphenol F (BPF) with TND and thyroid measures in adult women. A case-control study in Cyprus and Romania (*n* = 212) was conducted, where cases were those with thyroid nodules (diameter >3mm), and controls without nodules. Serum TSH and free thyroxine and urinary levels of BPA, BPF and ClxBPA were measured using immunoassays and tandem mass spectrometry, respectively. The association between exposures to BPA compounds and TND, adjusting for age, BMI, thyroid hormones and urinary iodine was assessed using logistic regression. Linear regression was used to explore associations between urinary BPA, BPF and ClxBPA and serum thyroid hormones. With the exception of a chlorinated BPA compound (30%), the rest of bisphenols were quantified in 100% of urine samples. A positive and significant (p<0.05) association was observed between urinary BPA and serum TSH that remained after adjusting for urinary creatinine, age, BMI, study site and disease status; there was no significant association between BPF or ClxBPA with TSH. None of the BPA compounds were associated with higher odds of TND. Our study found associations of urinary BPA with TSH but not with BPF or ClxBPA. A larger study would be justified.

## Introduction

Thyroid nodules are discrete lesions in the thyroid gland that are either benign or malignant [1]. The increasing prevalence of thyroid nodular disease (TND) is paralleled by the increasing prevalence of thyroid cancer and more frequent usage of improved diagnostic methods, such as ultrasonography [2]. Although in most cases only a small proportion (~5%) of the thyroid nodules progresses to malignancy, the periodic monitoring of thyroid nodules is often advised, posing a burden to both individuals and healthcare systems [3].

Genetic and environmental factors including iodine deficiency have been linked with the development of TND [4–6]. Endocrine disrupting chemicals (EDCs) have been studied in terms of their effect on thyroid homeostasis as they may affect the thyroid gland in various ways [7]. The maintenance of the thyroid gland’s homeostasis requires the ‘synchronized cooperation’ of multiple systems in the thyroid gland itself, the peripheral metabolism and the binding proteins; thus, disruption or interference at any point may cause adverse effects on the organism [8]. The thyroid disrupting properties of EDCs, such as bisphenol A (BPA), pesticides or even ultraviolet filters have been demonstrated in both human and animal studies [7,9,10]. However, BPA is has been mostly linked with disruption of estrogenic activity [7].

BPA is a synthetic monomer used in polycarbonate plastics and epoxy resins. It is produced globally in high volumes and used in a wide variety of products such as plastic or metallic food containers or other food-contact items including bottles [11]. BPA has been linked with adverse health effects and therefore it has been banned from various products such as baby bottles [12,13]. Bisphenol F (BPF) is a structural analog of BPA that is used in epoxy resins and other plastic products usually substituting BPA in their polymeric structure [14,15]. BPF has been detected in personal care products (PCPs), indoor dust, as well as in human urine [16–18]. The reactivity of BPA with disinfectant chlorine or chlorinated oxidants is evident in the instantaneous formation of chlorinated BPA derivatives (ClxBPA) in various environmental media that show increased estrogen-activity when compared with that of BPA [19].

Possible links between thyroid function and exposures to BPA have not been fully studied in humans. One study in school-aged children recently showed an inverse relationship between urinary BPA levels and thyroid volume and a negative association of the risk for thyroid multinodularity and BPA [20]. Perturbations in thyroid hormones, i.e. thyroid stimulating hormone (TSH) or thyroxine (T4) as a result of exposures to BPA have been documented for the general population, pregnant women or in occupational settings, but often generating inconsistent results [21–24].

The epidemiology of thyroid disorders and TND has not been recently studied either in Cyprus or Romania; these countries share contrasting characteristics with regards to thyroid cancer comparative prevalence estimates. In Cyprus, thyroid cancer is the 3^rd^ most prevalent type of cancer among females, but in Romania, it ranks 12^th^. Overall, Cyprus is 6^th^ and Romania 28^th^ in the list of countries with the highest thyroid cancer comparative prevalence rates among 41 countries of the European region [25–27]. A national iodine fortification program is implemented in Romania where table salt and salt used in bakery products are fortified, but not in Cyprus while the iodine status of the two populations has not previously been extensively investigated [28,29].

Given the gap in literature on the effects of exposure to BPA and the development of TND, the main objective of this pilot case-control study was to investigate the magnitude and variability of human exposures to BPA and its chlorinated derivatives, as well as, BPF and their possible links with the development of thyroid nodules or the alteration of thyroid hormone levels in adult women of Cyprus and Romania. A preliminary analysis of the thyroid nodule characteristics (size and number), including country differences in serum hormones and their relation with the probability of detecting a thyroid nodule was undertaken for this study’s population groups from both Cyprus and Romania [30]. A comprehensive exposure assessment study investigating the links between exposures to BPA, its chlorinated derivatives and BPF and TND is presented here.

## Methods

### Study design, settings and participants

A pilot case-control study was set up in Nicosia, Cyprus and in Cluj-Napoca, Romania using the same recruitment protocol and approach. Participants were females, non-pregnant and were not receiving any treatment for thyroid disorders such as hypothyroidism or hyperthyroidism. Cases were defined as those having thyroid nodules of diameter >3mm diagnosed with ultrasound; recruited at the Archbishop Makarios III Hospital and at the “Ion Chiricuta” Institute of Oncology (IOCN), in Nicosia and Cluj-Napoca, respectively. In Cyprus, controls were randomly recruited from the public sector (contact information is publicly available) and in Romania they were recruited from the university campus nearby the IOCN. Absence of thyroid nodules in controls were confirmed with ultrasound. Recruitment took place from November 2013 until December 2014 in Cyprus and from June 2014 until February 2015 in Romania. All study participants read and signed the informed consent form during their visit at the hospital and the physician’s consultation. In Cyprus, the study was approved by the Cyprus National Bioethics Committee (EEBK/EΠ/2013/27) and in Romania by the IOCN ethics committee (Decision no 16/26.06/2015).

### Data collection and analytical protocols

A questionnaire developed to capture exposures through water consumption and use of consumer products that have been associated with BPA exposures such as drinking water from plastic bottles (20L), canned food, cleaning habits, use of PCPs (shampoo, conditioner, body lotion, shower gel, hair dye, hair foam, hair spray, nail polish, lip care products, face cream), perfume, deodorant or cosmetics (makeup, lipstick, eyeliner, rouge, mascara, makeup remover). The questionnaire was administered to Greek-Cypriot and Romanian women therefore in was written and administered in both Greek and Romanian.

Demographics were also recorded for all participants and anthropometric characteristics (i.e. weight and height) were self-reported. The questionnaires were administered in short interviews after each participant had visited the physician and had signed the consent form. All the questions were translated and formatted in English for the digitization process with the program EpiData (Manager and Entry Client) [31].

#### Thyroid hormone levels and antibodies

Serum thyroid hormone levels of TSH and free thyroxine (fT4) were measured routinely at the hospital during visit for the ultrasound for both cases and controls. In Cyprus, the analysis took place at the laboratories of the Nicosia General Hospital with the IMMULITE 1000 Siemens immunoassay system (Siemens Healthcare Diagnostics, ILL 60015–0778, USA, Abbott kit). In Romania, thyroid hormone levels were measured with Electro-chemiluminescence (ECLIA) (Cobas 6000, Roche kit) at the IOCN.

Antibodies against thyroglobulin (antiTg) and against thyroid peroxidase (antiTPO) were also measured in both populations. Values <40 IU/mL and <56 IU/mL were considered negative for antiTg and antiTPO, respectively in Cyprus. In Romania values <115 IU/mL for antiTg and <34 IU/mL for antiTPO defined the negative status. The determination of antibodies levels took place in the same laboratories and with the same equipment, as described for the hormones.

### Urine sample collection

In Cyprus, all study participants were asked to provide two urine samples using polypropylene urine vials provided by the physician during the consultation. The first sample was a spot sample collected during their visit at the hospital and the second sample was a first morning void brought to the hospital after the consultation with the physician (within 7 days after collection of the first urine sample). The samples were stored in a freezer (-4^°^C) at the hospital until transferred to the laboratory facilities for storage at -80^°^C. In Romania, only one spot sample was collected during the recruitment at IOCN. The samples were kept in deep freezers (in Deltalab medical tubes) until their shipment to Cyprus where they were stored along with the samples of the Cypriot leg of the study at -80^°^C.

### Bioanalytical protocol for the urinary BPA, BPF, ClxBPA and iodine analysis

Except for total (free plus conjugated) BPA, we analyzed urine samples for total BPF and three chlorinated BPA derivatives: 3-chlorobisphenol A (ClBPA), 3,5-dichlorobisphenol A (3,5-Cl_2_BPA) and 3,3’-dichlorobispheno A (3,3’-Cl_2_BPA). The bioanalytical protocol was based upon modifications of our earlier published urinary BPA protocol [32]. Briefly the modifications concerned with the smaller volumes of urine sample and reagents used; 2 mL of urine instead of 4 mL, 1 mL of buffer solution instead of 4 mL, 2.5 mL of solvent extraction mixture instead of 3 mL, and 400 μL of reconstituted solvent instead of 500 μL. The limits of detection (LOD) and in parenthesis limits of quantification (LOQ) were: BPA 10(30) ng/L, BPF 13(39) ng/L, ClBPA 17(52) ng/L, 3,5-Cl_2_BPA 10(30) ng/L, 3,3’-Cl_2_BPA 10(31) ng/L. Spiked quality control recoveries for all five BPA compounds ranged between 90–105% at a concentration of 250 ng/L. With the exception of 3,3’-Cl_2_BPA where 29% of all spot samples and 38% of the morning ones had levels >LOQ, the rest of analytes were quantified in all urine samples (206 spot samples– 121 from Cyprus and 85 Romania–and 114 morning urine samples from Cyprus). Additionally, spot urine samples were analyzed for total iodine following the Centers for Disease Control and Prevention (CDC) Environmental Health protocol [33] using inductively coupled plasma mass spectrometer (ICP-MS) (Thermo X Series II, Thermo Scientific). The LOD for the iodine measurements was 0.28 μg/L and the LOQ 0.83 μg/L. The average recovery of spiked quality controls for iodine was 95(SD 5%) and 122(SD 11%) in synthetic and pooled human urine, respectively. Urinary creatinine was determined by the picric acid-based spectrophotometric method (Jaffe method) [34].

### Statistical analysis

All statistical analyses were performed in R (v. 3.2.3) with RStudio (v. 0.99.489) with the packages: ggplot2, dplyr, stargazer, psych, tableone, sjPlot, ICC [35–43]. Urinary concentration values below the LOD were replaced with ½ LOD and values above LOD and below LOQ were replaced with ½ LOQ. The sum of all chlorinated derivatives of BPA (ClBPA, 3,5-Cl2BPA and 3,3’-Cl2BPA) was calculated and included in the analysis (ClxBPA). Urinary creatinine levels were also measured and used to adjust for urine dilution. Non-normally distributed urinary concentrations and the thyroid hormone levels were log-transformed (natural logarithm) to meet the assumption of normality and used in regression analysis. Between-group comparisons for the continuous variables were conducted with the parametric (t-test) and non-parametric tests (Wilcoxon) for normally and non-normally distributed variables, respectively. Participants’ distribution in the categorical variables were compared with the chi-square test or Fisher’s exact test when there were categories with <5 participants. The answers to the questionnaire on were recoded to variables capturing daily or weekly use to products to be used in the statistical analysis as proxy external exposure measurements.

Associations between urinary BPA, BPF and ClxBPA levels and exposures were explored with linear regression models. The outcome was the log-transformed urinary concentrations and the recoded questionnaire responses referring to product use and everyday activities were used as predictors with various adjustments, such as age, body mass index (BMI), study site or disease status. Logistic regression was performed with disease outcome (having TND or not) as the dependent variable. Participant characteristics and log-transformed samples concentrations were used as predictors in crude models. The concentrations were also used in multivariate models that had been adjusted for age, the thyroid hormone levels and spot urinary iodine.

Possible trends between urinary BPA, BPF and ClxBPA and serum thyroid hormones (TSH and fT4) were also assessed with linear regression models. In the linear regression trend test, each participant’s urinary BPA compound concentration was replaced with the median of the respective quantile and a new continuous predictor was created. The value of the regression coefficient of the predictor was used as a proxy estimator for the trend and its *p-*value was used as the estimator for the *p-*trend value. In all models, the log-transformed urinary BPA, BPF or ClxBPA compound (ng/L) concentrations were used to estimate the median of the quartile and creatinine (g/L, log-transformed) was added as covariate the same processes has been previously used by Omland et al. (2009) [44].

In all regression analyses, urinary concentrations of the chemicals were log-transformed, unless otherwise stated. To account for urine dilution, creatinine levels (also log transformed) were included in the models or the concentrations were used with prior standardization for creatinine levels. Sensitivity analysis included the analyses excluding the values below the 5^th^ and above the 95^th^ percentiles of BPA, BPF and ClxBPA. The reproducibility between spot and first morning voids measurements of BPA compounds was assessed with the calculation of the intraclass correlation coefficient (ICC) (between subject variability/total variability). Additionally, paired t-tests were conducted to assess the differences in the two samples for the whole population and by TND status. Both the ICC and the paired t-tests were conducted using the log-transformed concentrations for the unadjusted and creatinine-adjusted concentrations. ICC ranges from 0 to 1 with values closer to 1 indicating good reproducibility, while lower ICC values denote high within-subject variability. *P-*values < 0.05 were considered statistically significant throughout the analysis.

## Results

### Participant characteristics

In total, 212 women from both Cyprus (*n* = 122) and Romania (*n* = 90) participated in the study, 106 cases and 106 controls. Using data from both countries, females of the control group were younger (47 yrs vs. 52 yrs) with a lower BMI (25 kg/m^2^ vs. 27 kg/m^2^) than those in the case group (Table 1). The majority of participants in both the cases and the controls were non-smokers (76% never smoked and 7% were smoking in the past) and consumed alcohol less than two times weekly. A significantly (*p-*value<0.05) higher percentage of females with elementary level of education was reported for the cases than those for the control group (Table 1). On average controls reported higher frequency of consumption of canned food products, use of PCPs and cosmetics (S1 Table). With regards to differences in exposure habits between the two study locations, water consumption from 20L bottles was mostly reported in Cyprus, where overall higher the weekly cleaning duration (minutes spent cleaning), canned food consumption and frequency of PCPs use were also reported compared to Romania (S1 Table). Overall demographic characteristics of cases and controls were more similar in the study population from Cyprus than the Romanian population where cases and controls significantly differed in age, BMI and education levels (Table 1).

**Table 1:** Descriptive statistics of the case and control group and all the participants (from both study sites).

	Overall				Cyprus				Romania			
	Total (*n* = 212)	Cases (*n =* 106)	Controls (*n =* 106)	*p-*value	Total (*n =* 122)	Cases (*n =* 57)	Controls (*n =* 65)	*p-*value	Total (*n =* 90)	Cases (*n =* 49)	Controls (*n =* 41)	*p-*value
Age (years)*	49±13	52±12	47±13	0.002	51±12	53±12	49±12	0.114	47±13	51±11	42±14	0.001
**BMI (kg/m**^**2**^)*	26.1±5.5	27.4±6.1	24.8±4.5	0.001	25.4±4.6	25.9±4.9	25.0±4.2	0.303	27.0±6.5	29.1±6.9	24.4±4.9	<0.001
**Smoking status**				0.691				0.858				0.677
**Never**	157 (76)	76 (74)	81 (77)		94 (77)	43 (76)	51 (79)		63 (73)	33 (72)	30 (75)	
**Currently**	36 (17)	18 (18)	18 (17)		17 (14)	8 (14)	9 (14)		19 (22)	10 (22)	9 (23)	
**Past**	15 (7)	9 (9)	6 (6)		11 (9)	6 (11)	5 (8)		4 (5)	3 (7)	1 (3)	
**Alcohol consumption**				0.817				0.524				0.364
**Never/Rarely**	175 (85)	88 (86)	87 (83)		95 (78)	47 (83)	48 (74)		80 (94)	41 (91)	39 (98)	
**Weekend**	25 (12)	11 (11)	14 (13)		20 (16)	7 (12)	13 (20)		5 (6)	4 (9)	1 (3)	
**Often**	7 (3)	3 (3)	4 (4)		7 (6)	3 (5)	4 (6)		0 (0)	0 (0)	0 (0)	
**Marital status**				0.017				0.177				0.033
**Single**	25 (12)	6 (6)	19 (18)		13 (11)	4 (7)	9 (14)		12 (14)	2 (4)	10 (25)	
**Married**	157 (76)	85 (83)	72 (69)		99 (81)	49 (86)	50 (77)		58 (67)	36 (78)	22 (55)	
**Divorced**	13 (6)	4 (4)	9 (9)		6 (5)	1 (2)	5 (8)		7 (8)	3 (7)	4 (10)	
**Widow**	12 (6)	7 (7)	5 (5)		4 (3)	3 (5)	1 (2)		8 (9)	4 (9)	4 (10)	
**Education**				0.003				0.134				0.001
**Elementary**	28 (14)	21 (20)	7 (7)		16 (13)	11 (19)	5 (8)		12 (14)	10 (22)	2 (5)	
**Secondary**	78 (38)	40 (39)	38 (37)		43 (35)	17 (30)	26 (40)		35 (41)	23 (50)	12 (31)	
**University**	99 (48)	40 (39)	59 (57)		63 (52)	29 (51)	34 (52)		36 (42)	11 (24)	25 (64)	
** **												
TSH (mIU/L)**	1.6 [1.1, 2.2]	1.42 [0.90, 1.97]	1.68 [1.18, 2.49]	0.016	1.51 [1.06, 2.00]	1.40 [0.90, 1.98]	1.53 [1.08, 2]	0.559	1.68 [1.05, 2.55]	1.44 [0.83, 1.85]	2.20 [1.28, 2.94]	0.003
fT4 (pmol/L)**	13.4 [12.1, 14.9]	13.40 [12.40, 14.90]	13.43 [12.06, 14.90]	0.748	12.70 [11.80, 14]	12.90 [11.90, 13.95]	12.60 [11.80, 14]	0.606	14.71 [13.10, 16.10]	14.60 [12.90, 15.70]	14.80 [13.50, 16.10]	0.598
Spot iodine (μg/L)**	108 [49, 186]	106 [44, 158]	111 [54, 190]	0.32	94 [40, 190]	82 [27, 166]	103 [48, 190]	0.254	118 [61, 183]	116 [64, 153]	128 [59, 189]	0.583
** **												
BPA (ng/L)**	2258 [1100, 4611]	1751 [1117, 3458]	2710.50 [1081, 5912]	0.016	1508 [789, 2806]	1394 [757, 2189]	1832 [792, 3004]	0.333	3778 [2134, 9050]	2453 [1495, 5173]	8013 [3112, 19288]	<0.001
BPF (ng/L)**	465 [320, 720]	463 [338, 680]	480 [287, 788]	0.703	485 [365, 680]	457 [378, 634]	510 [360, 761]	0.54	416 [219, 822]	492 [256, 822]	371 [159, 1035]	0.32
ClxBPA (ng/L)**	159 [147, 187]	159 [144, 184]	159 [148, 187]	0.757	152 [141, 168]	150 [140, 167]	153 [144, 169]	0.357	177 [158, 201]	178 [158, 201]	177 [161, 201]	0.860

The normally (*) distributed and non-normally (**) distributed continuous variables are presented with the mean± SD or median[IQR], respectively. Categorical variables are presented with *n*(%). T-tests were used for the mean comparisons of the normally distributed continuous variables and Wilcoxon test for the non-normally distributed. Chi-square tests and Fisher’s exact test were used in the categorical variables (the latter when the categories included <5 subjects). Abbreviations: BPA: bisphenol A, BPF: bisphenol F; ClxBPA: sum of 3-chlorobisphenol A (ClBPA), 3,5-dichlorobisphenol A (3,5-Cl2BPA) and 3,3’-dichlorobispheno A (3,3’-Cl2BPA); BMI: body mass index; TSH: thyroid stimulating hormone; fT4: free thyroxine.

### Thyroid hormones and spot iodine levels

In the whole study population, median serum TSH was significantly (*p-*value<0.05) lower in cases than in controls (1.42 mIU/L vs. 1.68 mIU/L) (Table 1), but within the normal reference ranges. Median serum fT4 levels did not differ between cases and controls (13.40 pmol/L vs. 13.43 pmol/L), while spot median urinary iodine levels also did not differ between cases and controls (105.65 μg/L vs. 110.60 μg/L), denoting adequate iodine status for the whole study population (Table 1). No difference between cases and controls for urinary iodine and fT4 were observed within each country, while median serum TSH levels was higher in controls than cases in Romania (1.44 mIU/L and 2.2 mIU/L, TSH median for cases and controls, respectively).

In Cyprus, all participants were within the normal TSH range (0.4–4 mIU/L) whereas two cases and 2 controls were below the reference fT4 range (10–24 pmol/L). TSH and fT4 levels were in the normal range for the majority of the participants from Romania. In total, 10 participants (4 cases and 6 controls) were above the upper cutoff for TSH (0.27–4.2 mIU/L) and 3 (2 cases and 1 control) were below the lower cutoff of fT4 (10.6–22.7 pmol/L). Of the available information on the antibody levels, 95.5% of the cases and 88.7% of the controls had negative antiTg. AntiTPO was negative for 83.8% and 70.8% of the cases and controls, respectively (S2 Table).

### Urinary BPA, BPF and ClxBPA levels

In the pooled country dataset, median urinary BPA levels were significantly (*p*<0.05) higher for controls (2710 ng/L vs. 1751 ng/L), albeit no difference in the urinary BPF or ClxBPA levels between cases and controls was observed (Table 1). The Romanian population had significantly higher BPA levels (median [interquartile range (IQR)] 3778 ng/L [2134, 9050] and 4670 ng/g creatinine [2231, 10050], when compared to the Cypriot population (median [IQR]: 1508 [789, 2806] ng/L and 2101 [1184, 4220] ng/g of creatinine) (S3 Table). Median urinary BPF levels were higher in the Cypriot population (485 [365, 680] ng/L and 646 [398, 1327] ng/g) when compared to those of the Romanian population (416 [219, 822] ng/L and 500 [271, 1138] ng/g) (S3 Table). The sum of urinary ClxBPA levels was slightly higher in Romania (177 [158, 201] ng/L) than that in Cyprus (152 [141, 168] ng/L) (S3 Table).

The estimated ICC indicated low reproducibility (0.16) between the two urinary BPA measurements for the Cypriot population (S4 Table). Creatinine-unadjusted BPF levels had the highest ICC value (0.46) showing better reproducibility between the spot and the morning levels when compared to ICC estimates of BPA or ClxBPA (S4 Table).

After adjustment for covariates like urinary creatinine, diseases status, age BMI and study site, linear regression showed PC bottle water used and frequency of PCP used as significant (p<0.05) predictors of spot urinary BPA (Table 2). Similarly, frequency of microwave use was the single significant predictor of urinary BPF levels for both countries, while microwave use, PCP and cosmetics use were driving the relation with urinary ClxBPA, after accounting for possible confounders (Table 2). In the sensitivity analysis only PCP use retained its significance as a predictor of urinary BPA levels (S5 Table).

**Table 2:** Linear regression analysis of the exposure habits as determinants of spot urinary BPA levels (creatinine unadjusted, log-transformed) for the pooled study population.

	**BPA**			
	Model 1*		Model 2**	
	**β coefficient (95% CI)**	***p-*value**	**β coefficient (95% CI)**	***p-*value**
**20L Bottled water use (glasses per day)**	0.038 (-0.036, 0.111)	0.315	0.075 (0.002, 0.147)	0.046
**Microwave oven use (times per week)**	-0.049 (-0.125, 0.027)	0.208	-0.031 (-0.101, 0.039)	0.386
**Canned food consumption (portion per week)**	-0.015 (-0.070, 0.039)	0.579	-0.013 (-0.063, 0.036)	0.598
**Cleaning duration (mins per week)**	-0.001 (-0.002, -0.001)	0.0004	-0.001 (-0.001, 0.0001)	0.098
**PCPs use (number of times using a product per week)**	-0.003 (-0.007, 0.002)	0.219	0.005 (0.001, 0.010)	0.03
**Perfume use (times per week)**	0.006 (-0.036, 0.047)	0.785	-0.024 (-0.064, 0.015)	0.226
**Deodorant use(times per week)**	0.034 (-0.010, 0.078)	0.128	0.013 (-0.027, 0.053)	0.532
**Cosmetics use (times per week)**	0.009 (-0.002, 0.020)	0.129	0.003 (-0.008, 0.014)	0.599
** **	**BPF**			
** **	Model 1*		Model 2**	
** **	**β coefficient (95% CI)**	***p-*value**	**β coefficient (95% CI)**	***p-*value**
**20L Bottled water use (glasses per day)**	0.005 (-0.068, 0.078)	0.886	0.001 (-0.085, 0.086)	0.989
**Microwave oven use (times per week)**	0.124 (0.050, 0.198)	0.002	0.142 (0.062, 0.221)	0.001
**Canned food consumption (portion per week)**	-0.006 (-0.059, 0.047)	0.821	-0.008 (-0.065, 0.048)	0.776
**Cleaning duration (mins per week)**	0.00001 (-0.001, 0.001)	0.966	0.0001 (-0.001, 0.001)	0.821
**PCPs use (number of times using a product per week)**	0.002 (-0.002, 0.006)	0.375	0.002 (-0.004, 0.007)	0.542
**Perfume use (times per week)**	-0.014 (-0.055, 0.027)	0.502	-0.024 (-0.069, 0.022)	0.31
**Deodorant use (times per week)**	0.006 (-0.039, 0.050)	0.806	0.004 (-0.043, 0.052)	0.854
**Cosmetics use (times per week)**	-0.0001 (-0.011, 0.011)	0.983	-0.002 (-0.015, 0.011)	0.752
** **	**ClxBPA**			
** **	Model 1*		Model 2**	
** **	**β coefficient (95% CI)**	***p-*value**	**β coefficient (95% CI)**	***p-*value**
**20L Bottled water use (glasses per day)**	0.006 (-0.009, 0.021)	0.461	0.016 (-0.0002, 0.032)	0.055
**Microwave oven use (times per week)**	-0.021 (-0.036, -0.005)	0.009	-0.016 (-0.031, -0.001)	0.04
**Canned food consumption (portion per week)**	-0.002 (-0.014, 0.009)	0.7	-0.001 (-0.012, 0.011)	0.916
**Cleaning duration (mins per week)**	-0.00003 (-0.0002, 0.0001)	0.717	-0.00004 (-0.0002, 0.0001)	0.615
**PCPs use (number of times using a product per week)**	-0.0002 (-0.001, 0.001)	0.622	0.001 (-0.0001, 0.002)	0.071
**Perfume use (times per week)**	0.003 (-0.005, 0.012)	0.462	0.001 (-0.007, 0.010)	0.741
**Deodorant use(times per week)**	0.001 (-0.008, 0.010)	0.851	0.0001 (-0.009, 0.009)	0.988
**Cosmetics use (times per week)**	0.002 (0.0001, 0.005)	0.046	0.003 (0.001, 0.006)	0.01

*Crude estimate (only adjustment for log-creatinine has been included)

**Adjusted models for: log-creatinine, study site, disease status, age and BMI

Abbreviations: BPA: bisphenol A, BPF: bisphenol F; ClxBPA: sum of 3-chlorobisphenol A (ClBPA), 3,5-dichlorobisphenol A (3,5-Cl_2_BPA) and 3,3’-dichlorobispheno A (3,3’-Cl_2_BPA); PCPs: personal care products, CI: confidence intervals

In crude logistic regression analysis, age and BMI were significant predictors of higher odds of thyroid nodules (S6 Table). Urinary BPA (ng/L) was also significant in the univariate analysis with an odds ratio (OR) of 0.56 (95% CI: 0.40, 0.77) (S7 Table). In the multivariate logistic regression models of TND adjusting for age, BMI, thyroid hormones’ levels, spot iodine concentrations (with and without study site), BPA resulted in OR of TND below 1 (Table 3). In the adjusted for creatinine only models, the OR of thyroid nodules ranged from a low of 0.67 (95% CI, 0.50, 0.88) to 0.96 (95% CI, 0.73, 1.24) per log unit increase in urinary BPA and BPF, respectively (Table 3). Urinary BPA levels were higher in the controls than in the cases and its association with TND held after adjusting for several covariates (Table 3); upon constraining the logistic regression analysis to the 5^th^ - 95^th^ percentile of the populations, this association did not hold (S8 Table).

**Table 3:** Odds ratios of having thyroid nodules and urinary BPF, BPA and ClxBPA adjusted for age, BMI, hormone levels (A1) and iodine (B1). The two sets of models were repeated with the inclusion of the study site as a covariate (A2, B2).

	**Crude estimates**		**A1**		**A2**	
	**OR (95%CI)**	***p*-value**	**OR (95%CI)**	***p*-value**	**OR (95%CI)**	***p*-value**
**BPA (ng/L)**	0.67 (0.50 – 0.88)	0.006	0.76 (0.56, 1.03)	0.085	0.65 (0.46, 0.91)	0.014
**BPF (ng/L)**	0.96 (0.73, 1.24)	0.731	0.98 (0.73, 1.31)	0.902	0.99 (0.74, 1.32)	0.939
**ClxBPA (ng/L)**	0.78 (0.21, 2.78)	0.706	0.95 (0.22, 3.88)	0.941	0.7 (0.14, 3.09)	0.647
			**B1**		**B2**	
			**OR (95%CI)**	***p*-value**	**OR (95%CI)**	***p*-value**
**BPA (ng/L)**			0.8 (0.58, 1.08)	0.148	0.67 (0.47, 0.94)	0.025
**BPF (ng/L)**			0.98 (0.73, 1.30)	0.867	0.98 (0.73, 1.31)	0.9
**ClxBPA (ng/L)**			1.07 (0.24, 4.47)	0.924	0.77 (0.15, 3.47)	0.744

Note: All concentrations are log-transformed and all models have been adjusted for log-transformed creatinine. Model details: Crude models have been only adjusted for log-creatinine (g/L); (A1) Adjusted for: age, BMI, TSH and FT4; (A2) Adjusted for: study site, age, BMI, TSH and FT4; (B1) Adjusted for: age, BMI, TSH, FT4 and spot iodine (μg/L); (B2) Adjusted for: study site, age, BMI, TSH, FT4 and spot iodine (μg/L). Abbreviations: BPA: bisphenol A, BPF: bisphenol F; ClxBPA: sum of 3-chlorobisphenol A (ClBPA), 3,5-dichlorobisphenol A (3,5-Cl_2_BPA) and 3,3’-dichlorobispheno A (3,3’-Cl_2_BPA); OR: odds ratio; CI: confidence intervals.

A consistently positive and significant association was observed between urinary BPA and serum TSH that remained after models were adjusted for various covariates such as urinary creatinine, age, BMI, study site and disease status (Table 4); there was not a significant relationship for either BPF or ClxBPA with serum TSH. The significant association between BPA and TSH remained even after the sensitivity test using only the 5^th^– 95^th^ percentile study group in all models but the one adjusted for TND status (along with study site, age and BMI) (Table 4). No significant association between any of the BPA compounds and serum fT4 was observed (S9 Table).

**Table 4:** Test for trend in multivariate models adjusted for age, BMI, study site and disease status (creatinine adjustment has been included in all models) for the whole study population and including only those between the 5^th^ and 95^th^ percentile of BPA, BPF and ClxBPA (ng/L) urinary concentrations.

	Whole study population			Sensitivity analysis	
** **	**(A)**	** **	** **	**(A)**	** **
** **	**β coefficient (95% CI)**	***p-*value**	** **	**β coefficient (95% CI)**	***p-*value**
**BPA (ng/L)**	0.145 (0.045, 0.246)	0.006	**BPA (ng/L)**	0.125 (0.009, 0.240)	0.036
**BPF (ng/L)**	-0.063 (-0.211, 0.085)	0.404	**BPF (ng/L)**	-0.079 (-0.240, 0.082)	0.337
**ClxBPA (ng/L)**	0.095 (-0.443, 0.633)	0.73	**ClxBPA (ng/L)**	0.074 (-0.520, 0.667)	0.809
	**(B)**			**(B)**	
** **	**β coefficient (95% CI)**	***p-*value**	** **	**β coefficient (95% CI)**	*p-*value
**BPA (ng/L)**	0.144 (0.041, 0.248)	0.007	**BPA (ng/L)**	0.124 (0.007, 0.242)	0.04
**BPF (ng/L)**	-0.033 (-0.186, 0.119)	0.669	**BPF (ng/L)**	-0.045 (-0.211, 0.121)	0.594
**ClxBPA (ng/L)**	0.151 (-0.400, 0.703)	0.592	**ClxBPA (ng/L)**	0.036 (-0.570, 0.641)	0.909
	**(C)**	** **		**(C)**	** **
	**β coefficient (95% CI)**	***p-*value**	** **	**β coefficient (95% CI)**	***p-*value**
**BPA (ng/L)**	0.146 (0.032, 0.260)	0.013	**BPA (ng/L)**	0.130 (0.004, 0.255)	0.045
**BPF (ng/L)**	-0.024 (-0.177, 0.129)	0.759	**BPF (ng/L)**	-0.043 (-0.211, 0.125)	0.618
**ClxBPA (ng/L)**	** **0.047 (-0.550, 0.644)	0.879	**ClxBPA (ng/L)**	-0.034 (-0.695, 0.627)	0.919
	**(D)**	** **		**(D)**	** **
** **	**β coefficient (95% CI)**	***p-*value**	** **	**β coefficient (95% CI)**	***p-*value**
**BPA (ng/L)**	0.121 (0.008, 0.234)	0.037	**BPA (ng/L)**	0.113 (-0.011, 0.237)	0.076
**BPF (ng/L)**	-0.014 (-0.164, 0.135)	0.853	**BPF (ng/L)**	-0.031 (-0.195, 0.134)	0.718
**ClxBPA (ng/L)**	0.019 (-0.564, 0.602)	0.95	**ClxBPA (ng/L)**	-0.091 (-0.741, 0.560)	0.786

Model details: (A) Adjusted for: creatinine (g/L, log-transformed); (B) Adjusted for: age, BMI and creatinine (g/L, log-transformed); (C) Adjusted for: age, BMI, study site[Romania] and creatinine; (D) Adjusted for: age, BMI, study site[Romania], disease status[case] (and creatinine). Abbreviations: BPA: bisphenol A, BPF: bisphenol F; ClxBPA: sum of 3-chlorobisphenol A (ClBPA), 3,5-dichlorobisphenol A (3,5-Cl_2_BPA) and 3,3’-dichlorobispheno A (3,3’-Cl_2_BPA); OR: CI: confidence intervals.

## Discussion

The present two-site pilot study explored the association between urinary BPA, BPF and ClxBPA and thyroid nodules or the hormones TSH and fT4 among adult females using a case-control study design; BPA exposures were linked with lower odds of having thyroid nodules although this trend did not hold when outliers were removed. In both country populations, multiple nodules were found on thyroid gland that were localized in both lobes of the gland, being 65% of all thyroids in Romania and 80% in Cyprus [30]. This study’s findings provided evidence that ubiquitous exposures to BPA were associated with alterations in circulating TSH levels of adult females in Cyprus and Romania. Data provided evidence of a significant positive trend between spot urinary BPA levels and TSH, even after adjusting for possible confounders, including spot urinary iodine, disease status, creatinine, BMI and age; the significance did not hold for BPF or ClxBPA.

Human exposures to BPA and its effects on thyroid measures, as well as on the development of thyroid nodules have not been widely assessed in epidemiologic studies. Possible thyroid disrupting properties of BPA have been mostly studied in vitro or in animal studies [8]. Our results on thyroid measures are mostly in accordance with other studies looking at BPA effects on peripheral thyroid hormone function. However, the majority of our study population was within the normal range of TSH and this association has not yet been established in the literature. In effect, a positive association between urinary BPA and serum TSH levels were observed in a U.S. NHANES representative general population adult group (2007–2008), albeit not significant (*p* = 0.2) [22]. A recent study in Belgium (*n =* 194) showed a significant (*p* = 0.026) positive association between exposures to BPA and serum TSH levels in a lean (BMI < 25 kg/m^2^) adult group; the trend seemed to be particularly driven by females [45]. On the contrary, a decrease in serum TSH was observed for both male and female adults with increases in urinary BPA levels [24].

Besides BPA, other ubiquitous thyroid disrupting chemicals, such as perchlorate, certain phthalates and perfluoroalkyl acids (PFOAs) have been linked with perturbations in thyroid hormonal function. Using data from a U.S. population representative adult group (*n =* 1346), Meeker and Ferguson (2011) showed significant positive associations between serum TSH and urinary di(2-ethylhexyl) phthalate (DEHP) metabolites, and a negative association with total T3, suggesting direct influence of DEHP metabolites on thyroid hormonal synthesis, metabolism and thyroid hormonal receptor activation [22]. Similar patterns of effects on thyroid hormonal measures were also observed in another human study on PFOAs from the NHANES (2007–2008) dataset [46]. An interquartile range increase in PFOAs resulted in an increase of about 30% in serum TSH levels for those adults with both inadequate iodine status (<100μg/L urinary iodine) and high thyroid peroxidase antibody levels [46].

The fact that higher urinary BPA levels were measured in the control group could be perhaps attributed to specific dietary habits and/or exposure sources. After adjustments for creatinine, study site, disease status, age and BMI, the daily consumption of water from 20L bottles and the frequency of use of PCPs were significant predictors of urinary BPA levels. When the tests were repeated by disease status, consumption of water from 20L bottles and frequency of PCPs use were also significant predictors of BPA levels in the cases but not in the controls (details available in the Supporting Information).

Our study did not find an association between exposures to BPA compounds and the incidence of thyroid nodules. No population study was available on the association between BPA compounds and the risk of developing thyroid nodules. Interestingly, cases with thyroid nodules had lower urinary BPA levels than their healthy counterparts; however, the lower odds per log unit increase in urinary BPA compounds did not reach the level of significance upon outlier exclusion and adjusting for possible confounders. Our results were in accordance with a children’s health study (*n =* 718) in China showing an inverse association of urinary BPA with the risk of multinodular thyroid disease [20].

The magnitude of human exposures to BPA was comparable, if not higher than that typically reported in the literature. Median urinary BPA levels in our population groups from Cyprus and Romania were 1.5 μg/L (2.1 μg/g) and 3.8 μg/L (4.7 μg/g), respectively. Earlier BPA biomonitoring study in Cyprus (*n =* 224 adults) matched the median urinary BPA levels observed here (2.1 μg/g) [47]. A recent biomonitoring analysis by CDC in the U.S. by pooling urine samples from available NHANES datasets using the corrected NHANES sampling weights showed a decline in the creatinine-adjusted median urinary BPA levels from 2.58 μg/g in 2003, reaching 1.91 μg/g by 2010 [48]. Urinary BPA data were generated for the first time in Romania, being above the typically reported median BPA levels in other countries.

This study showed higher urinary BPF levels in both countries [GM: 548 ng/L (775 ng/g creatinine) and 523 ng/L (637 ng/g) in Cyprus and Romania, respectively] than those reported for an adult group (*n =* 100) in the U.S. where BPF was found in 55% of all urine samples, at concentrations up to 212 ng/mL [49]. At eight time points between 2000 and 2014, BPF was detected at the frequency of 42–88% with a range of GM concentrations (0.15–0.54 ng/mL) in archived urine samples of U.S. adults (*n =* 616) [18]. BPF has recently attracted attention given its widespread use as a BPA substitute and its safety has been questioned [50,51]. Information on the determinants of exposure to BPF is lacking in environmental epidemiological studies. This study showed 100% BPF detection rate, showing microwave use as an important predictor of its urinary levels, warranting further assessment of its exposures. Compared to urinary BPA or ClxBPA, urinary BPF showed the highest reproducibility estimates between the spot and morning samples.

This study was limited by its small sample size that did not allow to generalize our results for the two populations. Other limitations include the fact that exposure habits were assessed through questions that were not exhaustive and could not include all possible sources of exposure. The spot sample collected might not be representative of the exposure to the measured BPA compounds corroborated by the ICC data showing limited reproducibility between the morning and spot samples suggesting possible exposure misclassification. The study did not include all thyroid hormones that are implicated in thyroid function and therefore possible links between hormones such as T3 or total T4 could not be investigated.

## Conclusions

With the exception of a chlorinated BPA compound (30%), the rest of BPA compounds were found at concentrations above LOQ for 100% of urine samples collected from both country sites in Cyprus and Romania. Median urinary BPA levels were nearly double in Romania than those measured in Cyprus. Urinary BPF levels measured in both study sites were higher than those reported in the literature. There was a positive association of BPA with TSH, thus, such an association with serum TSH was not detected for BPF or for the ClxBPA compounds. No association between exposures to bisphenols and serum ft4 was observed. Urinary BPA levels adjusted for possible confounders were higher in the controls than in the cases and OR for TND were <1, but this association was not seen when outliers were removed.

The effect of thyroid disrupting chemicals, like BPA on altering thyroid measures (serum TSH) has not been established yet and it needs to be further investigated in larger scale. The possible association between BPA levels and thyroid health indicators may have important public health implications in relation with thyroid disorders, such as hypothyroidism for those with borderline low fT4 or high TSH levels. At the individual level, discrete alterations in serum TSH levels may seem insignificant if compared against the reference range. However, there is a narrow window of regulation for TSH, maintaining an individual’s set point of homeostasis [8]. Deviations from this narrow window of euthyroid hormonal function could result in alterations in thyroid hormonal status; such tight deviations form an individual’s set point of thyroid homeostasis may not be detected in small human studies. Larger studies are needed to better delineate the effects of thyroid disrupting chemicals on thyroid function with particular emphasis on critical life stages and susceptible population groups.

## Supporting Information

S1 Table Summary of exposure habits between the two study locations and between cases and controls. (PDF)

S2 Table Overview of the positive and negative antibody status for all the study participants. (PDF)

S3 Table Distribution of the urinary BPA, BPF and ClxBPA levels between the two countries. (PDF)

S4 Table Intraclass correlation coefficient (and 95% CIs) and paired t-tests for the log-transformed values of BPA, BPF and ClxBPA (Cyprus). (PDF)

S5 Table Results of the sensitivity analysis for the determinants of urinary BPA, BPF and ClxBPA levels. (PDF)

S6 Table Odds ratios of the univariate logistic regression with participants’ characteristics as predictors (A. models have only each characteristic as predictor; B. models have been adjusted for the study site).
(PDF)

S7 Table Odds ratios of the urinary levels of BPF, BPA and ClxBPA from logistic regression adjusted for the study site. (PDF)

S8 Table Sensitivity analysis results of the multivariate logistic regression models. (PDF)

S9 Table Test for trend in multivariate models where the outcome is log-transformed ft4, adjusted for age, BMI, study site and disease status (creatinine adjustment has been included in all models) for the whole study population. (PDF)

S10 Table Linear regression analysis of the exposure habits as determinants of spot urinary BPA levels (creatinine unadjusted, log-transformed) for the cases and controls, separately. (PDF)

S11 Table Raw data (csv format). (CSV)

S12 Table Raw data (csv format). (CSV)

S13 Table Raw data (csv format). (CSV)

S14 Table Raw data (csv format). (CSV)

S15 Table Raw data (csv format). (CSV)

S16 Table Raw data (csv format). (CSV)

S17 Table Raw data (csv format). (CSV)

S18 Table R markdown file with the analysis script. (RMD)

S19 Table Report from the statistical analysis. (HTML)
