# Supplementary material for: Human Exposures to Bisphenol A, Bisphenol F and Chlorinated Bisphenol A Derivatives and Thyroid Function
Source: PLoS One. 2016 Oct 26;11(10):e0155237. doi: 10.1371/journal.pone.0155237 (PMC5082639; doi:10.1371/journal.pone.0155237)
Supplement: S1 Table — (PDF) [file pone.0155237.s001.pdf]

Table S1. Summary of exposure habits between the two study locations and between cases and controls.

	Total (*n*=212)			Cyprus (n=122)			Romania (n=90)		
	**Overall**	**Cases**	**Controls**	**Overall**	**Cases**	**Controls**	**Overall**	**Cases**	**Controls**
n	**212**	**106**	**106**	**122**	**57**	**65**	**90**	**49**	**41**
**Daily consumption of water from 20L bottles (glasses; 1 glass=250mL)**	0.00 [0.00, 0.00]	0.00 [0.00, 0.00]	0.00 [0.00, 0.00]	0.00 [0.00, 0.91]	0.00 [0.00, 0.00]	0.00 [0.00, 2.14]	0.00 [0.00, 0.00]	0.00 [0.00, 0.00]	0.00 [0.00, 0.00]
**Frequency of use of the microwave oven (times per week)**	0.00 [0.00, 0.00]	0.00 [0.00, 0.00]	0.00 [0.00, 0.00]	0.00 [0.00, 0.00]	0.00 [0.00, 0.00]	0.00 [0.00, 0.02]	0.00 [0.00, 0.00]	0.00 [0.00, 0.00]	0.00 [0.00, 0.00]
**Consumption of canned food (portion per week)**	1.25 [0.38, 2.75]	1.00 [0.27, 2.22]	1.50 [0.53, 3.50]	1.88 [0.75, 3.01]	1.77 [0.70, 2.28]	2.25 [0.87, 3.81]	0.50 [0.00, 2.00]	0.38 [0.00, 1.12]	0.75 [0.12, 2.50]
**Cleaning duration (mins per week)**	277.50 [161.25, 427.50]	292.50 [173.12, 495.00]	262.50 [142.00, 396.12]	308.62 [177.50, 504.38]	321.16 [202.81, 524.75]	302.40 [171.25, 446.25]	211.25 [115.00, 355.00]	240.00 [102.50, 399.88]	174.75 [115.00, 306.25]
**Frequency of use of PCPs (number of times using a product per week)**	70.00 [53.75, 96.25]	65.72 [41.69, 91.69]	74.38 [58.00, 97.58]	86.20 [65.86, 103.00]	81.35 [65.59, 103.12]	86.76 [67.14, 102.75]	50.50 [32.50, 65.00]	41.12 [25.12, 59.00]	58.00 [51.50, 68.75]
**Frequency of perfume use (times per week)**	7.00 [1.00, 7.00]	5.00 [0.00, 7.00]	7.00 [3.50, 7.00]	7.00 [1.50, 7.00]	5.00 [0.75, 7.00]	7.00 [2.00, 7.00]	7.00 [1.00, 7.00]	4.50 [0.00, 7.00]	7.00 [5.00, 7.00]
**Frequency of use of deodorant (times per week)**	7.00 [2.88, 7.00]	7.00 [0.00, 7.00]	7.00 [7.00, 7.00]	7.00 [3.25, 7.00]	7.00 [1.00, 7.00]	7.00 [4.25, 7.00]	7.00 [3.00, 7.00]	7.00 [0.00, 7.00]	7.00 [7.00, 7.00]
**Frequency of use of cosmetics (times of using the products per week)**	7.00 [0.00, 22.06]	3.88 [0.00, 15.75]	14.00 [1.00, 28.00]	7.00 [0.18, 22.25]	6.62 [0.02, 16.88]	9.00 [1.00, 22.88]	7.00 [0.00, 21.12]	1.00 [0.00, 13.00]	20.50 [2.00, 28.00]

Median [IQR] values are presented in all variables

Abbreviations: PCPs: personal care products
