# Supplementary material for: Human Exposures to Bisphenol A, Bisphenol F and Chlorinated Bisphenol A Derivatives and Thyroid Function
Source: PLoS One. 2016 Oct 26;11(10):e0155237. doi: 10.1371/journal.pone.0155237 (PMC5082639; doi:10.1371/journal.pone.0155237)
Supplement: S2 Table — (PDF) [file pone.0155237.s002.pdf]

Table S2. Overview of the positive and negative antibody status for all the study participants.

	Overall					
	**Total number of subjects**	**missing**	**% missing**		***n***	**%**
**antiTg**	212	83	39.2	**Negative**	119	92.2
				**Positive**	10	7.8
**antiTPO**	212	73	34.4	**Negative**	108	77.7
				**Positive**	31	22.3
	**Cases**					
**antiTg**	106	39	36.8	**Negative**	84	95.5
				**Positive**	3	4.5
**antiTPO**	106	32	30.2	**Negative**	62	83.8
				**Positive**	12	16.2
	**Controls**					
**antiTg**	106	44	41.5	**Negative**	55	88.7
				**Positive**	7	11.3
**antiTPO**	106	41	38.7	**Negative**	46	70.8
				**Positive**	19	29.2
	**Cyprus**					
	**Total number of subjects**	**Missing**	**% missing**		***n***	**%**
**antiTg**	122	63	51.6	**Negative**	56	94.9
				**Positive**	3	5.1
**antiTPO**	122	61	50	**Negative**	52	85.2
				**Positive**	9	14.8
	**Cyprus**					
	**Cases**	**Missing**	**% missing**		***n***	**%**
**antiTg**	57	30	52.6	**Negative**	26	96.3
				**Positive**	1	3.7
**antiTPO**	57	28	49.1	**Negative**	28	96.6
				**Positive**	1	3.4
	**Controls**	**Missing**	**% missing**		***n***	**%**
**antiTg**	65	33	50.8	**Negative**	30	93.8
				**Positive**	2	6.2
**antiTPO**	65	33	50.8	**Negative**	24	75.0
				**Positive**	8	25.0
	**Romania**					
	**Total number of subjects**	**missing**	**% missing**		***n***	**%**
**antiTg**	90	20	22.2	**Negative**	63	90

				**Positive**	7	10
**antiTPO**	90	12	13.3	**Negative**	56	71.8
				**Positive**	22	28.2
	**Cases**				***n***	**%**
**antiTg**	49	9	18.4	**Negative**	38	95
				**Positive**	2	5
**antiTPO**	49	4	8.2	**Negative**	34	75.6
				**Positive**	11	24.4
	**Controls**				***n***	**%**
**antiTg**	41	11	26.8	**Negative**	25	83.3
				**Positive**	5	16.7
**antiTPO**	41	8	19.5	**Negative**	22	66.7
				**Positive**	11	33.3
