# Supplementary material for: Human Exposures to Bisphenol A, Bisphenol F and Chlorinated Bisphenol A Derivatives and Thyroid Function
Source: PLoS One. 2016 Oct 26;11(10):e0155237. doi: 10.1371/journal.pone.0155237 (PMC5082639; doi:10.1371/journal.pone.0155237)
Supplement: S3 Table — (PDF) [file pone.0155237.s003.pdf]

Table S3. Distribution of the urinary BPA, BPF and ClxBPA levels between the two countries.

	**Cyprus**	**Romania**	***p*-value**
N	122	90	
**BPA (ng/L)**	1508 [789, 2806]	3778 [2134, 9050]	<0.001
**BPF (ng/L)**	485 [365, 680]	416 [219, 822]	0.091
**ClxBPA (ng/L)**	152 [141.00, 168.00]	177 [158, 201]	<0.001
**Creatinine-adjusted concentrations**			
**BPA (ng/g)**	2101 [1184, 4220]	4670 [2231, 10050]	<0.001
**BPF (ng/g)**	646 [398, 1327]	500 [271, 1138]	0.014
**ClxBPA (ng/g)**	199.19 [120, 376]	201 [125, 394]	0.961

Median[IQR] values are presented along with the *p*-value of the Wilcoxon test.

Abbreviations: BPA: bisphenol A, BPF: bisphenol F; ClxBPA: sum of 3-chlorobisphenol A (ClBPA), 3,5-dichlorobisphenol A (3,5-Cl2BPA) and 3,3-dichlorobispheno A (3,3’-Cl2BPA)

.
