# Supplementary material for: Human Exposures to Bisphenol A, Bisphenol F and Chlorinated Bisphenol A Derivatives and Thyroid Function
Source: PLoS One. 2016 Oct 26;11(10):e0155237. doi: 10.1371/journal.pone.0155237 (PMC5082639; doi:10.1371/journal.pone.0155237)
Supplement: S4 Table — (PDF) [file pone.0155237.s004.pdf]

Table S4. Intraclass correlation coefficient (and 95% CIs) and paired t-tests for the log-transformed values of BPA, BPF and ClxBPA (Cyprus).

	Sample type	*n*	GM	GSD	Mean	SD	Median	25th percentile	75th percentile	Paired t-test	ICC	2.5% CI	97.5% CI
**BPA (ng/L)**	**Spot**	121	1575	2.5	2495	3295	1508	789	2806	<0.001	0.16	-0.03	0.34
	**Morning**	114	2727	3	11216	62924	2208	1457.5	4632				
**BPF (ng/L)**	**Spot**	121	548	2.3	3181	27304	485	365	680	0.063	0.46	0.31	0.60
	**Morning**	114	637	2.3	1404	5833	550	402.2	878				
**ClxBPA (ng/L)**	**Spot**	121	160	1.2	164	39	152	141	168	<0.001	0.09	-0.09	0.28
	**Morning**	114	182	1.4	194	104	164	150	203				
**BPA (ng/g)**	**Spot**	121	2229	2.5	3380	3626	2101	1184.3	4220	<0.001	0.21	0.03	0.39
	**Morning**	112	3923	3.5	26119	172516	3545	1595.6	6885				
**BPF (ng/g)**	**Spot**	121	775	2.6	2130	11351	646	397.8	1327	0.280	0.21	0.03	0.39
	**Morning**	112	897	3	2236	6048	815	445	1219				
**ClxBPA (ng/g)**	**Spot**	121	227	2.3	335	365	199	120.2	376	0.273	0.32	0.15	0.48
	**Morning**	112	259	2.4	514	1760	228	127.3	403				
