# Supplementary material for: Human Exposures to Bisphenol A, Bisphenol F and Chlorinated Bisphenol A Derivatives and Thyroid Function
Source: PLoS One. 2016 Oct 26;11(10):e0155237. doi: 10.1371/journal.pone.0155237 (PMC5082639; doi:10.1371/journal.pone.0155237)
Supplement: S5 Table — (PDF) [file pone.0155237.s005.pdf]

Table S5. Results of the sensitivity analysis for the determinants of urinary BPA, BPF and ClxBPA levels.

	BPA (ng/L)		BPF (ng/L)		ClxBPA (ng/L)	
	β coefficient (95% CI)	p-value	β coefficient (95% CI)	p-value	β coefficient (95% CI)	p-value
**20L Bottled water use (glasses per day; 1 glass=250mL)**	0.056 (-0.002, 0.114)	0.062	0.022 (-0.027, 0.071)	0.375	0.013 (0.002, 0.024)	**0.017**
**Microwave oven use (times per week)**	-0.022 (-0.077, 0.032)	0.426	0.056 (-0.003, 0.115)	0.067	-0.010 (-0.021, 0.0004)	0.062
**Canned food consumption (portion per week)**	-0.004 (-0.043, 0.035)	0.844	0.017 (-0.015, 0.049)	0.305	-0.004 (-0.012, 0.004)	0.307
**Cleaning duration (mins per week)**	-0.0003 (-0.001, 0.0002)	0.295	-0.00005 (-0.0005, 0.0004)	0.824	0.00005 (-0.0001, 0.0001)	0.365
**PCPs use (number of times using a product per week)**	0.004 (0.001, 0.008)	**0.020**	0.003 (-0.0003, 0.006)	0.079	0.0001 (-0.001, 0.001)	0.72
**Perfume use (times per week)**	-0.006 (-0.038, 0.026)	0.708	-0.012 (-0.039, 0.014)	0.369	-0.002 (-0.008, 0.004)	0.564
**Deodorant use(times per week)**	0.008 (-0.025, 0.040)	0.634	0.006 (-0.022, 0.035)	0.672	0.002 (-0.004, 0.008)	0.538
**Cosmetics use (times per week)**	0.007 (-0.003, 0.016)	0.164	-0.004 (-0.012, 0.004)	0.344	0.002 (-0.0002, 0.003)	0.092

All models in the sensitivity analysis have been adjusted for: log-creatinine, study site, disease status, age and BMI

Abbreviations: BPA: bisphenol A, BPF: bisphenol F; ClxBPA: sum of 3-chlorobisphenol A (ClBPA), 3,5-dichlorobisphenol A (3,5-Cl2BPA) and 3,3-dichlorobispheno A (3,3’-Cl2BPA); PCPs: personal care products
