# Supplementary material for: Human Exposures to Bisphenol A, Bisphenol F and Chlorinated Bisphenol A Derivatives and Thyroid Function
Source: PLoS One. 2016 Oct 26;11(10):e0155237. doi: 10.1371/journal.pone.0155237 (PMC5082639; doi:10.1371/journal.pone.0155237)
Supplement: S6 Table — Odds ratios of the univariate logistic regression with participants’ characteristics as predictors (A. models have only each characteristic as predictor; B. models have been adjusted for the study site). (PDF) [file pone.0155237.s006.pdf]

Table S6. Odds ratios of univariate logistic regression with participants' characteristics as predictors (A. models with each characteristic as predictor; B. models have been adjusted for the study site).

**A.**							
**Demographics**	OR	**95%** CI	p-value	**Questionnaire-derived variables**	OR	**95%** CI	p-value
**Age (y)**	1.03	1.01 – 1.06	0.003	**Daily consumption of water from 20L bottles (glasses; 1 glass=250mL)**	0.86	0.72 – 1.00	0.06
**BMI (kg/m2)**	1.1	1.04 – 1.16	0.001	**Frequency of use of the microwave oven (times per week)**	1	0.87 – 1.16	0.964
**Smoking status [never]**				**Consumption of canned food (portion per week)**	0.84	0.73 – 0.95	0.013
**Currently**	1.07	0.51 – 2.21	0.863	**Cleaning duration (mins per week)**	1	1.00 – 1.00	0.327
**Past**	1.6	0.55 – 4.97	0.394	**Frequency of use of PCPs (number of times using a product per week)**	0.99	0.98 – 1.00	0.09
**Alcohol consumption [Rarely/never]**				**Frequency of perfume use (times per week)**	0.87	0.80 – 0.95	0.002
**Weekend**	0.78	0.33 – 1.80	0.557	**Frequency of use of deodorant (times per week)**	0.95	0.87 – 1.03	0.203
**Often**	0.74	0.14 – 3.46	0.701	**Frequency of use of cosmetics (times of using the products per week)**	0.96	0.94 – 0.98	0.001
**Marital status [single]**							
**Married**	3.74	1.49 – 10.72	0.008				
**Divorced**	1.41	0.30 – 6.26	0.654				
**Widow**	4.43	1.05 – 20.69	0.047				

**Other**	6707569.93	0.00 – NA	0.986
**Education [elementary]**			
**Secondary**	0.35	0.13 – 0.89	0.033
**University**	0.23	0.08 – 0.56	0.002
**Other**	1919270.95	0.00 – NA	0.989
**TSH (mIU/L)**	0.57	0.36 – 0.88	**0.014**
**FT4 (pmol/L)**	1.28	0.21 – 7.98	0.79
**Spot iodine (µg/L)**	0.82	0.62 – 1.06	0.129

B.

**Demographics**	**OR**	**95% CI**	**p-value**	**Questionnaire-derived variables**	**OR**	**95% CI**	**p-value**
**Age (y)**	1.04	1.02 – 1.06	0.001	**Daily consumption of water from 20L bottles (glasses; 1 glass=250mL)**	0.87	0.73 – 1.01	0.086
**Study site [Romania]**	1.61	0.91 – 2.87	0.10	**Study site [Romania]**	1.21	0.69 – 2.13	0.515
**BMI (kg/m2)**	1.1	1.04 – 1.16	0.001	**Frequency of use of the microwave oven (times per week)**	1.01	0.87 – 1.17	0.882
**Study site [Romania]**	1.22	0.69 – 2.17	0.49	**Study site [Romania]**	1.39	0.80 – 2.43	0.248
**Smoking status [never]**				**Consumption of canned food (portion per week)**	0.85	0.73 – 0.96	0.018

**Currently**	1.03	0.49 – 2.14	0.942	**Study site [Romania]**	1.14	0.62 – 2.09	0.679
**Past**	1.67	0.57 – 5.21	0.356				
**Study site [Romania]**	1.34	0.77 – 2.35	0.31	**Cleaning duration (mins per week)**	1	1.00 – 1.00	0.249
				**Study site [Romania]**	1.31	0.71 – 2.43	0.396
**Alcohol consumption [Rarely/never]**							
**Weekend**	0.82	0.34 – 1.93	0.652	**Frequency of use of PCPs (number of times using a product per week)**	0.99	0.98 – 1.00	0.257
**Often**	0.82	0.15 – 3.90	0.8	**Study site [Romania]**	1.26	0.60 – 2.65	0.544
**Study site [Romania]**	1.24	0.70 – 2.20	0.46				
				**Frequency of perfume use (times per week)**	0.87	0.80 – 0.95	0.001
**Marital status [single]**				**Study site [Romania]**	1.55	0.87 – 2.76	0.137
**Married**	3.92	1.55 – 11.31	0.006				
**Divorced**	1.38	0.29 – 6.18	0.672	**Frequency of use of deodorant (times per week)**	0.94	0.87 – 1.02	0.169
**Widow**	4.2	0.99 – 19.71	0.057	**Study site [Romania]**	1.56	0.89 – 2.78	0.124
**Other**	5665074.6	0.00 – NA	0.986				
**Study site [Romania]**	1.4	0.79 – 2.52	0.25				
				**Frequency of use of cosmetics (times of using the products per week)**	0.96	0.94 – 0.98	0.001
**Education [elementary]**				**Study site [Romania]**	1.36	0.76 – 2.44	0.297

**Secondary**	0.35	0.12 – 0.88	0.032
**University**	0.23	0.08 – 0.56	0.002
**Other**	1692079.85	0.00 – NA	0.989
**Study site [Romania]**	1.24	0.70 – 2.21	0.46
**TSH (mIU/L)**	0.55	0.34 – 0.86	0.01
**Study site [Romania]**	1.49	0.85 – 2.63	0.166
**FT4 (pmol/L)**	0.78	0.10 – 5.70	0.802
**Study site [Romania]**	1.46	0.80 – 2.67	0.219
**Spot iodine (µg/L)**	1.41	0.81 – 2.48	0.231
**Study site [Romania]**	0.8	0.61 – 1.04	0.097

*Log-transformed (natural logarithm) concentrations of TSH, fT4 and iodine levels were used the univariate models
