# Supplementary material for: Human Exposures to Bisphenol A, Bisphenol F and Chlorinated Bisphenol A Derivatives and Thyroid Function
Source: PLoS One. 2016 Oct 26;11(10):e0155237. doi: 10.1371/journal.pone.0155237 (PMC5082639; doi:10.1371/journal.pone.0155237)
Supplement: S7 Table — (PDF) [file pone.0155237.s007.pdf]

Table S7. Odds ratios of the urinary levels of BPF, BPA and ClxBPA from logistic regression adjusted for the study site.

	OR	95% CI	p-value
**BPA (ng/L)***	0.56	0.40 – 0.77	**<0.001**
**Study site [Romania]**	2.28	1.19 – 4.49	**0.015**
**BPF (ng/L)***	0.96	0.74 – 1.25	0.765
**Study site [Romania]**	1.27	0.73 – 2.22	0.406
**ClxBPA (ng/L)***	0.65	0.16 – 2.44	0.53
**Study site [Romania]**	1.34	0.75 – 2.41	0.32

Note: All concentrations are used log-transformed in the models
*models have been adjusted for creatinine (g/L)
