# Supplementary material for: Human Exposures to Bisphenol A, Bisphenol F and Chlorinated Bisphenol A Derivatives and Thyroid Function
Source: PLoS One. 2016 Oct 26;11(10):e0155237. doi: 10.1371/journal.pone.0155237 (PMC5082639; doi:10.1371/journal.pone.0155237)
Supplement: S8 Table — (PDF) [file pone.0155237.s008.pdf]

Table S8. Sensitivity analysis results of the multivariate logistic regression models.

	A1			A2		
	**OR**	**95% CI**	**p-value**	**OR**	**95% CI**	**p-value**
**BPA (ng/L)**	0.88	0.58 – 1.33	0.553	0.73	0.46 – 1.14	0.166
**BPF (ng/L)**	1.39	0.82 – 2.38	0.222	1.42	0.84 – 2.44	0.2
**ClxBPA (ng/L)**	0.46	0.05 – 3.95	0.481	0.24	0.02 – 2.52	0.242
	**B1**			**B2**		
	**OR**	**95% CI**	**p-value**	**OR**	**95% CI**	**p-value**
**BPA (ng/L)**	0.92	0.60 – 1.40	0.69	0.76	0.48 – 1.18	0.223
**BPF (ng/L)**	1.41	0.84 – 2.43	0.203	1.44	0.85 – 2.50	0.179
**ClxBPA (ng/L)**	0.59	0.06 – 5.26	0.635	0.29	0.02 – 3.07	0.308

Note: All concentrations are log-transformed and all models have been adjusted for log-transformed creatinine

Model details:

(A1) Adjusted for: age, BMI, TSH and FT4

(A2) Adjusted for: study site, age, BMI, TSH and FT4

(B1) Adjusted for: age, BMI, TSH, FT4 and spot iodine (µg/L)

(B2) Adjusted for: study site, age, BMI, TSH, FT4 and spot iodine (µg/L)

Abbreviations: BPA: bisphenol A, BPF: bisphenol F; ClxBPA: sum of 3-chlorobisphenol A (ClBPA), 3,5-dichlorobisphenol A (3,5-Cl2BPA) and 3,3’-dichlorobispheno A (3,3’-Cl2BPA); OR: odds ratio; CI: confidence intervals
