# Supplementary material for: Human Exposures to Bisphenol A, Bisphenol F and Chlorinated Bisphenol A Derivatives and Thyroid Function
Source: PLoS One. 2016 Oct 26;11(10):e0155237. doi: 10.1371/journal.pone.0155237 (PMC5082639; doi:10.1371/journal.pone.0155237)
Supplement: S9 Table — (PDF) [file pone.0155237.s009.pdf]

Table S9. Test for trend in multivariate models where the outcome is log-transformed ft4, adjusted for age, BMI, study site and disease status (creatinine adjustment has been included in all models) for the whole study population.

	**Complete study population**	
	**(A)**<br>**β coefficient (95% CI)**	***p*-value**
**BPA (ng/L)**	0.023 (-0.001, 0.047)	**0.061**
**BPF (ng/L)**	-0.011 (-0.046, 0.024)	0.530
**ClxBPA (ng/L)**	0.058 (-0.068, 0.184)	0.368
	**(B)**<br>**β coefficient (95% CI)**	***p*-value**
**BPA (ng/L)**	0.025 (0.001, 0.049)	**0.046**
**BPF (ng/L)**	-0.006 (-0.042, 0.029)	0.723
**ClxBPA (ng/L)**	0.063 (-0.065, 0.191)	0.338
	**(C)**<br>**β coefficient (95% CI)**	***p*-value**
**BPA (ng/L)**	-0.004 (-0.029, 0.021)	**0.737**
**BPF (ng/L)**	0.006 (-0.027, 0.039)	0.732
**ClxBPA (ng/L)**	**-0.087 (-0.214, 0.041)**	0.184
	**(D)**<br>**β coefficient (95% CI)**	***p*-value**
**BPA (ng/L)**	-0.005 (-0.030, 0.020)	**0.707**
**BPF (ng/L)**	0.006 (-0.027, 0.039)	0.722
**ClxBPA (ng/L)**	-0.087 (-0.215, 0.040)	0.183

Model details:
(A) Adjusted for: log creatinine
(B) Adjusted for: age, BMI and creatinine (g/L, log-transformed)
(C) Adjusted for: age, BMI, study site[Romania] and creatinine
(D) Adjusted for: age, BMI, study site[Romania], disease status[case] (and creatinine)
Abbreviations: BPA: bisphenol A, BPF: bisphenol F; ClxBPA: sum of 3-chlorobisphenol A (ClBPA), 3,5-dichlorobisphenol A (3,5-Cl_2BPA) and 3,3’-dichlorobispheno A (3,3’-Cl_2BPA); CI: confidence intervals
