# Supplementary material for: Human Exposures to Bisphenol A, Bisphenol F and Chlorinated Bisphenol A Derivatives and Thyroid Function
Source: PLoS One. 2016 Oct 26;11(10):e0155237. doi: 10.1371/journal.pone.0155237 (PMC5082639; doi:10.1371/journal.pone.0155237)
Supplement: S10 Table — (PDF) [file pone.0155237.s010.pdf]

Table S10. Linear regression analysis of the exposure habits as determinants of spot urinary BPA levels (creatinine unadjusted, log-transformed) for the cases and controls, separately.

	BPA (ng/L)	
**Cases**		
	**β coefficient (95% CI)**	***p*-value**
**20L Bottled water use (glasses per day; 1 glass=250mL)**	0.143 (0.026, 0.261)	**0.019**
**Microwave oven use (times per week)**	-0.004 (-0.101, 0.092)	0.928
**Canned food consumption (portion per week)**	-0.026 (-0.100, 0.047)	0.485
**Cleaning duration (mins per week)**	-0.001 (-0.002, -0.0002)	0.017
**PCPs use (number of times using a product per week)**	0.008 (0.003, 0.013)	**0.003**
**Perfume use (times per week)**	0.011 (-0.033, 0.055)	0.638
**Deodorant use (times per week)**	0.015 (-0.028, 0.059)	0.490
**Cosmetics use (times per week)**	0.008 (-0.007, 0.024)	0.292
**Controls**		
	**β coefficient (95% CI)**	***p*-value**
**20L Bottled water use (glasses per day; 1 glass=250mL)**	0.071 (-0.023, 0.164)	**0.142**
**Microwave oven use (times per week)**	-0.006 (-0.106, 0.094)	0.903
**Canned food consumption (portion per week)**	0.0005 (-0.065, 0.066)	0.989
**Cleaning duration (mins per week)**	-0.00002 (-0.001, 0.001)	0.965
**PCPs use (number of times using a product per week)**	-0.003 (-0.011, 0.005)	**0.529**
**Perfume use (times per week)**	-0.076 (-0.139, -0.013)	0.022
**Deodorant use (times per week)**	-0.008 (-0.080, 0.065)	0.838
**Cosmetics use (times per week)**	-0.001 (-0.016, 0.015)	0.920

*The models have been adjusted for: log-creatinine, study site, disease status, age and BMI
Abbreviations: BPA: bisphenol A, BPF: bisphenol F; ClxBPA: sum of 3-chlorobisphenol A (ClBPA), 3,5-dichlorobisphenol A (3,5-Cl2BPA) and 3,3’-dichlorobispheno A (3,3’-Cl2BPA); PCPs: personal care products, CI: confidence intervals
