# Supplementary material for: Human Exposures to Bisphenol A, Bisphenol F and Chlorinated Bisphenol A Derivatives and Thyroid Function
Source: PLoS One. 2016 Oct 26;11(10):e0155237. doi: 10.1371/journal.pone.0155237 (PMC5082639; doi:10.1371/journal.pone.0155237)
Supplement: S19 Table — (HTML) [file pone.0155237.s019.html]

Final THYROCHEM report - REPORT - tables


# Final THYROCHEM report - REPORT - tables

#### *Xanthi D. Andrianou - Water and Health Lab*

- 1 Preliminaries
  - 1.1 Packages used
  - 1.2 Raw data files
  - 1.3 Variable lists
  - 1.4 Functions
  - 1.5 Questionnaire variable recoding
- 2 Cyprus
  - 2.1 Table S4
- 3 Romania
- 4 Working on the both countries (pooled population)
  - 4.1 Descriptives and comparisons - demographics, hormones, iodine and urinary levels of BPA, BPF, ClxBPA
    - 4.1.1 Table 1, Table S1, Table S3
  - 4.2 Logistic regressions
    - 4.2.1 Table S6, Table S7
    - 4.2.2 Table 3
    - 4.2.3 Table S8
- 5 Determinants of exposure by compound in the whole study population
  - 5.0.1 Table 2
  - 5.1 Table S5
  - 5.2 Table S10 (additional table)
- 6 BPA and TSH - trends
  - 6.1 Table 4
  - 6.2 Additional trend analysis for fT4
    - 6.2.1 Table S9
  - 6.3 Plots between BPA, TSH and iodine

This report is based on the full analysis of the THYROCHEM dataset including both the Cypriot and Romanian data.

# 1 Preliminaries

## 1.1 Packages used

The R packages used in the analysis are the following: “ggplot2”,“dplyr”,“data.table”,“Hmisc”,“stargazer”,“psych”,“plyr”,“tableone”, “sjPlot”,“corrplot”,“ICC”, “irr”,“coefplot”,“car”,“knitr” Complete information on the versions and the exact citations are provided in the end of the report. —

## 1.2 Raw data files

The raw data files used for the analysis are the following:

- Cyprus
- questionnaire - CY\_20150714\_Questionnaire\_DF.csv
- BPA results - CY\_BPA\_spot\_rawdata\_DF.csv and CY\_BPA\_morn\_rawdata\_DF.csv
- Iodine results (incl. creatinine) - CY\_creat\_iodine\_DF.csv
- Romania
- questionnaire - RO\_Questionnaire\_150711\_DF.csv
- BPA results - RO\_BPA\_rawdata.csv
- Iodine results - RO\_creat\_iodine\_DF.csv

## 1.3 Variable lists

The variables were all taken from the raw data. Questionnaire variables were summarized to be used in the summary analysis of the exposures. Variables that were not directly measured such as BMI or the creatinine-adjustment of the urinary concentrations were created during the analysis. Specific variable lists were created and used in the present script for efficiency.

List of the different combinations of the variables used in the analysis:

- quest\_vars - all the variables extracted from the questionnaire and used in the analysis
- quest\_analysis\_vars - questionnaire variables used in the final analysis
- demo\_vars - demographics and anthropometric characteristics
- expo\_vars - summary of the “questionniare exposures”
- horm\_vars\_iod\_sp - hormones and spot iodine (ng/L) (all continuous)
- horm\_vars\_cat - categorical variables of the hormone levels and the antibodies
- *Note: the categorization of the hormone leves was done separately for Cyprus and Romania since the normal levels differ in the two countries.*
- urine\_levs\_vars - ng/L urine levels both spot and morning (raw, ng/L and ng/g)
- urine\_levs\_vars\_av - urine levels average levels {derived with the function (spot+morn)/2}
- ln\_vars\_spot - ln-transformed ng/L and ng/g - spot
- ln\_vars\_morn - ln-transformed ng/L and ng/g - morning
- ln\_vars\_av - ln-transformed average ng/L and ng/g - (ln of the average not average of the ln\_spot and ln\_morn)
- quant\_vars - quantiles of the BPF, BPA and ClxBPA (ng/L)

## 1.4 Functions

General functions

- creatine\_adj – creatinine adjustment (ng/L concentrations divided by the creatinine) \_Note: iodine concentrations were already adjusted for creatinine and the units were ug/L and ug/g (unadjusted and adjusted, respectively)
- spot\_morn\_av – average of spot and morning - applied to the ng/L and the ng/g
- gm\_func – geometric mean
- gsd\_func – geometric standard deviation
- break\_quant – breaking the quantiles (0,0.25,0.50,0.75,1) - including the lowest bound
- t\_test\_unpaired\_table – table for the results of the unpaired t-test - applied to the ln-transformed ng/L and ng/g concentrations, hormones etc.
- wilcox\_test\_unpaired\_table – table for the results of the unpaired Wilcoxon test - applied to the raw ng/L and ng/g concentrations, hormones etc.

Logistic regression functions

\_Ξotes: The logistic regression fuctions were used separately for each country and the pooled samples.

All the concentration variables were used in the models log-transformed (to the natural logarithm) including the hormones, iodine and creatinine.

- *Univariate regressions*
  - All variables (incl. those recoded from the questionnaires) were included in univariate models
- *Multivariate regressions*
  - LR1: adjusted for age, BMI, tsh and ft4
  - LR2: adjusted for age, BMI, tsh, ft4 and spot iodine

## 1.5 Questionnaire variable recoding

List of the final external exposure measurement variables are:

- Water consumption from 20L PC bottles
- Weekly use of microwave (with plastic container?)
- Weekly consumption of canned food (number of portions)
- Duration of cleaning activities (as proxy to chlorine exposures)
- Weekly use of PCPs (sum of: shampoo, conditioner, body lotion, shower gel, hair dye, hair foam, hair spray, nail polish, lip care products, face cream)
- Weekly use of perfume and deodorant
- Weekly use of perfume and deodorant and weekly use of cosmetics (makeup, lipstick, eyeliner, rouge, mascara, makeup remover).

```
## Using CODE as id variables
## Using CODE as id variables
```

# 2 Cyprus

## 2.1 Table S4

```
## strata: Overall
##                          n miss p.miss  mean     sd median    p25  p75   min     max skew
## BPF_ngPERlt_spot       122    1   0.82  3181  27304    485  365.0  680 134.0  300728 11.0
## BPA_ngPERlt_spot       122    1   0.82  2495   3295   1508  789.0 2806 273.0   24606  4.2
## ClBPA_ngPERlt_spot     122    1   0.82    74     13     71   66.0   78  56.0     148  2.5
## Cl35_2BPA_ngPERlt_spot 122    1   0.82    73     18     67   65.0   71  62.0     144  3.0
## Cl33_2BPA_ngPERlt_spot 122    1   0.82    17     16     15    5.0   15   5.0      80  1.8
## ClxBPA_ngPERlt_spot    122    1   0.82   164     39    152  141.0  168 129.0     357  2.5
## BPF_ngPERg_spot        122    1   0.82  2130  11351    646  397.8 1327 106.1  125199 10.8
## BPA_ngPERg_spot        122    1   0.82  3380   3626   2101 1184.3 4220 214.5   20238  2.4
## ClBPA_ngPERg_spot      122    1   0.82   149    160     89   58.7  159  23.5     810  2.5
## Cl35_2BPA_ngPERg_spot  122    1   0.82   152    169     87   51.7  183  17.4     960  2.6
## Cl33_2BPA_ngPERg_spot  122    1   0.82    34     48     17    7.2   39   2.1     323  3.3
## ClxBPA_ngPERg_spot     122    1   0.82   335    365    199  120.2  376  44.8    1827  2.5
## BPF_ngPERlt_morn       122    8   6.56  1404   5833    550  402.2  878 151.0   61870 10.1
## BPA_ngPERlt_morn       122    8   6.56 11216  62924   2208 1457.5 4632 505.0  654989  9.8
## ClBPA_ngPERlt_morn     122    8   6.56    83     47     73   67.0   83  56.0     512  7.3
## Cl35_2BPA_ngPERlt_morn 122    8   6.56    83     75     68   65.0   76  62.0     848  9.4
## Cl33_2BPA_ngPERlt_morn 122    8   6.56    28     23     15   15.0   41   5.0     100  1.2
## ClxBPA_ngPERlt_morn    122    8   6.56   194    104    164  150.0  203 124.0    1045  5.8
## BPF_ngPERg_morn        122   10   8.20  2236   6048    815  445.0 1219 116.6   48222  6.0
## BPA_ngPERg_morn        122   10   8.20 26119 172516   3545 1595.6 6885 396.8 1809362 10.1
## ClBPA_ngPERg_morn      122   10   8.20   216    681     97   58.5  159  35.1    7111  9.6
## Cl35_2BPA_ngPERg_morn  122   10   8.20   211    660     96   51.4  172  33.0    6889  9.6
## Cl33_2BPA_ngPERg_morn  122   10   8.20    88    430     27   13.3   56   1.8    4556 10.3
## ClxBPA_ngPERg_morn     122   10   8.20   514   1760    228  127.3  403  76.5   18556  9.9
##                          kurt   gm gsd
## BPF_ngPERlt_spot       120.47  548 2.3
## BPA_ngPERlt_spot        22.14 1575 2.5
## ClBPA_ngPERlt_spot       9.53   73 1.2
## Cl35_2BPA_ngPERlt_spot   8.58   71 1.2
## Cl33_2BPA_ngPERlt_spot   3.07   12 2.2
## ClxBPA_ngPERlt_spot      6.91  160 1.2
## BPF_ngPERg_spot        117.99  775 2.6
## BPA_ngPERg_spot          6.12 2229 2.5
## ClBPA_ngPERg_spot        6.63  103 2.2
## Cl35_2BPA_ngPERg_spot    7.27  101 2.4
## Cl33_2BPA_ngPERg_spot   13.06   18 3.1
## ClxBPA_ngPERg_spot       6.44  227 2.3
## BPF_ngPERlt_morn       104.71  637 2.3
## BPA_ngPERlt_morn        99.47 2727 3.0
## ClBPA_ngPERlt_morn      63.54   78 1.3
## Cl35_2BPA_ngPERlt_morn  95.77   75 1.4
## Cl33_2BPA_ngPERlt_morn   0.87   20 2.3
## ClxBPA_ngPERlt_morn     42.71  182 1.4
## BPF_ngPERg_morn         39.36  897 3.0
## BPA_ngPERg_morn        105.50 3923 3.5
## ClBPA_ngPERg_morn       96.96  111 2.4
## Cl35_2BPA_ngPERg_morn   96.98  107 2.4
## Cl33_2BPA_ngPERg_morn  107.84   29 3.2
## ClxBPA_ngPERg_morn     101.95  259 2.4
```

Table: CY - Summary of the urinary levels (all participants)

```
## Note: All the t-tests have been applied to the ln-transformed concentrations
```

Paired t-test p-values and ICC - spot and morning samples - CY (pooled population) - Table S4

|  | method | p.value | ICC | LowerCI | UpperCI |
| --- | --- | --- | --- | --- | --- |
| BPF\_ngPERlt | Paired t-test | 0.0634916683119793 | 0.458845971316997 | 0.313423305588782 | 0.604268637045211 |
| BPA\_ngPERlt | Paired t-test | 1.46954396636618e-06 | 0.155802749962952 | -0.0261253660406351 | 0.33773086596654 |
| ClBPA\_ngPERlt | Paired t-test | 0.00252995275638623 | 0.308083752492721 | 0.140491212944793 | 0.475676292040649 |
| Cl35\_2BPA\_ngPERlt | Paired t-test | 0.159524255332564 | -0.0667064538405715 | -0.255153567560228 | 0.121740659879085 |
| Cl33\_2BPA\_ngPERlt | Paired t-test | 1.10646310277686e-05 | 0.104054801265897 | -0.0809110631645849 | 0.289020665696379 |
| ClxBPA\_ngPERlt | Paired t-test | 0.000207443636623444 | 0.0940296772627003 | -0.0914175838605487 | 0.279476938385949 |
| BPF\_ngPERg | Paired t-test | 0.27995859920388 | 0.208007959709809 | 0.0282177416208153 | 0.387798177798803 |
| BPA\_ngPERg | Paired t-test | 5.08372802699384e-06 | 0.213340005288653 | 0.0340273169482114 | 0.392652693629095 |
| ClBPA\_ngPERg | Paired t-test | 0.531221435937399 | 0.308188173404376 | 0.13901669803282 | 0.477359648775931 |
| Cl35\_2BPA\_ngPERg | Paired t-test | 0.743882297631523 | 0.3370977799242 | 0.171638782267132 | 0.502556777581268 |
| Cl33\_2BPA\_ngPERg | Paired t-test | 0.00191501038735322 | 0.202600424358262 | 0.0223359602335225 | 0.382864888483001 |
| ClxBPA\_ngPERg | Paired t-test | 0.273129603713081 | 0.316536848613297 | 0.148407617731461 | 0.484666079495132 |

# 3 Romania

# 4 Working on the both countries (pooled population)

## 4.1 Descriptives and comparisons - demographics, hormones, iodine and urinary levels of BPA, BPF, ClxBPA

### 4.1.1 Table 1, Table S1, Table S3

Demographics (all study population - by status and in the two countries - Table 1)

|  | Overall | CA | CO | p | test | Overall | CA | CO | p | test | Overall | CA | CO | p | test |
| --- | --- | --- | --- | --- | --- | --- | --- | --- | --- | --- | --- | --- | --- | --- | --- |
| n | 212 | 106 | 106 |  |  | 122 | 57 | 65 |  |  | 90 | 49 | 41 |  |  |
| age (mean (sd)) | 49.21 (12.67) | 51.87 (11.76) | 46.56 (13.04) | 0.002 |  | 50.88 (12.27) | 52.75 (12.39) | 49.23 (12.02) | 0.114 |  | 46.96 (12.92) | 50.84 (11.02) | 42.32 (13.62) | 0.001 |  |
| weight (mean (sd)) | 69.55 (14.25) | 72.34 (15.59) | 66.68 (12.14) | 0.004 |  | 67.04 (12.16) | 68.04 (13.00) | 66.14 (11.38) | 0.397 |  | 72.93 (16.11) | 77.35 (16.94) | 67.52 (13.34) | 0.004 |  |
| heightM (mean (sd)) | 1.64 (0.06) | 1.63 (0.06) | 1.64 (0.06) | 0.102 |  | 1.63 (0.06) | 1.62 (0.06) | 1.63 (0.06) | 0.691 |  | 1.65 (0.06) | 1.63 (0.06) | 1.66 (0.06) | 0.016 |  |
| BMI (mean (sd)) | 26.08 (5.50) | 27.39 (6.10) | 24.75 (4.48) | 0.001 |  | 25.39 (4.56) | 25.85 (4.91) | 24.98 (4.22) | 0.303 |  | 27.00 (6.46) | 29.11 (6.85) | 24.41 (4.88) | <0.001 |  |
| BMI\_cat (%) |  |  |  | 0.035 | exact |  |  |  | 0.667 | exact |  |  |  | 0.021 | exact |
| Underweight | 5 ( 2.4) | 1 ( 1.0) | 4 ( 3.9) |  |  | 2 ( 1.7) | 0 ( 0.0) | 2 ( 3.2) |  |  | 3 ( 3.4) | 1 ( 2.0) | 2 ( 5.0) |  |  |
| Normal | 91 (44.2) | 39 (37.5) | 52 (51.0) |  |  | 56 (47.9) | 25 (45.5) | 31 (50.0) |  |  | 35 (39.3) | 14 (28.6) | 21 (52.5) |  |  |
| Overweight | 61 (29.6) | 32 (30.8) | 29 (28.4) |  |  | 39 (33.3) | 20 (36.4) | 19 (30.6) |  |  | 22 (24.7) | 12 (24.5) | 10 (25.0) |  |  |
| Obese | 49 (23.8) | 32 (30.8) | 17 (16.7) |  |  | 20 (17.1) | 10 (18.2) | 10 (16.1) |  |  | 29 (32.6) | 22 (44.9) | 7 (17.5) |  |  |
| smokst (%) |  |  |  | 0.691 |  |  |  |  | 0.858 |  |  |  |  | 0.677 |  |
| Never | 157 (75.5) | 76 (73.8) | 81 (77.1) |  |  | 94 (77.0) | 43 (75.4) | 51 (78.5) |  |  | 63 (73.3) | 33 (71.7) | 30 (75.0) |  |  |
| Currently | 36 (17.3) | 18 (17.5) | 18 (17.1) |  |  | 17 (13.9) | 8 (14.0) | 9 (13.8) |  |  | 19 (22.1) | 10 (21.7) | 9 (22.5) |  |  |
| Past | 15 ( 7.2) | 9 ( 8.7) | 6 ( 5.7) |  |  | 11 ( 9.0) | 6 (10.5) | 5 ( 7.7) |  |  | 4 ( 4.7) | 3 ( 6.5) | 1 ( 2.5) |  |  |
| alccons (%) |  |  |  | 0.817 | exact |  |  |  | 0.524 | exact |  |  |  | 0.364 | exact |
| Never/Rarely | 175 (84.5) | 88 (86.3) | 87 (82.9) |  |  | 95 (77.9) | 47 (82.5) | 48 (73.8) |  |  | 80 (94.1) | 41 (91.1) | 39 (97.5) |  |  |
| Weekend | 25 (12.1) | 11 (10.8) | 14 (13.3) |  |  | 20 (16.4) | 7 (12.3) | 13 (20.0) |  |  | 5 ( 5.9) | 4 ( 8.9) | 1 ( 2.5) |  |  |
| Often | 7 ( 3.4) | 3 ( 2.9) | 4 ( 3.8) |  |  | 7 ( 5.7) | 3 ( 5.3) | 4 ( 6.2) |  |  | 0 ( 0.0) | 0 ( 0.0) | 0 ( 0.0) |  |  |
| marstat (%) |  |  |  | 0.017 | exact |  |  |  | 0.177 | exact |  |  |  | 0.033 | exact |
| Single | 25 (12.0) | 6 ( 5.8) | 19 (18.1) |  |  | 13 (10.7) | 4 ( 7.0) | 9 (13.8) |  |  | 12 (14.0) | 2 ( 4.3) | 10 (25.0) |  |  |
| Married | 157 (75.5) | 85 (82.5) | 72 (68.6) |  |  | 99 (81.1) | 49 (86.0) | 50 (76.9) |  |  | 58 (67.4) | 36 (78.3) | 22 (55.0) |  |  |
| Divorced | 13 ( 6.2) | 4 ( 3.9) | 9 ( 8.6) |  |  | 6 ( 4.9) | 1 ( 1.8) | 5 ( 7.7) |  |  | 7 ( 8.1) | 3 ( 6.5) | 4 (10.0) |  |  |
| Widow | 12 ( 5.8) | 7 ( 6.8) | 5 ( 4.8) |  |  | 4 ( 3.3) | 3 ( 5.3) | 1 ( 1.5) |  |  | 8 ( 9.3) | 4 ( 8.7) | 4 (10.0) |  |  |
| Other | 1 ( 0.5) | 1 ( 1.0) | 0 ( 0.0) |  |  | 0 ( 0.0) | 0 ( 0.0) | 0 ( 0.0) |  |  | 1 ( 1.2) | 1 ( 2.2) | 0 ( 0.0) |  |  |
| edu (%) |  |  |  | 0.003 | exact |  |  |  | 0.134 | exact |  |  |  | 0.001 | exact |
| Primary | 28 (13.5) | 21 (20.4) | 7 ( 6.7) |  |  | 16 (13.1) | 11 (19.3) | 5 ( 7.7) |  |  | 12 (14.1) | 10 (21.7) | 2 ( 5.1) |  |  |
| Secondary | 78 (37.7) | 40 (38.8) | 38 (36.5) |  |  | 43 (35.2) | 17 (29.8) | 26 (40.0) |  |  | 35 (41.2) | 23 (50.0) | 12 (30.8) |  |  |
| University | 99 (47.8) | 40 (38.8) | 59 (56.7) |  |  | 63 (51.6) | 29 (50.9) | 34 (52.3) |  |  | 36 (42.4) | 11 (23.9) | 25 (64.1) |  |  |
| Other | 2 ( 1.0) | 2 ( 1.9) | 0 ( 0.0) |  |  | 0 ( 0.0) | 0 ( 0.0) | 0 ( 0.0) |  |  | 2 ( 2.4) | 2 ( 4.3) | 0 ( 0.0) |  |  |

Hormone and iodine (all study population - by status and in the two countries) - Table 1

|  | Overall | CA | CO | p | test | Overall | CA | CO | p | test | Overall | CA | CO | p | test |
| --- | --- | --- | --- | --- | --- | --- | --- | --- | --- | --- | --- | --- | --- | --- | --- |
| n | 212 | 106 | 106 |  |  | 122 | 57 | 65 |  |  | 90 | 49 | 41 |  |  |
| tsh (median [IQR]) | 1.58 [1.05, 2.19] | 1.42 [0.90, 1.97] | 1.68 [1.18, 2.49] | 0.016 | nonnorm | 1.51 [1.06, 2.00] | 1.40 [0.90, 1.98] | 1.53 [1.08, 2.00] | 0.559 | nonnorm | 1.68 [1.05, 2.55] | 1.44 [0.83, 1.85] | 2.20 [1.28, 2.94] | 0.003 | nonnorm |
| ft4 (median [IQR]) | 13.40 [12.11, 14.90] | 13.40 [12.40, 14.90] | 13.43 [12.06, 14.90] | 0.748 | nonnorm | 12.70 [11.80, 14.00] | 12.90 [11.90, 13.95] | 12.60 [11.80, 14.00] | 0.606 | nonnorm | 14.71 [13.10, 16.10] | 14.60 [12.90, 15.70] | 14.80 [13.50, 16.10] | 0.598 | nonnorm |
| iod\_ugPERlt\_spot (median [IQR]) | 107.80 [48.90, 185.62] | 105.65 [44.47, 157.93] | 110.60 [53.76, 190.03] | 0.320 | nonnorm | 94.30 [40.40, 190.00] | 82.00 [26.80, 165.50] | 103.35 [48.42, 190.03] | 0.254 | nonnorm | 118.40 [60.53, 182.80] | 115.80 [64.47, 153.00] | 127.75 [58.86, 188.93] | 0.583 | nonnorm |
| iod\_ugPERg\_spot (median [IQR]) | 121.22 [71.94, 180.16] | 110.30 [70.89, 160.07] | 134.50 [77.15, 187.65] | 0.086 | nonnorm | 114.30 [67.40, 185.20] | 109.30 [59.40, 178.30] | 134.50 [69.60, 185.80] | 0.303 | nonnorm | 125.72 [84.03, 174.82] | 114.46 [84.58, 147.03] | 134.33 [83.38, 189.68] | 0.119 | nonnorm |

BPA, BPF, ClxBPA (all study population - by status and in the two countries) - Table 1

|  | Overall | CA | CO | p | test | Overall | CA | CO | p | test | Overall | CA | CO | p | test |
| --- | --- | --- | --- | --- | --- | --- | --- | --- | --- | --- | --- | --- | --- | --- | --- |
| n | 212 | 106 | 106 |  |  | 122 | 57 | 65 |  |  | 90 | 49 | 41 |  |  |
| BPF\_ngPERlt\_spot (median [IQR]) | 464.50 [320.25, 719.50] | 463.00 [338.00, 679.50] | 480.00 [287.25, 788.75] | 0.703 | nonnorm | 485.00 [365.00, 680.00] | 457.00 [378.00, 634.00] | 510.00 [359.75, 761.00] | 0.540 | nonnorm | 416.00 [219.00, 822.00] | 492.00 [256.00, 822.00] | 371.00 [158.50, 1034.50] | 0.320 | nonnorm |
| BPA\_ngPERlt\_spot (median [IQR]) | 2258.00 [1099.75, 4610.75] | 1751.00 [1117.25, 3458.25] | 2710.50 [1081.00, 5912.00] | 0.016 | nonnorm | 1508.00 [789.00, 2806.00] | 1394.00 [757.00, 2189.00] | 1831.50 [792.00, 3003.50] | 0.333 | nonnorm | 3778.00 [2134.00, 9050.00] | 2453.00 [1495.00, 5173.00] | 8012.50 [3112.00, 19288.00] | <0.001 | nonnorm |
| ClBPA\_ngPERlt\_spot (median [IQR]) | 73.00 [67.00, 81.75] | 72.00 [66.25, 79.75] | 74.00 [68.00, 83.50] | 0.099 | nonnorm | 71.00 [66.00, 78.00] | 70.00 [66.00, 76.00] | 71.50 [65.75, 80.50] | 0.297 | nonnorm | 76.00 [71.00, 88.00] | 75.00 [69.00, 83.00] | 79.00 [72.75, 94.00] | 0.108 | nonnorm |
| Cl35\_2BPA\_ngPERlt\_spot (median [IQR]) | 68.00 [65.00, 73.00] | 68.00 [65.00, 74.00] | 68.00 [65.00, 71.25] | 0.625 | nonnorm | 67.00 [65.00, 71.00] | 67.00 [65.00, 71.00] | 67.00 [65.00, 71.00] | 0.442 | nonnorm | 69.00 [65.00, 75.00] | 71.00 [66.00, 78.00] | 69.00 [64.00, 74.00] | 0.118 | nonnorm |
| Cl33\_2BPA\_ngPERlt\_spot (median [IQR]) | 15.00 [5.00, 33.75] | 15.00 [5.00, 33.75] | 15.00 [5.00, 33.25] | 0.835 | nonnorm | 15.00 [5.00, 15.00] | 15.00 [5.00, 15.00] | 15.00 [5.00, 15.00] | 0.264 | nonnorm | 15.00 [15.00, 44.00] | 15.00 [15.00, 44.00] | 15.00 [15.00, 43.25] | 0.701 | nonnorm |
| ClxBPA\_ngPERlt\_spot (median [IQR]) | 159.00 [147.00, 186.75] | 158.50 [144.00, 183.75] | 159.00 [147.75, 187.25] | 0.757 | nonnorm | 152.00 [141.00, 168.00] | 150.00 [140.00, 167.00] | 153.00 [143.50, 168.50] | 0.357 | nonnorm | 177.00 [158.00, 201.00] | 178.00 [158.00, 201.00] | 176.50 [160.50, 201.25] | 0.860 | nonnorm |
| BPF\_ngPERg\_spot (median [IQR]) | 586.32 [331.45, 1229.15] | 603.64 [374.95, 1128.38] | 565.39 [314.85, 1419.45] | 0.679 | nonnorm | 645.81 [397.80, 1327.18] | 740.98 [463.54, 1192.43] | 603.14 [348.77, 1419.45] | 0.442 | nonnorm | 499.64 [271.02, 1138.41] | 472.58 [298.39, 997.72] | 535.99 [231.07, 1332.31] | 0.853 | nonnorm |
| BPA\_ngPERg\_spot (median [IQR]) | 2763.28 [1519.81, 5685.52] | 2333.19 [1497.61, 4554.86] | 3860.16 [1530.03, 8317.94] | 0.029 | nonnorm | 2101.40 [1184.35, 4219.70] | 2199.74 [1184.35, 4086.99] | 1996.07 [1209.57, 4244.96] | 0.963 | nonnorm | 4669.68 [2231.12, 10050.39] | 2800.92 [1826.15, 4809.27] | 8536.81 [4454.26, 23687.69] | <0.001 | nonnorm |
| ClBPA\_ngPERg\_spot (median [IQR]) | 86.21 [58.28, 159.04] | 84.57 [58.49, 166.51] | 87.96 [54.06, 158.16] | 0.979 | nonnorm | 89.36 [58.66, 159.20] | 89.36 [64.99, 175.39] | 89.79 [51.62, 156.95] | 0.415 | nonnorm | 83.42 [58.23, 153.36] | 79.40 [54.10, 126.23] | 85.27 [66.28, 161.97] | 0.324 | nonnorm |
| Cl35\_2BPA\_ngPERg\_spot (median [IQR]) | 82.45 [51.25, 167.86] | 81.71 [54.41, 168.86] | 84.84 [46.89, 157.11] | 0.589 | nonnorm | 87.07 [51.67, 182.58] | 87.07 [61.64, 182.58] | 86.54 [44.79, 173.43] | 0.347 | nonnorm | 78.70 [50.15, 147.73] | 78.93 [50.15, 128.21] | 76.76 [50.55, 148.73] | 0.698 | nonnorm |
| Cl33\_2BPA\_ngPERg\_spot (median [IQR]) | 20.23 [8.02, 46.42] | 19.98 [8.71, 38.92] | 20.79 [7.60, 49.59] | 0.793 | nonnorm | 17.32 [7.20, 39.27] | 15.06 [8.03, 28.96] | 20.55 [7.18, 45.19] | 0.578 | nonnorm | 23.81 [9.78, 64.29] | 23.81 [10.30, 62.30] | 22.72 [9.63, 85.00] | 0.986 | nonnorm |
| ClxBPA\_ngPERg\_spot (median [IQR]) | 199.31 [123.65, 391.39] | 200.04 [126.59, 386.55] | 198.35 [117.87, 395.85] | 0.793 | nonnorm | 199.19 [120.17, 375.68] | 199.19 [135.48, 415.73] | 198.35 [112.28, 362.14] | 0.403 | nonnorm | 200.90 [125.18, 393.60] | 200.90 [124.72, 286.37] | 205.57 [133.19, 406.38] | 0.444 | nonnorm |

Determinants of exposure (all study population - by status and in the two countries) - Table S1

|  | Overall | CA | CO | p | test | Overall | CA | CO | p | test | Overall | CA | CO | p | test |
| --- | --- | --- | --- | --- | --- | --- | --- | --- | --- | --- | --- | --- | --- | --- | --- |
| n | 212 | 106 | 106 |  |  | 122 | 57 | 65 |  |  | 90 | 49 | 41 |  |  |
| daily20lglass (median [IQR]) | 0.00 [0.00, 0.00] | 0.00 [0.00, 0.00] | 0.00 [0.00, 0.00] | 0.149 | nonnorm | 0.00 [0.00, 0.91] | 0.00 [0.00, 0.00] | 0.00 [0.00, 2.14] | 0.294 | nonnorm | 0.00 [0.00, 0.00] | 0.00 [0.00, 0.00] | 0.00 [0.00, 0.00] | 0.375 | nonnorm |
| MwaveFrw (median [IQR]) | 0.00 [0.00, 0.00] | 0.00 [0.00, 0.00] | 0.00 [0.00, 0.00] | 0.994 | nonnorm | 0.00 [0.00, 0.00] | 0.00 [0.00, 0.00] | 0.00 [0.00, 0.02] | 0.115 | nonnorm | 0.00 [0.00, 0.00] | 0.00 [0.00, 0.00] | 0.00 [0.00, 0.00] | 0.005 | nonnorm |
| weekPORTIONScanned (median [IQR]) | 1.25 [0.38, 2.75] | 1.00 [0.27, 2.22] | 1.50 [0.53, 3.50] | 0.010 | nonnorm | 1.88 [0.75, 3.01] | 1.77 [0.70, 2.28] | 2.25 [0.87, 3.81] | 0.059 | nonnorm | 0.50 [0.00, 2.00] | 0.38 [0.00, 1.12] | 0.75 [0.12, 2.50] | 0.109 | nonnorm |
| weekMINScleaning (median [IQR]) | 277.50 [161.25, 427.50] | 292.50 [173.12, 495.00] | 262.50 [142.00, 396.12] | 0.219 | nonnorm | 308.62 [177.50, 504.38] | 321.16 [202.81, 524.75] | 302.40 [171.25, 446.25] | 0.512 | nonnorm | 211.25 [115.00, 355.00] | 240.00 [102.50, 399.88] | 174.75 [115.00, 306.25] | 0.242 | nonnorm |
| weekPORTIONSpcps (median [IQR]) | 70.00 [53.75, 96.25] | 65.72 [41.69, 91.69] | 74.38 [58.00, 97.58] | 0.036 | nonnorm | 86.20 [65.86, 103.00] | 81.35 [65.59, 103.12] | 86.76 [67.14, 102.75] | 0.830 | nonnorm | 50.50 [32.50, 65.00] | 41.12 [25.12, 59.00] | 58.00 [51.50, 68.75] | 0.008 | nonnorm |
| perfwc (median [IQR]) | 7.00 [1.00, 7.00] | 5.00 [0.00, 7.00] | 7.00 [3.50, 7.00] | <0.001 | nonnorm | 7.00 [1.50, 7.00] | 5.00 [0.75, 7.00] | 7.00 [2.00, 7.00] | 0.025 | nonnorm | 7.00 [1.00, 7.00] | 4.50 [0.00, 7.00] | 7.00 [5.00, 7.00] | 0.008 | nonnorm |
| weekPORTIONSdeo (median [IQR]) | 7.00 [2.88, 7.00] | 7.00 [0.00, 7.00] | 7.00 [7.00, 7.00] | 0.187 | nonnorm | 7.00 [3.25, 7.00] | 7.00 [1.00, 7.00] | 7.00 [4.25, 7.00] | 0.973 | nonnorm | 7.00 [3.00, 7.00] | 7.00 [0.00, 7.00] | 7.00 [7.00, 7.00] | 0.046 | nonnorm |
| weekPORTIONScosm (median [IQR]) | 7.00 [0.00, 22.06] | 3.88 [0.00, 15.75] | 14.00 [1.00, 28.00] | <0.001 | nonnorm | 7.00 [0.18, 22.25] | 6.62 [0.02, 16.88] | 9.00 [1.00, 22.88] | 0.069 | nonnorm | 7.00 [0.00, 21.12] | 1.00 [0.00, 13.00] | 20.50 [2.00, 28.00] | 0.003 | nonnorm |

Antibodies (all study population) - Table S2

|  | Overall | CA | CO | p | test | CY | RO | p | test |
| --- | --- | --- | --- | --- | --- | --- | --- | --- | --- |
| n | 212 | 106 | 106 |  |  | 122 | 90 |  |  |
| antitg = Positive (%) | 10 ( 7.8) | 3 ( 4.5) | 7 (11.3) | 0.264 |  | 3 ( 5.1) | 7 (10.0) | 0.478 |  |
| antitpo = Positive (%) | 31 (22.3) | 12 (16.2) | 19 (29.2) | 0.102 |  | 9 (14.8) | 22 (28.2) | 0.092 |  |

BPA, BPF, ClxBPA - by country - Table S3

|  | CY | RO | p | test |
| --- | --- | --- | --- | --- |
| n | 122 | 90 |  |  |
| BPF\_ngPERlt\_spot (median [IQR]) | 485.00 [365.00, 680.00] | 416.00 [219.00, 822.00] | 0.091 | nonnorm |
| BPA\_ngPERlt\_spot (median [IQR]) | 1508.00 [789.00, 2806.00] | 3778.00 [2134.00, 9050.00] | <0.001 | nonnorm |
| ClBPA\_ngPERlt\_spot (median [IQR]) | 71.00 [66.00, 78.00] | 76.00 [71.00, 88.00] | <0.001 | nonnorm |
| Cl35\_2BPA\_ngPERlt\_spot (median [IQR]) | 67.00 [65.00, 71.00] | 69.00 [65.00, 75.00] | 0.076 | nonnorm |
| Cl33\_2BPA\_ngPERlt\_spot (median [IQR]) | 15.00 [5.00, 15.00] | 15.00 [15.00, 44.00] | <0.001 | nonnorm |
| ClxBPA\_ngPERlt\_spot (median [IQR]) | 152.00 [141.00, 168.00] | 177.00 [158.00, 201.00] | <0.001 | nonnorm |
| BPF\_ngPERg\_spot (median [IQR]) | 645.81 [397.80, 1327.18] | 499.64 [271.02, 1138.41] | 0.014 | nonnorm |
| BPA\_ngPERg\_spot (median [IQR]) | 2101.40 [1184.35, 4219.70] | 4669.68 [2231.12, 10050.39] | <0.001 | nonnorm |
| ClBPA\_ngPERg\_spot (median [IQR]) | 89.36 [58.66, 159.20] | 83.42 [58.23, 153.36] | 0.835 | nonnorm |
| Cl35\_2BPA\_ngPERg\_spot (median [IQR]) | 87.07 [51.67, 182.58] | 78.70 [50.15, 147.73] | 0.359 | nonnorm |
| Cl33\_2BPA\_ngPERg\_spot (median [IQR]) | 17.32 [7.20, 39.27] | 23.81 [9.78, 64.29] | 0.052 | nonnorm |
| ClxBPA\_ngPERg\_spot (median [IQR]) | 199.19 [120.17, 375.68] | 200.90 [125.18, 393.60] | 0.961 | nonnorm |

Demographics by country

|  | CY | RO | p | test |
| --- | --- | --- | --- | --- |
| n | 122 | 90 |  |  |
| age (mean (sd)) | 50.88 (12.27) | 46.96 (12.92) | 0.026 |  |
| weight (mean (sd)) | 67.04 (12.16) | 72.93 (16.11) | 0.003 |  |
| heightM (mean (sd)) | 1.63 (0.06) | 1.65 (0.06) | 0.018 |  |
| BMI (mean (sd)) | 25.39 (4.56) | 27.00 (6.46) | 0.037 |  |
| BMI\_cat (%) |  |  | 0.046 | exact |
| Underweight | 2 ( 1.7) | 3 ( 3.4) |  |  |
| Normal | 56 (47.9) | 35 (39.3) |  |  |
| Overweight | 39 (33.3) | 22 (24.7) |  |  |
| Obese | 20 (17.1) | 29 (32.6) |  |  |
| smokst (%) |  |  | 0.186 |  |
| Never | 94 (77.0) | 63 (73.3) |  |  |
| Currently | 17 (13.9) | 19 (22.1) |  |  |
| Past | 11 ( 9.0) | 4 ( 4.7) |  |  |
| alccons (%) |  |  | 0.002 | exact |
| Never/Rarely | 95 (77.9) | 80 (94.1) |  |  |
| Weekend | 20 (16.4) | 5 ( 5.9) |  |  |
| Often | 7 ( 5.7) | 0 ( 0.0) |  |  |
| marstat (%) |  |  | 0.101 | exact |
| Single | 13 (10.7) | 12 (14.0) |  |  |
| Married | 99 (81.1) | 58 (67.4) |  |  |
| Divorced | 6 ( 4.9) | 7 ( 8.1) |  |  |
| Widow | 4 ( 3.3) | 8 ( 9.3) |  |  |
| Other | 0 ( 0.0) | 1 ( 1.2) |  |  |
| edu (%) |  |  | 0.247 | exact |
| Primary | 16 (13.1) | 12 (14.1) |  |  |
| Secondary | 43 (35.2) | 35 (41.2) |  |  |
| University | 63 (51.6) | 36 (42.4) |  |  |
| Other | 0 ( 0.0) | 2 ( 2.4) |  |  |

Determinants of exposure by country

|  | CY | RO | p | test |
| --- | --- | --- | --- | --- |
| n | 122 | 90 |  |  |
| daily20lglass (median [IQR]) | 0.00 [0.00, 0.91] | 0.00 [0.00, 0.00] | 0.004 | nonnorm |
| MwaveFrw (median [IQR]) | 0.00 [0.00, 0.00] | 0.00 [0.00, 0.00] | 0.031 | nonnorm |
| weekPORTIONScanned (median [IQR]) | 1.88 [0.75, 3.01] | 0.50 [0.00, 2.00] | <0.001 | nonnorm |
| weekMINScleaning (median [IQR]) | 308.62 [177.50, 504.38] | 211.25 [115.00, 355.00] | 0.002 | nonnorm |
| weekPORTIONSpcps (median [IQR]) | 86.20 [65.86, 103.00] | 50.50 [32.50, 65.00] | <0.001 | nonnorm |
| perfwc (median [IQR]) | 7.00 [1.50, 7.00] | 7.00 [1.00, 7.00] | 0.860 | nonnorm |
| weekPORTIONSdeo (median [IQR]) | 7.00 [3.25, 7.00] | 7.00 [3.00, 7.00] | 0.554 | nonnorm |
| weekPORTIONScosm (median [IQR]) | 7.00 [0.18, 22.25] | 7.00 [0.00, 21.12] | 0.296 | nonnorm |

Hormones and iodine levels by country

|  | CY | RO | p | test |
| --- | --- | --- | --- | --- |
| n | 122 | 90 |  |  |
| tsh (median [IQR]) | 1.51 [1.06, 2.00] | 1.68 [1.05, 2.55] | 0.122 | nonnorm |
| ft4 (median [IQR]) | 12.70 [11.80, 14.00] | 14.71 [13.10, 16.10] | <0.001 | nonnorm |
| iod\_ugPERlt\_spot (median [IQR]) | 94.30 [40.40, 190.00] | 118.40 [60.53, 182.80] | 0.212 | nonnorm |
| iod\_ugPERg\_spot (median [IQR]) | 114.30 [67.40, 185.20] | 125.72 [84.03, 174.82] | 0.548 | nonnorm |

## 4.2 Logistic regressions

### 4.2.1 Table S6, Table S7

- Demographics - table S6 - A

  |  |  |  |  |  |  |  |  |  |  |  |  |  |  |  |  |  |  |  |  |  |  |  |  |  |  |  |  |  |  |  |  |  |
  | --- | --- | --- | --- | --- | --- | --- | --- | --- | --- | --- | --- | --- | --- | --- | --- | --- | --- | --- | --- | --- | --- | --- | --- | --- | --- | --- | --- | --- | --- | --- | --- | --- |
  |  |  | status01 | | |  | status01 | | |  | status01 | | |  | status01 | | |  | status01 | | |  | status01 | | |  | status01 | | |  | status01 | | |
  |  |  | OR | CI | p |  | OR | CI | p |  | OR | CI | p |  | OR | CI | p |  | OR | CI | p |  | OR | CI | p |  | OR | CI | p |  | OR | CI | p |
  | (Intercept) |  | 0.19 | 0.06 – 0.56 | **.003** |  | 0.14 | 0.03 – 0.55 | **.006** |  | 455.50 | 0.31 – 831313.64 | .103 |  | 0.09 | 0.02 – 0.37 | **.001** |  | 0.94 | 0.69 – 1.28 | .690 |  | 1.01 | 0.75 – 1.36 | .940 |  | 0.32 | 0.12 – 0.75 | **.014** |  | 3.00 | 1.34 – 7.62 | **.012** |
  | age |  | 1.03 | 1.01 – 1.06 | **.003** |  |  |  |  |  |  |  |  |  |  |  |  |  |  |  |  |  |  |  |  |  |  |  |  |  |  |  |  |
  | weight |  |  |  |  |  | 1.03 | 1.01 – 1.05 | **.005** |  |  |  |  |  |  |  |  |  |  |  |  |  |  |  |  |  |  |  |  |  |  |  |  |
  | heightM |  |  |  |  |  |  |  |  |  | 0.02 | 0.00 – 2.02 | .103 |  |  |  |  |  |  |  |  |  |  |  |  |  |  |  |  |  |  |  |  |
  | BMI |  |  |  |  |  |  |  |  |  |  |  |  |  | 1.10 | 1.04 – 1.16 | **.001** |  |  |  |  |  |  |  |  |  |  |  |  |  |  |  |  |
  | smokstCurrently |  |  |  |  |  |  |  |  |  |  |  |  |  |  |  |  |  | 1.07 | 0.51 – 2.21 | .863 |  |  |  |  |  |  |  |  |  |  |  |  |
  | smokstPast |  |  |  |  |  |  |  |  |  |  |  |  |  |  |  |  |  | 1.60 | 0.55 – 4.97 | .394 |  |  |  |  |  |  |  |  |  |  |  |  |
  | alcconsWeekend |  |  |  |  |  |  |  |  |  |  |  |  |  |  |  |  |  |  |  |  |  | 0.78 | 0.33 – 1.80 | .557 |  |  |  |  |  |  |  |  |
  | alcconsOften |  |  |  |  |  |  |  |  |  |  |  |  |  |  |  |  |  |  |  |  |  | 0.74 | 0.14 – 3.46 | .701 |  |  |  |  |  |  |  |  |
  | marstatMarried |  |  |  |  |  |  |  |  |  |  |  |  |  |  |  |  |  |  |  |  |  |  |  |  |  | 3.74 | 1.49 – 10.72 | **.008** |  |  |  |  |
  | marstatDivorced |  |  |  |  |  |  |  |  |  |  |  |  |  |  |  |  |  |  |  |  |  |  |  |  |  | 1.41 | 0.30 – 6.26 | .654 |  |  |  |  |
  | marstatWidow |  |  |  |  |  |  |  |  |  |  |  |  |  |  |  |  |  |  |  |  |  |  |  |  |  | 4.43 | 1.05 – 20.69 | **.047** |  |  |  |  |
  | marstatOther |  |  |  |  |  |  |  |  |  |  |  |  |  |  |  |  |  |  |  |  |  |  |  |  |  | 6707569.93 | 0.00 – NA | .986 |  |  |  |  |
  | eduSecondary |  |  |  |  |  |  |  |  |  |  |  |  |  |  |  |  |  |  |  |  |  |  |  |  |  |  |  |  |  | 0.35 | 0.13 – 0.89 | **.033** |
  | eduUniversity |  |  |  |  |  |  |  |  |  |  |  |  |  |  |  |  |  |  |  |  |  |  |  |  |  |  |  |  |  | 0.23 | 0.08 – 0.56 | **.002** |
  | eduOther |  |  |  |  |  |  |  |  |  |  |  |  |  |  |  |  |  |  |  |  |  |  |  |  |  |  |  |  |  | 1919270.95 | 0.00 – NA | .989 |
  | Observations |  | 212 | | |  | 209 | | |  | 208 | | |  | 206 | | |  | 208 | | |  | 207 | | |  | 208 | | |  | 207 | | |
- Questionnaire exposures - table S6 - A

  |  |  |  |  |  |  |  |  |  |  |  |  |  |  |  |  |  |  |  |  |  |  |  |  |  |  |  |  |  |  |  |  |  |
  | --- | --- | --- | --- | --- | --- | --- | --- | --- | --- | --- | --- | --- | --- | --- | --- | --- | --- | --- | --- | --- | --- | --- | --- | --- | --- | --- | --- | --- | --- | --- | --- | --- |
  |  |  | status01 | | |  | status01 | | |  | status01 | | |  | status01 | | |  | status01 | | |  | status01 | | |  | status01 | | |  | status01 | | |
  |  |  | OR | CI | p |  | OR | CI | p |  | OR | CI | p |  | OR | CI | p |  | OR | CI | p |  | OR | CI | p |  | OR | CI | p |  | OR | CI | p |
  | (Intercept) |  | 1.15 | 0.86 – 1.54 | .354 |  | 1.02 | 0.76 – 1.35 | .906 |  | 1.33 | 0.92 – 1.96 | .138 |  | 0.78 | 0.48 – 1.26 | .314 |  | 1.91 | 0.95 – 3.94 | .073 |  | 2.01 | 1.23 – 3.35 | **.006** |  | 1.37 | 0.81 – 2.35 | .248 |  | 1.62 | 1.10 – 2.41 | **.015** |
  | daily20lglass |  | 0.86 | 0.72 – 1.00 | .060 |  |  |  |  |  |  |  |  |  |  |  |  |  |  |  |  |  |  |  |  |  |  |  |  |  |  |  |  |
  | MwaveFrw |  |  |  |  |  | 1.00 | 0.87 – 1.16 | .964 |  |  |  |  |  |  |  |  |  |  |  |  |  |  |  |  |  |  |  |  |  |  |  |  |
  | weekPORTIONScanned |  |  |  |  |  |  |  |  |  | 0.84 | 0.73 – 0.95 | **.013** |  |  |  |  |  |  |  |  |  |  |  |  |  |  |  |  |  |  |  |  |
  | weekMINScleaning |  |  |  |  |  |  |  |  |  |  |  |  |  | 1.00 | 1.00 – 1.00 | .327 |  |  |  |  |  |  |  |  |  |  |  |  |  |  |  |  |
  | weekPORTIONSpcps |  |  |  |  |  |  |  |  |  |  |  |  |  |  |  |  |  | 0.99 | 0.98 – 1.00 | .090 |  |  |  |  |  |  |  |  |  |  |  |  |
  | perfwc |  |  |  |  |  |  |  |  |  |  |  |  |  |  |  |  |  |  |  |  |  | 0.87 | 0.80 – 0.95 | **.002** |  |  |  |  |  |  |  |  |
  | weekPORTIONSdeo |  |  |  |  |  |  |  |  |  |  |  |  |  |  |  |  |  |  |  |  |  |  |  |  |  | 0.95 | 0.87 – 1.03 | .203 |  |  |  |  |
  | weekPORTIONScosm |  |  |  |  |  |  |  |  |  |  |  |  |  |  |  |  |  |  |  |  |  |  |  |  |  |  |  |  |  | 0.96 | 0.94 – 0.98 | **.001** |
  | Observations |  | 209 | | |  | 208 | | |  | 193 | | |  | 185 | | |  | 168 | | |  | 207 | | |  | 200 | | |  | 202 | | |
- Hormone levels and iodine - table S6 - A

  |  |  |  |  |  |  |  |  |  |  |  |  |  |
  | --- | --- | --- | --- | --- | --- | --- | --- | --- | --- | --- | --- | --- |
  |  |  | status01 | | |  | status01 | | |  | status01 | | |
  |  |  | OR | CI | p |  | OR | CI | p |  | OR | CI | p |
  | (Intercept) |  | 1.26 | 0.91 – 1.76 | .171 |  | 0.52 | 0.00 – 59.61 | .784 |  | 2.47 | 0.75 – 8.44 | .140 |
  | ln\_tsh |  | 0.57 | 0.36 – 0.88 | **.014** |  |  |  |  |  |  |  |  |
  | ln\_ft4 |  |  |  |  |  | 1.28 | 0.21 – 7.98 | .790 |  |  |  |  |
  | ln\_iod\_ugPERlt\_spot |  |  |  |  |  |  |  |  |  | 0.82 | 0.62 – 1.06 | .129 |
  | Observations |  | 212 | | |  | 210 | | |  | 208 | | |

Study-site adjusted univariate logistic regression

- Demographics - table S6 - B

  |  |  |  |  |  |  |  |  |  |  |  |  |  |  |  |  |  |  |  |  |  |  |  |  |  |  |  |  |  |  |  |  |  |
  | --- | --- | --- | --- | --- | --- | --- | --- | --- | --- | --- | --- | --- | --- | --- | --- | --- | --- | --- | --- | --- | --- | --- | --- | --- | --- | --- | --- | --- | --- | --- | --- | --- |
  |  |  | status01 | | |  | status01 | | |  | status01 | | |  | status01 | | |  | status01 | | |  | status01 | | |  | status01 | | |  | status01 | | |
  |  |  | OR | CI | p |  | OR | CI | p |  | OR | CI | p |  | OR | CI | p |  | OR | CI | p |  | OR | CI | p |  | OR | CI | p |  | OR | CI | p |
  | (Intercept) |  | 0.13 | 0.04 – 0.43 | **.001** |  | 0.14 | 0.03 – 0.55 | **.006** |  | 1091.28 | 0.66 – 2395330.40 | .068 |  | 0.09 | 0.02 – 0.36 | **.001** |  | 0.83 | 0.57 – 1.23 | .358 |  | 0.92 | 0.62 – 1.36 | .663 |  | 0.27 | 0.09 – 0.66 | **.007** |  | 2.74 | 1.18 – 7.16 | **.026** |
  | countryRO |  | 1.61 | 0.91 – 2.87 | .103 |  | 1.16 | 0.66 – 2.06 | .604 |  | 1.57 | 0.89 – 2.78 | .119 |  | 1.22 | 0.69 – 2.17 | .490 |  | 1.34 | 0.77 – 2.35 | .306 |  | 1.24 | 0.70 – 2.20 | .455 |  | 1.40 | 0.79 – 2.52 | .252 |  | 1.24 | 0.70 – 2.21 | .463 |
  | age |  | 1.04 | 1.02 – 1.06 | **.001** |  |  |  |  |  |  |  |  |  |  |  |  |  |  |  |  |  |  |  |  |  |  |  |  |  |  |  |  |
  | weight |  |  |  |  |  | 1.03 | 1.01 – 1.05 | **.007** |  |  |  |  |  |  |  |  |  |  |  |  |  |  |  |  |  |  |  |  |  |  |  |  |
  | heightM |  |  |  |  |  |  |  |  |  | 0.01 | 0.00 – 1.17 | .062 |  |  |  |  |  |  |  |  |  |  |  |  |  |  |  |  |  |  |  |  |
  | BMI |  |  |  |  |  |  |  |  |  |  |  |  |  | 1.10 | 1.04 – 1.16 | **.001** |  |  |  |  |  |  |  |  |  |  |  |  |  |  |  |  |
  | smokstCurrently |  |  |  |  |  |  |  |  |  |  |  |  |  |  |  |  |  | 1.03 | 0.49 – 2.14 | .942 |  |  |  |  |  |  |  |  |  |  |  |  |
  | smokstPast |  |  |  |  |  |  |  |  |  |  |  |  |  |  |  |  |  | 1.67 | 0.57 – 5.21 | .356 |  |  |  |  |  |  |  |  |  |  |  |  |
  | alcconsWeekend |  |  |  |  |  |  |  |  |  |  |  |  |  |  |  |  |  |  |  |  |  | 0.82 | 0.34 – 1.93 | .652 |  |  |  |  |  |  |  |  |
  | alcconsOften |  |  |  |  |  |  |  |  |  |  |  |  |  |  |  |  |  |  |  |  |  | 0.82 | 0.15 – 3.90 | .800 |  |  |  |  |  |  |  |  |
  | marstatMarried |  |  |  |  |  |  |  |  |  |  |  |  |  |  |  |  |  |  |  |  |  |  |  |  |  | 3.92 | 1.55 – 11.31 | **.006** |  |  |  |  |
  | marstatDivorced |  |  |  |  |  |  |  |  |  |  |  |  |  |  |  |  |  |  |  |  |  |  |  |  |  | 1.38 | 0.29 – 6.18 | .672 |  |  |  |  |
  | marstatWidow |  |  |  |  |  |  |  |  |  |  |  |  |  |  |  |  |  |  |  |  |  |  |  |  |  | 4.20 | 0.99 – 19.71 | .057 |  |  |  |  |
  | marstatOther |  |  |  |  |  |  |  |  |  |  |  |  |  |  |  |  |  |  |  |  |  |  |  |  |  | 5665074.60 | 0.00 – NA | .986 |  |  |  |  |
  | eduSecondary |  |  |  |  |  |  |  |  |  |  |  |  |  |  |  |  |  |  |  |  |  |  |  |  |  |  |  |  |  | 0.35 | 0.12 – 0.88 | **.032** |
  | eduUniversity |  |  |  |  |  |  |  |  |  |  |  |  |  |  |  |  |  |  |  |  |  |  |  |  |  |  |  |  |  | 0.23 | 0.08 – 0.56 | **.002** |
  | eduOther |  |  |  |  |  |  |  |  |  |  |  |  |  |  |  |  |  |  |  |  |  |  |  |  |  |  |  |  |  | 1692079.85 | 0.00 – NA | .989 |
  | Observations |  | 212 | | |  | 209 | | |  | 208 | | |  | 206 | | |  | 208 | | |  | 207 | | |  | 208 | | |  | 207 | | |
- Questionnaire exposures - table S6 - B

  |  |  |  |  |  |  |  |  |  |  |  |  |  |  |  |  |  |  |  |  |  |  |  |  |  |  |  |  |  |  |  |  |  |
  | --- | --- | --- | --- | --- | --- | --- | --- | --- | --- | --- | --- | --- | --- | --- | --- | --- | --- | --- | --- | --- | --- | --- | --- | --- | --- | --- | --- | --- | --- | --- | --- | --- |
  |  |  | status01 | | |  | status01 | | |  | status01 | | |  | status01 | | |  | status01 | | |  | status01 | | |  | status01 | | |  | status01 | | |
  |  |  | OR | CI | p |  | OR | CI | p |  | OR | CI | p |  | OR | CI | p |  | OR | CI | p |  | OR | CI | p |  | OR | CI | p |  | OR | CI | p |
  | (Intercept) |  | 1.05 | 0.71 – 1.56 | .802 |  | 0.88 | 0.61 – 1.28 | .515 |  | 1.25 | 0.79 – 2.04 | .348 |  | 0.68 | 0.37 – 1.21 | .192 |  | 1.55 | 0.59 – 4.16 | .374 |  | 1.70 | 1.00 – 2.96 | .055 |  | 1.16 | 0.66 – 2.07 | .607 |  | 1.43 | 0.91 – 2.26 | .123 |
  | country (RO) |  | 1.21 | 0.69 – 2.13 | .515 |  | 1.39 | 0.80 – 2.43 | .248 |  | 1.14 | 0.62 – 2.09 | .679 |  | 1.31 | 0.71 – 2.43 | .396 |  | 1.26 | 0.60 – 2.65 | .544 |  | 1.55 | 0.87 – 2.76 | .137 |  | 1.56 | 0.89 – 2.78 | .124 |  | 1.36 | 0.76 – 2.44 | .297 |
  | daily20lglass |  | 0.87 | 0.73 – 1.01 | .086 |  |  |  |  |  |  |  |  |  |  |  |  |  |  |  |  |  |  |  |  |  |  |  |  |  |  |  |  |
  | MwaveFrw |  |  |  |  |  | 1.01 | 0.87 – 1.17 | .882 |  |  |  |  |  |  |  |  |  |  |  |  |  |  |  |  |  |  |  |  |  |  |  |  |
  | weekPORTIONScanned |  |  |  |  |  |  |  |  |  | 0.85 | 0.73 – 0.96 | **.018** |  |  |  |  |  |  |  |  |  |  |  |  |  |  |  |  |  |  |  |  |
  | weekMINScleaning |  |  |  |  |  |  |  |  |  |  |  |  |  | 1.00 | 1.00 – 1.00 | .249 |  |  |  |  |  |  |  |  |  |  |  |  |  |  |  |  |
  | weekPORTIONSpcps |  |  |  |  |  |  |  |  |  |  |  |  |  |  |  |  |  | 0.99 | 0.98 – 1.00 | .257 |  |  |  |  |  |  |  |  |  |  |  |  |
  | perfwc |  |  |  |  |  |  |  |  |  |  |  |  |  |  |  |  |  |  |  |  |  | 0.87 | 0.80 – 0.95 | **.001** |  |  |  |  |  |  |  |  |
  | weekPORTIONSdeo |  |  |  |  |  |  |  |  |  |  |  |  |  |  |  |  |  |  |  |  |  |  |  |  |  | 0.94 | 0.87 – 1.02 | .169 |  |  |  |  |
  | weekPORTIONScosm |  |  |  |  |  |  |  |  |  |  |  |  |  |  |  |  |  |  |  |  |  |  |  |  |  |  |  |  |  | 0.96 | 0.94 – 0.98 | **.001** |
  | Observations |  | 209 | | |  | 208 | | |  | 193 | | |  | 185 | | |  | 168 | | |  | 207 | | |  | 200 | | |  | 202 | | |
- Hormone levels and iodine - table S6 - B

  |  |  |  |  |  |  |  |  |  |  |  |  |  |
  | --- | --- | --- | --- | --- | --- | --- | --- | --- | --- | --- | --- | --- |
  |  |  | status01 | | |  | status01 | | |  | status01 | | |
  |  |  | OR | CI | p |  | OR | CI | p |  | OR | CI | p |
  | (Intercept) |  | 1.08 | 0.73 – 1.61 | .688 |  | 1.62 | 0.01 – 272.73 | .853 |  | 2.35 | 0.72 – 8.04 | .163 |
  | country (RO) |  | 1.49 | 0.85 – 2.63 | .166 |  | 1.46 | 0.80 – 2.67 | .219 |  | 1.41 | 0.81 – 2.48 | .231 |
  | ln\_tsh |  | 0.55 | 0.34 – 0.86 | **.010** |  |  |  |  |  |  |  |  |
  | ln\_ft4 |  |  |  |  |  | 0.78 | 0.10 – 5.70 | .802 |  |  |  |  |
  | ln\_iod\_ugPERlt\_spot |  |  |  |  |  |  |  |  |  | 0.80 | 0.61 – 1.04 | .097 |
  | Observations |  | 212 | | |  | 210 | | |  | 208 | | |
- BPF, BPA and ClxBPA - table S7

  |  |  |  |  |  |  |  |  |  |  |  |  |  |
  | --- | --- | --- | --- | --- | --- | --- | --- | --- | --- | --- | --- | --- |
  |  |  | status01 | | |  | status01 | | |  | status01 | | |
  |  |  | OR | CI | p |  | OR | CI | p |  | OR | CI | p |
  | (Intercept) |  | 70.67 | 6.36 – 957.34 | **.001** |  | 1.13 | 0.20 – 6.60 | .888 |  | 7.69 | 0.01 – 8670.21 | .554 |
  | country (RO) |  | 2.28 | 1.19 – 4.49 | **.015** |  | 1.27 | 0.73 – 2.22 | .406 |  | 1.34 | 0.75 – 2.41 | .322 |
  | ln\_creat\_gPERlt\_spot |  | 1.29 | 0.87 – 1.92 | .206 |  | 0.97 | 0.67 – 1.39 | .851 |  | 0.97 | 0.68 – 1.37 | .847 |
  | ln\_BPA\_ngPERlt\_spot |  | 0.56 | 0.40 – 0.77 | **<.001** |  |  |  |  |  |  |  |  |
  | ln\_BPF\_ngPERlt\_spot |  |  |  |  |  | 0.96 | 0.74 – 1.25 | .765 |  |  |  |  |
  | ln\_ClxBPA\_ngPERlt\_spot |  |  |  |  |  |  |  |  |  | 0.65 | 0.16 – 2.44 | .527 |
  | Observations |  | 206 | | |  | 206 | | |  | 206 | | |

### 4.2.2 Table 3

BPF, BPA and ClxBPA - table 3 - crude estimates

|  |  |  |  |  |  |  |  |  |  |  |  |  |
| --- | --- | --- | --- | --- | --- | --- | --- | --- | --- | --- | --- | --- |
|  |  | status01 | | |  | status01 | | |  | status01 | | |
|  |  | OR | CI | p |  | OR | CI | p |  | OR | CI | p |
| (Intercept) |  | 22.64 | 2.54 – 237.69 | **.007** |  | 1.30 | 0.24 – 7.37 | .761 |  | 3.39 | 0.00 – 2788.32 | .713 |
| ln\_creat\_gPERlt\_spot |  | 1.21 | 0.83 – 1.79 | .325 |  | 0.98 | 0.68 – 1.41 | .919 |  | 0.97 | 0.69 – 1.38 | .879 |
| ln\_BPA\_ngPERlt\_spot |  | 0.67 | 0.50 – 0.88 | **.006** |  |  |  |  |  |  |  |  |
| ln\_BPF\_ngPERlt\_spot |  |  |  |  |  | 0.96 | 0.73 – 1.24 | .731 |  |  |  |  |
| ln\_ClxBPA\_ngPERlt\_spot |  |  |  |  |  |  |  |  |  | 0.78 | 0.21 – 2.78 | .706 |
| Observations |  | 206 | | |  | 206 | | |  | 206 | | |

(A1) Adjusted for: age, BMI, TSH, FT4 - table 3

|  |  |  |  |  |  |  |  |  |  |  |  |  |
| --- | --- | --- | --- | --- | --- | --- | --- | --- | --- | --- | --- | --- |
|  |  | status01 | | |  | status01 | | |  | status01 | | |
|  |  | OR | CI | p |  | OR | CI | p |  | OR | CI | p |
| (Intercept) |  | 0.36 | 0.00 – 124.19 | .732 |  | 0.08 | 0.00 – 29.94 | .406 |  | 0.09 | 0.00 – 895.49 | .605 |
| age |  | 1.02 | 0.99 – 1.04 | .270 |  | 1.02 | 0.99 – 1.05 | .145 |  | 1.02 | 0.99 – 1.05 | .148 |
| BMI |  | 1.08 | 1.02 – 1.16 | **.014** |  | 1.08 | 1.01 – 1.15 | **.019** |  | 1.08 | 1.01 – 1.15 | **.018** |
| ln\_tsh |  | 0.48 | 0.28 – 0.81 | **.007** |  | 0.44 | 0.26 – 0.73 | **.002** |  | 0.44 | 0.26 – 0.73 | **.002** |
| ln\_ft4 |  | 1.26 | 0.15 – 10.56 | .830 |  | 1.00 | 0.12 – 8.09 | .997 |  | 1.01 | 0.13 – 8.13 | .990 |
| ln\_creat\_gPERlt\_spot |  | 1.27 | 0.84 – 1.94 | .266 |  | 1.11 | 0.74 – 1.66 | .613 |  | 1.10 | 0.75 – 1.63 | .619 |
| ln\_BPA\_ngPERlt\_spot |  | 0.76 | 0.56 – 1.03 | .085 |  |  |  |  |  |  |  |  |
| ln\_BPF\_ngPERlt\_spot |  |  |  |  |  | 0.98 | 0.73 – 1.31 | .902 |  |  |  |  |
| ln\_ClxBPA\_ngPERlt\_spot |  |  |  |  |  |  |  |  |  | 0.95 | 0.22 – 3.88 | .941 |
| Observations |  | 198 | | |  | 198 | | |  | 198 | | |

(A2) Adjusted for: study site, age, BMI, TSH, FT4 - table 3

|  |  |  |  |  |  |  |  |  |  |  |  |  |
| --- | --- | --- | --- | --- | --- | --- | --- | --- | --- | --- | --- | --- |
|  |  | status01 | | |  | status01 | | |  | status01 | | |
|  |  | OR | CI | p |  | OR | CI | p |  | OR | CI | p |
| (Intercept) |  | 12.72 | 0.02 – 11825.67 | .459 |  | 0.27 | 0.00 – 150.42 | .687 |  | 1.77 | 0.00 – 62411.79 | .913 |
| country (RO) |  | 2.38 | 1.11 – 5.26 | **.029** |  | 1.54 | 0.78 – 3.08 | .217 |  | 1.62 | 0.80 – 3.35 | .187 |
| age |  | 1.02 | 0.99 – 1.05 | .166 |  | 1.02 | 1.00 – 1.05 | .095 |  | 1.02 | 1.00 – 1.05 | .095 |
| BMI |  | 1.07 | 1.01 – 1.15 | **.034** |  | 1.07 | 1.01 – 1.15 | **.032** |  | 1.07 | 1.01 – 1.15 | **.030** |
| ln\_tsh |  | 0.48 | 0.27 – 0.80 | **.007** |  | 0.43 | 0.25 – 0.71 | **.002** |  | 0.43 | 0.25 – 0.71 | **.002** |
| ln\_ft4 |  | 0.47 | 0.04 – 4.76 | .520 |  | 0.58 | 0.06 – 5.55 | .633 |  | 0.54 | 0.05 – 5.31 | .599 |
| ln\_creat\_gPERlt\_spot |  | 1.34 | 0.88 – 2.08 | .180 |  | 1.09 | 0.72 – 1.64 | .683 |  | 1.10 | 0.74 – 1.63 | .636 |
| ln\_BPA\_ngPERlt\_spot |  | 0.65 | 0.46 – 0.91 | **.014** |  |  |  |  |  |  |  |  |
| ln\_BPF\_ngPERlt\_spot |  |  |  |  |  | 0.99 | 0.74 – 1.32 | .939 |  |  |  |  |
| ln\_ClxBPA\_ngPERlt\_spot |  |  |  |  |  |  |  |  |  | 0.70 | 0.14 – 3.09 | .647 |
| Observations |  | 198 | | |  | 198 | | |  | 198 | | |

(B1) Adjusted for: age, BMI, TSH, FT4, spot iodine (ΞΌg/L) - table 3

|  |  |  |  |  |  |  |  |  |  |  |  |  |
| --- | --- | --- | --- | --- | --- | --- | --- | --- | --- | --- | --- | --- |
|  |  | status01 | | |  | status01 | | |  | status01 | | |
|  |  | OR | CI | p |  | OR | CI | p |  | OR | CI | p |
| (Intercept) |  | 1.25 | 0.00 – 606.31 | .942 |  | 0.51 | 0.00 – 305.50 | .836 |  | 0.29 | 0.00 – 3440.14 | .795 |
| age |  | 1.02 | 0.99 – 1.05 | .201 |  | 1.02 | 1.00 – 1.05 | .105 |  | 1.02 | 1.00 – 1.05 | .104 |
| BMI |  | 1.08 | 1.02 – 1.16 | **.017** |  | 1.08 | 1.01 – 1.15 | **.022** |  | 1.08 | 1.01 – 1.15 | **.022** |
| ln\_tsh |  | 0.50 | 0.29 – 0.83 | **.009** |  | 0.46 | 0.27 – 0.77 | **.004** |  | 0.46 | 0.27 – 0.77 | **.004** |
| ln\_ft4 |  | 1.10 | 0.13 – 9.29 | .931 |  | 0.89 | 0.11 – 7.23 | .910 |  | 0.90 | 0.11 – 7.27 | .919 |
| ln\_iod\_ugPERlt\_spot |  | 0.76 | 0.49 – 1.15 | .196 |  | 0.71 | 0.47 – 1.07 | .107 |  | 0.71 | 0.47 – 1.07 | .108 |
| ln\_creat\_gPERlt\_spot |  | 1.62 | 0.93 – 2.87 | .093 |  | 1.54 | 0.88 – 2.75 | .138 |  | 1.52 | 0.88 – 2.68 | .139 |
| ln\_BPA\_ngPERlt\_spot |  | 0.80 | 0.58 – 1.08 | .148 |  |  |  |  |  |  |  |  |
| ln\_BPF\_ngPERlt\_spot |  |  |  |  |  | 0.98 | 0.73 – 1.30 | .867 |  |  |  |  |
| ln\_ClxBPA\_ngPERlt\_spot |  |  |  |  |  |  |  |  |  | 1.07 | 0.24 – 4.47 | .924 |
| Observations |  | 198 | | |  | 198 | | |  | 198 | | |

(B2) Adjusted for: study site age, BMI, TSH, FT4, spot iodine (ΞΌg/L) - table 3

|  |  |  |  |  |  |  |  |  |  |  |  |  |
| --- | --- | --- | --- | --- | --- | --- | --- | --- | --- | --- | --- | --- |
|  |  | status01 | | |  | status01 | | |  | status01 | | |
|  |  | OR | CI | p |  | OR | CI | p |  | OR | CI | p |
| (Intercept) |  | 62.23 | 0.05 – 91559.40 | .258 |  | 2.85 | 0.00 – 2940.48 | .766 |  | 9.67 | 0.00 – 462152.40 | .671 |
| country (RO) |  | 2.46 | 1.14 – 5.50 | **.025** |  | 1.67 | 0.83 – 3.40 | .151 |  | 1.73 | 0.84 – 3.63 | .141 |
| age |  | 1.02 | 0.99 – 1.05 | .115 |  | 1.03 | 1.00 – 1.06 | .059 |  | 1.03 | 1.00 – 1.06 | .060 |
| BMI |  | 1.07 | 1.00 – 1.15 | **.042** |  | 1.07 | 1.00 – 1.14 | **.042** |  | 1.07 | 1.00 – 1.15 | **.040** |
| ln\_tsh |  | 0.49 | 0.28 – 0.83 | **.010** |  | 0.45 | 0.26 – 0.75 | **.003** |  | 0.45 | 0.26 – 0.75 | **.003** |
| ln\_ft4 |  | 0.37 | 0.03 – 3.93 | .413 |  | 0.44 | 0.04 – 4.44 | .489 |  | 0.43 | 0.04 – 4.33 | .473 |
| ln\_iod\_ugPERlt\_spot |  | 0.74 | 0.48 – 1.12 | .163 |  | 0.69 | 0.45 – 1.03 | .077 |  | 0.69 | 0.45 – 1.04 | .083 |
| ln\_creat\_gPERlt\_spot |  | 1.75 | 1.00 – 3.14 | .056 |  | 1.56 | 0.89 – 2.81 | .129 |  | 1.55 | 0.90 – 2.75 | .121 |
| ln\_BPA\_ngPERlt\_spot |  | 0.67 | 0.47 – 0.94 | **.025** |  |  |  |  |  |  |  |  |
| ln\_BPF\_ngPERlt\_spot |  |  |  |  |  | 0.98 | 0.73 – 1.31 | .900 |  |  |  |  |
| ln\_ClxBPA\_ngPERlt\_spot |  |  |  |  |  |  |  |  |  | 0.77 | 0.15 – 3.47 | .744 |
| Observations |  | 198 | | |  | 198 | | |  | 198 | | |

### 4.2.3 Table S8

(A1) Adjusted for: age, BMI, TSH, FT4 - table S8 - BPA

|  |  |  |  |  |
| --- | --- | --- | --- | --- |
|  |  | status01 | | |
|  |  | OR | CI | p |
| (Intercept) |  | 0.07 | 0.00 – 34.86 | .401 |
| age |  | 1.02 | 0.99 – 1.05 | .197 |
| BMI |  | 1.07 | 1.00 – 1.15 | **.045** |
| ln\_tsh |  | 0.51 | 0.30 – 0.86 | **.015** |
| ln\_ft4 |  | 1.66 | 0.19 – 15.00 | .646 |
| ln\_creat\_gPERlt\_spot |  | 1.13 | 0.72 – 1.76 | .596 |
| ln\_BPA\_ngPERlt\_spot |  | 0.88 | 0.58 – 1.33 | .553 |
| Observations |  | 177 | | |

(A2) Adjusted for: study site, age, BMI, TSH, FT4 - table S8 - BPA

|  |  |  |  |  |
| --- | --- | --- | --- | --- |
|  |  | status01 | | |
|  |  | OR | CI | p |
| (Intercept) |  | 3.52 | 0.00 – 4918.44 | .731 |
| country (RO) |  | 2.52 | 1.17 – 5.62 | **.020** |
| age |  | 1.02 | 0.99 – 1.05 | .110 |
| BMI |  | 1.06 | 0.99 – 1.14 | .100 |
| ln\_tsh |  | 0.50 | 0.28 – 0.85 | **.013** |
| ln\_ft4 |  | 0.57 | 0.05 – 6.31 | .648 |
| ln\_creat\_gPERlt\_spot |  | 1.20 | 0.76 – 1.90 | .433 |
| ln\_BPA\_ngPERlt\_spot |  | 0.73 | 0.46 – 1.14 | .166 |
| Observations |  | 177 | | |

(B1) Adjusted for: age, BMI, TSH, FT4, spot iodine (ΞΌg/L) - table S8 - BPA

|  |  |  |  |  |
| --- | --- | --- | --- | --- |
|  |  | status01 | | |
|  |  | OR | CI | p |
| (Intercept) |  | 0.21 | 0.00 – 150.72 | .638 |
| age |  | 1.02 | 0.99 – 1.05 | .156 |
| BMI |  | 1.07 | 1.00 – 1.15 | **.049** |
| ln\_tsh |  | 0.53 | 0.30 – 0.89 | **.020** |
| ln\_ft4 |  | 1.45 | 0.16 – 13.23 | .740 |
| ln\_iod\_ugPERlt\_spot |  | 0.78 | 0.50 – 1.21 | .277 |
| ln\_creat\_gPERlt\_spot |  | 1.39 | 0.78 – 2.51 | .270 |
| ln\_BPA\_ngPERlt\_spot |  | 0.92 | 0.60 – 1.40 | .690 |
| Observations |  | 177 | | |

(B2) Adjusted for: study site age, BMI, TSH, FT4, spot iodine (ΞΌg/L) - table S8 - BPA

|  |  |  |  |  |
| --- | --- | --- | --- | --- |
|  |  | status01 | | |
|  |  | OR | CI | p |
| (Intercept) |  | 14.85 | 0.01 – 34180.50 | .488 |
| country (RO) |  | 2.60 | 1.19 – 5.85 | **.018** |
| age |  | 1.03 | 1.00 – 1.06 | .082 |
| BMI |  | 1.06 | 0.99 – 1.13 | .112 |
| ln\_tsh |  | 0.52 | 0.29 – 0.88 | **.019** |
| ln\_ft4 |  | 0.46 | 0.04 – 5.25 | .532 |
| ln\_iod\_ugPERlt\_spot |  | 0.77 | 0.49 – 1.18 | .237 |
| ln\_creat\_gPERlt\_spot |  | 1.51 | 0.84 – 2.76 | .176 |
| ln\_BPA\_ngPERlt\_spot |  | 0.76 | 0.48 – 1.18 | .223 |
| Observations |  | 177 | | |

(A1) Adjusted for: age, BMI, TSH, FT4 - table S8 - BPF

|  |  |  |  |  |
| --- | --- | --- | --- | --- |
|  |  | status01 | | |
|  |  | OR | CI | p |
| (Intercept) |  | 0.02 | 0.00 – 17.83 | .254 |
| age |  | 1.03 | 1.00 – 1.06 | .088 |
| BMI |  | 1.05 | 0.98 – 1.13 | .175 |
| ln\_tsh |  | 0.48 | 0.27 – 0.82 | **.009** |
| ln\_ft4 |  | 0.95 | 0.10 – 9.09 | .967 |
| ln\_creat\_gPERlt\_spot |  | 1.14 | 0.74 – 1.76 | .552 |
| ln\_BPF\_ngPERlt\_spot |  | 1.39 | 0.82 – 2.38 | .222 |
| Observations |  | 177 | | |

(A2) Adjusted for: study site, age, BMI, TSH, FT4 - table S8 - BPF

|  |  |  |  |  |
| --- | --- | --- | --- | --- |
|  |  | status01 | | |
|  |  | OR | CI | p |
| (Intercept) |  | 0.06 | 0.00 – 91.57 | .457 |
| country (RO) |  | 1.69 | 0.81 – 3.57 | .164 |
| age |  | 1.03 | 1.00 – 1.06 | .053 |
| BMI |  | 1.04 | 0.97 – 1.12 | .253 |
| ln\_tsh |  | 0.47 | 0.27 – 0.80 | **.007** |
| ln\_ft4 |  | 0.51 | 0.04 – 5.74 | .582 |
| ln\_creat\_gPERlt\_spot |  | 1.09 | 0.71 – 1.70 | .689 |
| ln\_BPF\_ngPERlt\_spot |  | 1.42 | 0.84 – 2.44 | .200 |
| Observations |  | 177 | | |

(B1) Adjusted for: age, BMI, TSH, FT4, spot iodine (ΞΌg/L) - table S8 - BPF

|  |  |  |  |  |
| --- | --- | --- | --- | --- |
|  |  | status01 | | |
|  |  | OR | CI | p |
| (Intercept) |  | 0.10 | 0.00 – 163.16 | .546 |
| age |  | 1.03 | 1.00 – 1.06 | .061 |
| BMI |  | 1.05 | 0.98 – 1.13 | .197 |
| ln\_tsh |  | 0.50 | 0.29 – 0.86 | **.014** |
| ln\_ft4 |  | 0.85 | 0.09 – 8.31 | .891 |
| ln\_iod\_ugPERlt\_spot |  | 0.69 | 0.45 – 1.05 | .090 |
| ln\_creat\_gPERlt\_spot |  | 1.64 | 0.90 – 3.06 | .110 |
| ln\_BPF\_ngPERlt\_spot |  | 1.41 | 0.84 – 2.43 | .203 |
| Observations |  | 177 | | |

(B2) Adjusted for: study site age, BMI, TSH, FT4, spot iodine (ΞΌg/L) - table S8 - BPF

|  |  |  |  |  |
| --- | --- | --- | --- | --- |
|  |  | status01 | | |
|  |  | OR | CI | p |
| (Intercept) |  | 0.61 | 0.00 – 1515.15 | .902 |
| country (RO) |  | 1.83 | 0.87 – 3.95 | .115 |
| age |  | 1.03 | 1.00 – 1.07 | **.031** |
| BMI |  | 1.04 | 0.97 – 1.12 | .298 |
| ln\_tsh |  | 0.49 | 0.28 – 0.84 | **.012** |
| ln\_ft4 |  | 0.39 | 0.03 – 4.69 | .462 |
| ln\_iod\_ugPERlt\_spot |  | 0.67 | 0.43 – 1.02 | .064 |
| ln\_creat\_gPERlt\_spot |  | 1.62 | 0.89 – 3.04 | .119 |
| ln\_BPF\_ngPERlt\_spot |  | 1.44 | 0.85 – 2.50 | .179 |
| Observations |  | 177 | | |

(A1) Adjusted for: age, BMI, TSH, FT4 - table S8 - ClxBPA

|  |  |  |  |  |
| --- | --- | --- | --- | --- |
|  |  | status01 | | |
|  |  | OR | CI | p |
| (Intercept) |  | 2.08 | 0.00 – 379132.11 | .905 |
| age |  | 1.02 | 0.99 – 1.05 | .308 |
| BMI |  | 1.09 | 1.02 – 1.17 | **.013** |
| ln\_tsh |  | 0.49 | 0.28 – 0.83 | **.010** |
| ln\_ft4 |  | 1.26 | 0.15 – 11.03 | .835 |
| ln\_creat\_gPERlt\_spot |  | 1.19 | 0.80 – 1.79 | .390 |
| ln\_ClxBPA\_ngPERlt\_spot |  | 0.46 | 0.05 – 3.95 | .481 |
| Observations |  | 179 | | |

(A2) Adjusted for: study site, age, BMI, TSH, FT4 - table S8 - ClxBPA

|  |  |  |  |  |
| --- | --- | --- | --- | --- |
|  |  | status01 | | |
|  |  | OR | CI | p |
| (Intercept) |  | 258.19 | 0.00 – 372265383.87 | .435 |
| country (RO) |  | 1.74 | 0.81 – 3.82 | .161 |
| age |  | 1.02 | 0.99 – 1.05 | .214 |
| BMI |  | 1.08 | 1.01 – 1.16 | **.021** |
| ln\_tsh |  | 0.48 | 0.27 – 0.81 | **.008** |
| ln\_ft4 |  | 0.61 | 0.05 – 6.72 | .690 |
| ln\_creat\_gPERlt\_spot |  | 1.19 | 0.79 – 1.79 | .407 |
| ln\_ClxBPA\_ngPERlt\_spot |  | 0.24 | 0.02 – 2.52 | .242 |
| Observations |  | 179 | | |

(B1) Adjusted for: age, BMI, TSH, FT4, spot iodine (ΞΌg/L) - table S8 - ClxBPA

|  |  |  |  |  |
| --- | --- | --- | --- | --- |
|  |  | status01 | | |
|  |  | OR | CI | p |
| (Intercept) |  | 4.81 | 0.00 – 1018480.46 | .800 |
| age |  | 1.02 | 0.99 – 1.05 | .215 |
| BMI |  | 1.09 | 1.02 – 1.17 | **.014** |
| ln\_tsh |  | 0.52 | 0.30 – 0.88 | **.017** |
| ln\_ft4 |  | 1.06 | 0.12 – 9.42 | .955 |
| ln\_iod\_ugPERlt\_spot |  | 0.68 | 0.43 – 1.04 | .082 |
| ln\_creat\_gPERlt\_spot |  | 1.73 | 0.97 – 3.19 | .068 |
| ln\_ClxBPA\_ngPERlt\_spot |  | 0.59 | 0.06 – 5.26 | .635 |
| Observations |  | 179 | | |

(B2) Adjusted for: study site age, BMI, TSH, FT4, spot iodine (ΞΌg/L) - table S8 - ClxBPA

|  |  |  |  |  |
| --- | --- | --- | --- | --- |
|  |  | status01 | | |
|  |  | OR | CI | p |
| (Intercept) |  | 1604.53 | 0.00 – 3418234838.35 | .310 |
| country (RO) |  | 1.92 | 0.88 – 4.32 | .108 |
| age |  | 1.02 | 0.99 – 1.06 | .129 |
| BMI |  | 1.08 | 1.01 – 1.16 | **.027** |
| ln\_tsh |  | 0.51 | 0.29 – 0.86 | **.014** |
| ln\_ft4 |  | 0.44 | 0.04 – 5.03 | .509 |
| ln\_iod\_ugPERlt\_spot |  | 0.65 | 0.41 – 1.00 | .057 |
| ln\_creat\_gPERlt\_spot |  | 1.79 | 1.00 – 3.33 | .055 |
| ln\_ClxBPA\_ngPERlt\_spot |  | 0.29 | 0.02 – 3.07 | .308 |
| Observations |  | 179 | | |

# 5 Determinants of exposure by compound in the whole study population

### 5.0.1 Table 2

**Determinants of BPA exposure - linear regression analysis (table 2 - BPA - model 1**

|  | | | | | | | | |
|  | Pooled population - questionnaire exposures | | | | | | | |
|  |  | | | | | | | |
|  | Spot BPA (ng/L creatinine, log transformed) | | | | | | | |
|  | (1) | (2) | (3) | (4) | (5) | (6) | (7) | (8) |
|  | | | | | | | | |
| ln\_creat\_gPERlt\_spot | 0.593 (0.413, 0.774) | 0.606 (0.423, 0.790) | 0.566 (0.377, 0.755) | 0.566 (0.368, 0.763) | 0.584 (0.383, 0.785) | 0.591 (0.407, 0.776) | 0.590 (0.404, 0.777) | 0.597 (0.410, 0.784) |
|  | p = 0.000 | p = 0.000 | p = 0.00000 | p = 0.00000 | p = 0.00000 | p = 0.000 | p = 0.000 | p = 0.000 |
| daily20lglass | 0.038 (-0.036, 0.111) |  |  |  |  |  |  |  |
|  | p = 0.315 |  |  |  |  |  |  |  |
| MwaveFrw |  | -0.049 (-0.125, 0.027) |  |  |  |  |  |  |
|  |  | p = 0.208 |  |  |  |  |  |  |
| weekPORTIONScanned |  |  | -0.015 (-0.070, 0.039) |  |  |  |  |  |
|  |  |  | p = 0.579 |  |  |  |  |  |
| weekMINScleaning |  |  |  | -0.001 (-0.002, -0.001) |  |  |  |  |
|  |  |  |  | p = 0.0004 |  |  |  |  |
| weekPORTIONSpcps |  |  |  |  | -0.003 (-0.007, 0.002) |  |  |  |
|  |  |  |  |  | p = 0.219 |  |  |  |
| perfwc |  |  |  |  |  | 0.006 (-0.036, 0.047) |  |  |
|  |  |  |  |  |  | p = 0.785 |  |  |
| weekPORTIONSdeo |  |  |  |  |  |  | 0.034 (-0.010, 0.078) |  |
|  |  |  |  |  |  |  | p = 0.128 |  |
| weekPORTIONScosm |  |  |  |  |  |  |  | 0.009 (-0.002, 0.020) |
|  |  |  |  |  |  |  |  | p = 0.129 |
| Constant | 7.936 (7.771, 8.102) | 8.008 (7.843, 8.172) | 7.969 (7.772, 8.166) | 8.321 (8.054, 8.587) | 8.134 (7.757, 8.511) | 7.926 (7.666, 8.186) | 7.789 (7.497, 8.081) | 7.857 (7.643, 8.071) |
|  | p = 0.000 | p = 0.000 | p = 0.000 | p = 0.000 | p = 0.000 | p = 0.000 | p = 0.000 | p = 0.000 |
|  | | | | | | | | |
| Observations | 203 | 202 | 188 | 179 | 164 | 201 | 194 | 196 |
| R2 | 0.177 | 0.175 | 0.163 | 0.203 | 0.170 | 0.167 | 0.173 | 0.177 |
| Adjusted R2 | 0.169 | 0.167 | 0.153 | 0.194 | 0.159 | 0.158 | 0.164 | 0.169 |
| Residual Std. Error | 1.061 (df = 200) | 1.062 (df = 199) | 1.070 (df = 185) | 1.067 (df = 176) | 1.059 (df = 161) | 1.065 (df = 198) | 1.063 (df = 191) | 1.073 (df = 193) |
| F Statistic | 21.568\*\*\* (df = 2; 200) | 21.093\*\*\* (df = 2; 199) | 17.950\*\*\* (df = 2; 185) | 22.354\*\*\* (df = 2; 176) | 16.461\*\*\* (df = 2; 161) | 19.780\*\*\* (df = 2; 198) | 19.911\*\*\* (df = 2; 191) | 20.805\*\*\* (df = 2; 193) |
|  | | | | | | | | |
| *Note:* | p<0.1; p<0.05; p<0.01 | | | | | | | |

**Determinants of BPA exposure - linear regression analysis (Table 2 - BPA - model 2**

|  | | | | | | | | |
|  | Pooled population - questionnaire exposures (adj. for study site, disease status, age, BMI | | | | | | | |
|  |  | | | | | | | |
|  | Spot BPA (ng/L, log transformed) | | | | | | | |
|  | (1) | (2) | (3) | (4) | (5) | (6) | (7) | (8) |
|  | | | | | | | | |
| ln\_creat\_gPERlt\_spot | 0.495 (0.330, 0.660) | 0.501 (0.333, 0.669) | 0.452 (0.275, 0.629) | 0.429 (0.247, 0.611) | 0.410 (0.216, 0.603) | 0.464 (0.292, 0.635) | 0.471 (0.296, 0.647) | 0.505 (0.332, 0.679) |
|  | p = 0.00000 | p = 0.00000 | p = 0.00001 | p = 0.00001 | p = 0.0001 | p = 0.00000 | p = 0.00000 | p = 0.00000 |
| countryRO | 1.056 (0.776, 1.336) | 1.009 (0.731, 1.286) | 0.999 (0.707, 1.290) | 1.104 (0.800, 1.409) | 1.235 (0.873, 1.598) | 1.026 (0.750, 1.303) | 1.004 (0.720, 1.288) | 1.019 (0.738, 1.301) |
|  | p = 0.000 | p = 0.000 | p = 0.000 | p = 0.000 | p = 0.000 | p = 0.000 | p = 0.000 | p = 0.000 |
| status011 | -0.389 (-0.656, -0.122) | -0.432 (-0.699, -0.164) | -0.410 (-0.697, -0.123) | -0.458 (-0.740, -0.176) | -0.428 (-0.725, -0.132) | -0.476 (-0.747, -0.206) | -0.475 (-0.751, -0.199) | -0.431 (-0.709, -0.153) |
|  | p = 0.005 | p = 0.002 | p = 0.006 | p = 0.002 | p = 0.006 | p = 0.001 | p = 0.001 | p = 0.003 |
| age | -0.006 (-0.018, 0.006) | -0.007 (-0.019, 0.006) | -0.009 (-0.021, 0.004) | -0.003 (-0.016, 0.009) | -0.005 (-0.019, 0.008) | -0.008 (-0.021, 0.004) | -0.004 (-0.017, 0.008) | -0.005 (-0.018, 0.008) |
|  | p = 0.320 | p = 0.292 | p = 0.188 | p = 0.608 | p = 0.433 | p = 0.195 | p = 0.503 | p = 0.473 |
| BMI | -0.002 (-0.029, 0.025) | 0.002 (-0.025, 0.029) | 0.003 (-0.027, 0.033) | -0.006 (-0.035, 0.022) | -0.001 (-0.032, 0.030) | -0.002 (-0.029, 0.025) | -0.002 (-0.030, 0.026) | 0.004 (-0.025, 0.032) |
|  | p = 0.903 | p = 0.886 | p = 0.854 | p = 0.675 | p = 0.958 | p = 0.894 | p = 0.883 | p = 0.807 |
| daily20lglass | 0.075 (0.002, 0.147) |  |  |  |  |  |  |  |
|  | p = 0.046 |  |  |  |  |  |  |  |
| MwaveFrw |  | -0.031 (-0.101, 0.039) |  |  |  |  |  |  |
|  |  | p = 0.386 |  |  |  |  |  |  |
| weekPORTIONScanned |  |  | -0.013 (-0.063, 0.036) |  |  |  |  |  |
|  |  |  | p = 0.598 |  |  |  |  |  |
| weekMINScleaning |  |  |  | -0.001 (-0.001, 0.0001) |  |  |  |  |
|  |  |  |  | p = 0.098 |  |  |  |  |
| weekPORTIONSpcps |  |  |  |  | 0.005 (0.001, 0.010) |  |  |  |
|  |  |  |  |  | p = 0.030 |  |  |  |
| perfwc |  |  |  |  |  | -0.024 (-0.064, 0.015) |  |  |
|  |  |  |  |  |  | p = 0.226 |  |  |
| weekPORTIONSdeo |  |  |  |  |  |  | 0.013 (-0.027, 0.053) |  |
|  |  |  |  |  |  |  | p = 0.532 |  |
| weekPORTIONScosm |  |  |  |  |  |  |  | 0.003 (-0.008, 0.014) |
|  |  |  |  |  |  |  |  | p = 0.599 |
| Constant | 7.978 (7.281, 8.674) | 8.036 (7.336, 8.736) | 8.099 (7.328, 8.871) | 8.203 (7.451, 8.955) | 7.523 (6.557, 8.490) | 8.326 (7.501, 9.150) | 7.961 (7.146, 8.777) | 7.852 (6.971, 8.732) |
|  | p = 0.000 | p = 0.000 | p = 0.000 | p = 0.000 | p = 0.000 | p = 0.000 | p = 0.000 | p = 0.000 |
|  | | | | | | | | |
| Observations | 198 | 197 | 183 | 175 | 159 | 196 | 189 | 191 |
| R2 | 0.401 | 0.394 | 0.377 | 0.418 | 0.386 | 0.391 | 0.381 | 0.388 |
| Adjusted R2 | 0.382 | 0.375 | 0.356 | 0.397 | 0.362 | 0.372 | 0.361 | 0.368 |
| Residual Std. Error | 0.922 (df = 191) | 0.927 (df = 190) | 0.941 (df = 176) | 0.927 (df = 168) | 0.931 (df = 152) | 0.927 (df = 189) | 0.937 (df = 182) | 0.943 (df = 184) |
| F Statistic | 21.289\*\*\* (df = 6; 191) | 20.600\*\*\* (df = 6; 190) | 17.739\*\*\* (df = 6; 176) | 20.103\*\*\* (df = 6; 168) | 15.926\*\*\* (df = 6; 152) | 20.246\*\*\* (df = 6; 189) | 18.689\*\*\* (df = 6; 182) | 19.402\*\*\* (df = 6; 184) |
|  | | | | | | | | |
| *Note:* | p<0.1; p<0.05; p<0.01 | | | | | | | |

## 5.1 Table S5

**5to95 - Determinants of BPA exposure - linear regression analysis - Table S5 - BPA**

|  | | | | | | | | |
|  | Pooled population - questionnaire exposures (adj. for study site, disease status, age, BMI | | | | | | | |
|  |  | | | | | | | |
|  | Spot BPA (ng/L, log transformed) | | | | | | | |
|  | (1) | (2) | (3) | (4) | (5) | (6) | (7) | (8) |
|  | | | | | | | | |
| ln\_creat\_gPERlt\_spot | 0.467 (0.334, 0.600) | 0.468 (0.333, 0.603) | 0.437 (0.295, 0.579) | 0.401 (0.255, 0.547) | 0.396 (0.252, 0.541) | 0.451 (0.312, 0.590) | 0.450 (0.308, 0.591) | 0.478 (0.338, 0.617) |
|  | p = 0.000 | p = 0.000 | p = 0.000 | p = 0.00000 | p = 0.00000 | p = 0.000 | p = 0.000 | p = 0.000 |
| countryRO | 0.681 (0.450, 0.912) | 0.644 (0.416, 0.872) | 0.619 (0.382, 0.856) | 0.707 (0.460, 0.955) | 0.712 (0.433, 0.992) | 0.639 (0.410, 0.868) | 0.619 (0.386, 0.853) | 0.637 (0.407, 0.868) |
|  | p = 0.00000 | p = 0.00000 | p = 0.00000 | p = 0.00000 | p = 0.00001 | p = 0.00000 | p = 0.00000 | p = 0.00000 |
| status011 | -0.185 (-0.404, 0.033) | -0.220 (-0.440, 0.0002) | -0.228 (-0.461, 0.005) | -0.258 (-0.488, -0.029) | -0.186 (-0.412, 0.039) | -0.242 (-0.467, -0.017) | -0.230 (-0.458, -0.003) | -0.205 (-0.434, 0.025) |
|  | p = 0.099 | p = 0.052 | p = 0.058 | p = 0.029 | p = 0.108 | p = 0.037 | p = 0.050 | p = 0.082 |
| age | -0.002 (-0.012, 0.008) | -0.002 (-0.012, 0.008) | -0.002 (-0.013, 0.008) | -0.00002 (-0.010, 0.010) | 0.001 (-0.010, 0.011) | -0.002 (-0.013, 0.008) | -0.001 (-0.011, 0.010) | 0.001 (-0.010, 0.012) |
|  | p = 0.670 | p = 0.670 | p = 0.655 | p = 0.998 | p = 0.906 | p = 0.668 | p = 0.881 | p = 0.860 |
| BMI | -0.001 (-0.023, 0.021) | 0.002 (-0.020, 0.024) | 0.007 (-0.017, 0.030) | -0.005 (-0.028, 0.018) | 0.002 (-0.021, 0.025) | 0.001 (-0.021, 0.023) | 0.00005 (-0.022, 0.022) | 0.005 (-0.018, 0.027) |
|  | p = 0.910 | p = 0.838 | p = 0.587 | p = 0.671 | p = 0.890 | p = 0.956 | p = 0.997 | p = 0.694 |
| daily20lglass | 0.056 (-0.002, 0.114) |  |  |  |  |  |  |  |
|  | p = 0.062 |  |  |  |  |  |  |  |
| MwaveFrw |  | -0.022 (-0.077, 0.032) |  |  |  |  |  |  |
|  |  | p = 0.426 |  |  |  |  |  |  |
| weekPORTIONScanned |  |  | -0.004 (-0.043, 0.035) |  |  |  |  |  |
|  |  |  | p = 0.844 |  |  |  |  |  |
| weekMINScleaning |  |  |  | -0.0003 (-0.001, 0.0002) |  |  |  |  |
|  |  |  |  | p = 0.295 |  |  |  |  |
| weekPORTIONSpcps |  |  |  |  | 0.004 (0.001, 0.008) |  |  |  |
|  |  |  |  |  | p = 0.020 |  |  |  |
| perfwc |  |  |  |  |  | -0.006 (-0.038, 0.026) |  |  |
|  |  |  |  |  |  | p = 0.708 |  |  |
| weekPORTIONSdeo |  |  |  |  |  |  | 0.008 (-0.025, 0.040) |  |
|  |  |  |  |  |  |  | p = 0.634 |  |
| weekPORTIONScosm |  |  |  |  |  |  |  | 0.007 (-0.003, 0.016) |
|  |  |  |  |  |  |  |  | p = 0.164 |
| Constant | 7.779 (7.218, 8.339) | 7.793 (7.228, 8.359) | 7.672 (7.054, 8.290) | 7.909 (7.306, 8.513) | 7.234 (6.519, 7.950) | 7.853 (7.191, 8.515) | 7.733 (7.071, 8.395) | 7.473 (6.779, 8.167) |
|  | p = 0.000 | p = 0.000 | p = 0.000 | p = 0.000 | p = 0.000 | p = 0.000 | p = 0.000 | p = 0.000 |
|  | | | | | | | | |
| Observations | 177 | 176 | 164 | 154 | 140 | 175 | 169 | 170 |
| R2 | 0.373 | 0.365 | 0.341 | 0.366 | 0.362 | 0.353 | 0.343 | 0.360 |
| Adjusted R2 | 0.351 | 0.342 | 0.316 | 0.340 | 0.334 | 0.330 | 0.319 | 0.336 |
| Residual Std. Error | 0.712 (df = 170) | 0.716 (df = 169) | 0.722 (df = 157) | 0.704 (df = 147) | 0.653 (df = 133) | 0.718 (df = 168) | 0.723 (df = 162) | 0.726 (df = 163) |
| F Statistic | 16.883\*\*\* (df = 6; 170) | 16.166\*\*\* (df = 6; 169) | 13.530\*\*\* (df = 6; 157) | 14.118\*\*\* (df = 6; 147) | 12.595\*\*\* (df = 6; 133) | 15.252\*\*\* (df = 6; 168) | 14.118\*\*\* (df = 6; 162) | 15.260\*\*\* (df = 6; 163) |
|  | | | | | | | | |
| *Note:* | p<0.1; p<0.05; p<0.01 | | | | | | | |

**Determinants of BPF exposure - linear regression analysis (Table 2 - BPF - model 1)**

|  | | | | | | | | |
|  | Pooled population - questionnaire exposures | | | | | | | |
|  |  | | | | | | | |
|  | Spot BPF (ng/L creatinine, log transformed) | | | | | | | |
|  | (1) | (2) | (3) | (4) | (5) | (6) | (7) | (8) |
|  | | | | | | | | |
| ln\_creat\_gPERlt\_spot | 0.451 (0.271, 0.632) | 0.401 (0.223, 0.579) | 0.386 (0.201, 0.571) | 0.519 (0.331, 0.707) | 0.408 (0.213, 0.602) | 0.431 (0.248, 0.614) | 0.429 (0.241, 0.617) | 0.464 (0.278, 0.650) |
|  | p = 0.00001 | p = 0.00002 | p = 0.0001 | p = 0.00000 | p = 0.0001 | p = 0.00001 | p = 0.00002 | p = 0.00001 |
| daily20lglass | 0.005 (-0.068, 0.078) |  |  |  |  |  |  |  |
|  | p = 0.886 |  |  |  |  |  |  |  |
| MwaveFrw |  | 0.124 (0.050, 0.198) |  |  |  |  |  |  |
|  |  | p = 0.002 |  |  |  |  |  |  |
| weekPORTIONScanned |  |  | -0.006 (-0.059, 0.047) |  |  |  |  |  |
|  |  |  | p = 0.821 |  |  |  |  |  |
| weekMINScleaning |  |  |  | 0.00001 (-0.001, 0.001) |  |  |  |  |
|  |  |  |  | p = 0.966 |  |  |  |  |
| weekPORTIONSpcps |  |  |  |  | 0.002 (-0.002, 0.006) |  |  |  |
|  |  |  |  |  | p = 0.375 |  |  |  |
| perfwc |  |  |  |  |  | -0.014 (-0.055, 0.027) |  |  |
|  |  |  |  |  |  | p = 0.502 |  |  |
| weekPORTIONSdeo |  |  |  |  |  |  | 0.006 (-0.039, 0.050) |  |
|  |  |  |  |  |  |  | p = 0.806 |  |
| weekPORTIONScosm |  |  |  |  |  |  |  | -0.0001 (-0.011, 0.011) |
|  |  |  |  |  |  |  |  | p = 0.983 |
| Constant | 6.414 (6.248, 6.579) | 6.342 (6.182, 6.502) | 6.411 (6.218, 6.604) | 6.441 (6.187, 6.696) | 6.294 (5.929, 6.659) | 6.505 (6.248, 6.762) | 6.416 (6.122, 6.710) | 6.451 (6.239, 6.663) |
|  | p = 0.000 | p = 0.000 | p = 0.000 | p = 0.000 | p = 0.000 | p = 0.000 | p = 0.000 | p = 0.000 |
|  | | | | | | | | |
| Observations | 203 | 202 | 188 | 179 | 164 | 201 | 194 | 196 |
| R2 | 0.108 | 0.151 | 0.085 | 0.143 | 0.103 | 0.098 | 0.095 | 0.110 |
| Adjusted R2 | 0.099 | 0.142 | 0.075 | 0.133 | 0.092 | 0.089 | 0.085 | 0.101 |
| Residual Std. Error | 1.060 (df = 200) | 1.032 (df = 199) | 1.045 (df = 185) | 1.018 (df = 176) | 1.023 (df = 161) | 1.055 (df = 198) | 1.071 (df = 191) | 1.064 (df = 193) |
| F Statistic | 12.064\*\*\* (df = 2; 200) | 17.672\*\*\* (df = 2; 199) | 8.622\*\*\* (df = 2; 185) | 14.624\*\*\* (df = 2; 176) | 9.241\*\*\* (df = 2; 161) | 10.800\*\*\* (df = 2; 198) | 9.975\*\*\* (df = 2; 191) | 11.966\*\*\* (df = 2; 193) |
|  | | | | | | | | |
| *Note:* | p<0.1; p<0.05; p<0.01 | | | | | | | |

**Determinants of BPF exposure - linear regression analysis (Table 2 - BPF - model 2)**

|  | | | | | | | | |
|  | Pooled population - questionnaire exposures (adj. for study site, disease status, age, BMI | | | | | | | |
|  |  | | | | | | | |
|  | Spot BPF (ng/L, log transformed) | | | | | | | |
|  | (1) | (2) | (3) | (4) | (5) | (6) | (7) | (8) |
|  | | | | | | | | |
| ln\_creat\_gPERlt\_spot | 0.448 (0.255, 0.642) | 0.400 (0.210, 0.590) | 0.380 (0.179, 0.581) | 0.494 (0.291, 0.697) | 0.385 (0.167, 0.603) | 0.411 (0.212, 0.610) | 0.422 (0.217, 0.626) | 0.454 (0.254, 0.654) |
|  | p = 0.00001 | p = 0.0001 | p = 0.0003 | p = 0.00001 | p = 0.001 | p = 0.0001 | p = 0.0001 | p = 0.00002 |
| countryRO | -0.073 (-0.401, 0.255) | 0.024 (-0.290, 0.338) | -0.102 (-0.434, 0.229) | -0.035 (-0.374, 0.304) | 0.036 (-0.372, 0.445) | -0.013 (-0.333, 0.308) | -0.043 (-0.373, 0.288) | -0.041 (-0.366, 0.283) |
|  | p = 0.662 | p = 0.882 | p = 0.545 | p = 0.840 | p = 0.863 | p = 0.938 | p = 0.800 | p = 0.804 |
| status011 | -0.025 (-0.338, 0.288) | -0.051 (-0.353, 0.252) | -0.043 (-0.369, 0.283) | -0.090 (-0.404, 0.224) | 0.015 (-0.319, 0.349) | -0.097 (-0.411, 0.216) | -0.068 (-0.389, 0.254) | -0.035 (-0.356, 0.286) |
|  | p = 0.876 | p = 0.743 | p = 0.798 | p = 0.577 | p = 0.932 | p = 0.544 | p = 0.680 | p = 0.830 |
| age | -0.00000 (-0.014, 0.014) | 0.006 (-0.008, 0.020) | 0.0003 (-0.014, 0.015) | -0.002 (-0.017, 0.012) | 0.001 (-0.014, 0.016) | -0.001 (-0.016, 0.013) | 0.001 (-0.014, 0.016) | -0.002 (-0.018, 0.013) |
|  | p = 1.000 | p = 0.410 | p = 0.967 | p = 0.759 | p = 0.910 | p = 0.847 | p = 0.888 | p = 0.761 |
| BMI | -0.011 (-0.043, 0.021) | -0.018 (-0.049, 0.012) | -0.011 (-0.045, 0.023) | -0.019 (-0.051, 0.013) | -0.021 (-0.056, 0.014) | -0.016 (-0.047, 0.016) | -0.017 (-0.050, 0.015) | -0.007 (-0.039, 0.026) |
|  | p = 0.489 | p = 0.242 | p = 0.530 | p = 0.244 | p = 0.247 | p = 0.329 | p = 0.293 | p = 0.694 |
| daily20lglass | 0.001 (-0.085, 0.086) |  |  |  |  |  |  |  |
|  | p = 0.989 |  |  |  |  |  |  |  |
| MwaveFrw |  | 0.142 (0.062, 0.221) |  |  |  |  |  |  |
|  |  | p = 0.001 |  |  |  |  |  |  |
| weekPORTIONScanned |  |  | -0.008 (-0.065, 0.048) |  |  |  |  |  |
|  |  |  | p = 0.776 |  |  |  |  |  |
| weekMINScleaning |  |  |  | 0.0001 (-0.001, 0.001) |  |  |  |  |
|  |  |  |  | p = 0.821 |  |  |  |  |
| weekPORTIONSpcps |  |  |  |  | 0.002 (-0.004, 0.007) |  |  |  |
|  |  |  |  |  | p = 0.542 |  |  |  |
| perfwc |  |  |  |  |  | -0.024 (-0.069, 0.022) |  |  |
|  |  |  |  |  |  | p = 0.310 |  |  |
| weekPORTIONSdeo |  |  |  |  |  |  | 0.004 (-0.043, 0.052) |  |
|  |  |  |  |  |  |  | p = 0.854 |  |
| weekPORTIONScosm |  |  |  |  |  |  |  | -0.002 (-0.015, 0.011) |
|  |  |  |  |  |  |  |  | p = 0.752 |
| Constant | 6.751 (5.934, 7.569) | 6.540 (5.748, 7.333) | 6.738 (5.863, 7.614) | 7.066 (6.228, 7.904) | 6.780 (5.692, 7.867) | 7.080 (6.126, 8.035) | 6.873 (5.923, 7.823) | 6.796 (5.781, 7.811) |
|  | p = 0.000 | p = 0.000 | p = 0.000 | p = 0.000 | p = 0.000 | p = 0.000 | p = 0.000 | p = 0.000 |
|  | | | | | | | | |
| Observations | 198 | 197 | 183 | 175 | 159 | 196 | 189 | 191 |
| R2 | 0.109 | 0.160 | 0.088 | 0.154 | 0.108 | 0.105 | 0.101 | 0.110 |
| Adjusted R2 | 0.081 | 0.134 | 0.056 | 0.124 | 0.073 | 0.077 | 0.071 | 0.081 |
| Residual Std. Error | 1.081 (df = 191) | 1.049 (df = 190) | 1.067 (df = 176) | 1.033 (df = 168) | 1.048 (df = 152) | 1.073 (df = 189) | 1.091 (df = 182) | 1.087 (df = 184) |
| F Statistic | 3.906\*\*\* (df = 6; 191) | 6.042\*\*\* (df = 6; 190) | 2.814\*\* (df = 6; 176) | 5.093\*\*\* (df = 6; 168) | 3.080\*\*\* (df = 6; 152) | 3.711\*\*\* (df = 6; 189) | 3.400\*\*\* (df = 6; 182) | 3.791\*\*\* (df = 6; 184) |
|  | | | | | | | | |
| *Note:* | p<0.1; p<0.05; p<0.01 | | | | | | | |

**5to95 - Determinants of BPF exposure - linear regression analysis - Table S5 - BPF**

|  | | | | | | | | |
|  | Pooled population - questionnaire exposures (adj. for study site, disease status, age, BMI | | | | | | | |
|  |  | | | | | | | |
|  | Spot BPF (ng/L, log transformed) | | | | | | | |
|  | (1) | (2) | (3) | (4) | (5) | (6) | (7) | (8) |
|  | | | | | | | | |
| ln\_creat\_gPERlt\_spot | 0.240 (0.118, 0.363) | 0.232 (0.109, 0.354) | 0.225 (0.106, 0.345) | 0.259 (0.140, 0.379) | 0.226 (0.095, 0.357) | 0.235 (0.112, 0.357) | 0.240 (0.114, 0.366) | 0.248 (0.121, 0.375) |
|  | p = 0.0002 | p = 0.0003 | p = 0.0003 | p = 0.00004 | p = 0.001 | p = 0.0003 | p = 0.0003 | p = 0.0002 |
| countryRO | -0.055 (-0.260, 0.149) | -0.040 (-0.241, 0.160) | -0.105 (-0.304, 0.095) | 0.018 (-0.188, 0.224) | 0.090 (-0.156, 0.336) | -0.037 (-0.237, 0.164) | -0.063 (-0.269, 0.142) | -0.055 (-0.257, 0.148) |
|  | p = 0.596 | p = 0.694 | p = 0.306 | p = 0.864 | p = 0.477 | p = 0.720 | p = 0.547 | p = 0.600 |
| status011 | 0.153 (-0.032, 0.338) | 0.110 (-0.074, 0.294) | 0.114 (-0.072, 0.300) | 0.163 (-0.019, 0.346) | 0.097 (-0.099, 0.292) | 0.087 (-0.099, 0.272) | 0.105 (-0.085, 0.295) | 0.110 (-0.080, 0.301) |
|  | p = 0.108 | p = 0.244 | p = 0.231 | p = 0.082 | p = 0.333 | p = 0.362 | p = 0.282 | p = 0.259 |
| age | -0.002 (-0.011, 0.006) | -0.001 (-0.009, 0.008) | -0.001 (-0.009, 0.007) | -0.002 (-0.010, 0.007) | -0.0001 (-0.009, 0.009) | -0.003 (-0.011, 0.006) | -0.001 (-0.010, 0.008) | -0.005 (-0.014, 0.005) |
|  | p = 0.596 | p = 0.894 | p = 0.803 | p = 0.714 | p = 0.986 | p = 0.576 | p = 0.826 | p = 0.331 |
| BMI | -0.007 (-0.027, 0.013) | -0.006 (-0.026, 0.014) | -0.011 (-0.032, 0.009) | -0.003 (-0.023, 0.016) | -0.012 (-0.034, 0.009) | -0.006 (-0.026, 0.013) | -0.008 (-0.028, 0.012) | -0.003 (-0.023, 0.017) |
|  | p = 0.500 | p = 0.559 | p = 0.283 | p = 0.744 | p = 0.269 | p = 0.533 | p = 0.411 | p = 0.775 |
| daily20lglass | 0.022 (-0.027, 0.071) |  |  |  |  |  |  |  |
|  | p = 0.375 |  |  |  |  |  |  |  |
| MwaveFrw |  | 0.056 (-0.003, 0.115) |  |  |  |  |  |  |
|  |  | p = 0.067 |  |  |  |  |  |  |
| weekPORTIONScanned |  |  | 0.017 (-0.015, 0.049) |  |  |  |  |  |
|  |  |  | p = 0.305 |  |  |  |  |  |
| weekMINScleaning |  |  |  | -0.00005 (-0.0005, 0.0004) |  |  |  |  |
|  |  |  |  | p = 0.824 |  |  |  |  |
| weekPORTIONSpcps |  |  |  |  | 0.003 (-0.0003, 0.006) |  |  |  |
|  |  |  |  |  | p = 0.079 |  |  |  |
| perfwc |  |  |  |  |  | -0.012 (-0.039, 0.014) |  |  |
|  |  |  |  |  |  | p = 0.369 |  |  |
| weekPORTIONSdeo |  |  |  |  |  |  | 0.006 (-0.022, 0.035) |  |
|  |  |  |  |  |  |  | p = 0.672 |  |
| weekPORTIONScosm |  |  |  |  |  |  |  | -0.004 (-0.012, 0.004) |
|  |  |  |  |  |  |  |  | p = 0.344 |
| Constant | 6.469 (5.959, 6.979) | 6.377 (5.869, 6.885) | 6.523 (6.002, 7.044) | 6.372 (5.873, 6.870) | 6.301 (5.649, 6.953) | 6.588 (6.018, 7.157) | 6.484 (5.921, 7.047) | 6.597 (5.947, 7.247) |
|  | p = 0.000 | p = 0.000 | p = 0.000 | p = 0.000 | p = 0.000 | p = 0.000 | p = 0.000 | p = 0.000 |
|  | | | | | | | | |
| Observations | 177 | 176 | 165 | 158 | 144 | 176 | 169 | 171 |
| R2 | 0.119 | 0.120 | 0.111 | 0.147 | 0.146 | 0.108 | 0.103 | 0.118 |
| Adjusted R2 | 0.088 | 0.089 | 0.077 | 0.113 | 0.109 | 0.077 | 0.070 | 0.086 |
| Residual Std. Error | 0.611 (df = 170) | 0.608 (df = 169) | 0.584 (df = 158) | 0.571 (df = 151) | 0.586 (df = 137) | 0.607 (df = 169) | 0.614 (df = 162) | 0.614 (df = 164) |
| F Statistic | 3.833\*\*\* (df = 6; 170) | 3.852\*\*\* (df = 6; 169) | 3.280\*\*\* (df = 6; 158) | 4.326\*\*\* (df = 6; 151) | 3.911\*\*\* (df = 6; 137) | 3.425\*\*\* (df = 6; 169) | 3.108\*\*\* (df = 6; 162) | 3.668\*\*\* (df = 6; 164) |
|  | | | | | | | | |
| *Note:* | p<0.1; p<0.05; p<0.01 | | | | | | | |

**Determinants of ClxBPA exposure - linear regression analysis (Table 2 - ClxBPA - model 1)**

|  | | | | | | | | |
|  | Pooled population - questionnaire exposures | | | | | | | |
|  |  | | | | | | | |
|  | Spot ClxBPA (ng/L creatinine, log transformed) | | | | | | | |
|  | (1) | (2) | (3) | (4) | (5) | (6) | (7) | (8) |
|  | | | | | | | | |
| ln\_creat\_gPERlt\_spot | 0.053 (0.015, 0.090) | 0.057 (0.020, 0.094) | 0.052 (0.012, 0.092) | 0.053 (0.010, 0.095) | 0.055 (0.012, 0.099) | 0.052 (0.014, 0.090) | 0.053 (0.014, 0.092) | 0.054 (0.015, 0.093) |
|  | p = 0.007 | p = 0.003 | p = 0.013 | p = 0.016 | p = 0.014 | p = 0.009 | p = 0.009 | p = 0.007 |
| daily20lglass | 0.006 (-0.009, 0.021) |  |  |  |  |  |  |  |
|  | p = 0.461 |  |  |  |  |  |  |  |
| MwaveFrw |  | -0.021 (-0.036, -0.005) |  |  |  |  |  |  |
|  |  | p = 0.009 |  |  |  |  |  |  |
| weekPORTIONScanned |  |  | -0.002 (-0.014, 0.009) |  |  |  |  |  |
|  |  |  | p = 0.700 |  |  |  |  |  |
| weekMINScleaning |  |  |  | -0.00003 (-0.0002, 0.0001) |  |  |  |  |
|  |  |  |  | p = 0.717 |  |  |  |  |
| weekPORTIONSpcps |  |  |  |  | -0.0002 (-0.001, 0.001) |  |  |  |
|  |  |  |  |  | p = 0.622 |  |  |  |
| perfwc |  |  |  |  |  | 0.003 (-0.005, 0.012) |  |  |
|  |  |  |  |  |  | p = 0.462 |  |  |
| weekPORTIONSdeo |  |  |  |  |  |  | 0.001 (-0.008, 0.010) |  |
|  |  |  |  |  |  |  | p = 0.851 |  |
| weekPORTIONScosm |  |  |  |  |  |  |  | 0.002 (0.0001, 0.005) |
|  |  |  |  |  |  |  |  | p = 0.046 |
| Constant | 5.143 (5.109, 5.177) | 5.156 (5.123, 5.189) | 5.150 (5.108, 5.192) | 5.155 (5.098, 5.213) | 5.166 (5.084, 5.248) | 5.127 (5.073, 5.181) | 5.143 (5.082, 5.205) | 5.116 (5.072, 5.160) |
|  | p = 0.000 | p = 0.000 | p = 0.000 | p = 0.000 | p = 0.000 | p = 0.000 | p = 0.000 | p = 0.000 |
|  | | | | | | | | |
| Observations | 203 | 202 | 188 | 179 | 164 | 201 | 194 | 196 |
| R2 | 0.041 | 0.066 | 0.036 | 0.033 | 0.037 | 0.038 | 0.036 | 0.057 |
| Adjusted R2 | 0.031 | 0.057 | 0.025 | 0.022 | 0.025 | 0.028 | 0.026 | 0.048 |
| Residual Std. Error | 0.219 (df = 200) | 0.213 (df = 199) | 0.227 (df = 185) | 0.230 (df = 176) | 0.230 (df = 161) | 0.221 (df = 198) | 0.223 (df = 191) | 0.221 (df = 193) |
| F Statistic | 4.225\*\* (df = 2; 200) | 7.063\*\*\* (df = 2; 199) | 3.407\*\* (df = 2; 185) | 3.043\* (df = 2; 176) | 3.128\*\* (df = 2; 161) | 3.875\*\* (df = 2; 198) | 3.547\*\* (df = 2; 191) | 5.877\*\*\* (df = 2; 193) |
|  | | | | | | | | |
| *Note:* | p<0.1; p<0.05; p<0.01 | | | | | | | |

**Determinants of ClxBPA exposure - linear regression analysis (Table - ClxBPA - model 2)**

|  | | | | | | | | |
|  | Pooled population - questionnaire exposures (adj. for study site, disease status, age, BMI | | | | | | | |
|  |  | | | | | | | |
|  | Spot ClxBPA (ng/L, log transformed) | | | | | | | |
|  | (1) | (2) | (3) | (4) | (5) | (6) | (7) | (8) |
|  | | | | | | | | |
| ln\_creat\_gPERlt\_spot | 0.037 (0.001, 0.074) | 0.039 (0.002, 0.075) | 0.037 (-0.003, 0.077) | 0.032 (-0.010, 0.074) | 0.028 (-0.016, 0.073) | 0.036 (-0.003, 0.074) | 0.036 (-0.004, 0.075) | 0.043 (0.005, 0.081) |
|  | p = 0.049 | p = 0.039 | p = 0.075 | p = 0.139 | p = 0.214 | p = 0.071 | p = 0.080 | p = 0.027 |
| countryRO | 0.137 (0.076, 0.199) | 0.125 (0.065, 0.185) | 0.130 (0.063, 0.196) | 0.130 (0.059, 0.200) | 0.167 (0.083, 0.250) | 0.126 (0.064, 0.188) | 0.122 (0.058, 0.186) | 0.130 (0.068, 0.191) |
|  | p = 0.00003 | p = 0.0001 | p = 0.0002 | p = 0.0005 | p = 0.0002 | p = 0.0001 | p = 0.0003 | p = 0.0001 |
| status011 | -0.018 (-0.077, 0.041) | -0.014 (-0.072, 0.044) | -0.017 (-0.082, 0.048) | -0.022 (-0.088, 0.043) | -0.018 (-0.086, 0.051) | -0.018 (-0.078, 0.043) | -0.017 (-0.079, 0.045) | -0.011 (-0.072, 0.050) |
|  | p = 0.554 | p = 0.634 | p = 0.604 | p = 0.504 | p = 0.614 | p = 0.563 | p = 0.583 | p = 0.723 |
| age | 0.00002 (-0.003, 0.003) | -0.001 (-0.003, 0.002) | -0.0003 (-0.003, 0.003) | -0.0002 (-0.003, 0.003) | -0.001 (-0.004, 0.003) | -0.00004 (-0.003, 0.003) | -0.0002 (-0.003, 0.003) | 0.001 (-0.002, 0.004) |
|  | p = 0.987 | p = 0.698 | p = 0.853 | p = 0.876 | p = 0.724 | p = 0.979 | p = 0.915 | p = 0.389 |
| BMI | 0.002 (-0.004, 0.008) | 0.003 (-0.003, 0.008) | 0.003 (-0.004, 0.010) | 0.003 (-0.004, 0.009) | 0.004 (-0.004, 0.011) | 0.002 (-0.004, 0.008) | 0.002 (-0.004, 0.008) | 0.003 (-0.003, 0.009) |
|  | p = 0.487 | p = 0.379 | p = 0.363 | p = 0.449 | p = 0.336 | p = 0.456 | p = 0.522 | p = 0.310 |
| daily20lglass | 0.016 (-0.0002, 0.032) |  |  |  |  |  |  |  |
|  | p = 0.055 |  |  |  |  |  |  |  |
| MwaveFrw |  | -0.016 (-0.031, -0.001) |  |  |  |  |  |  |
|  |  | p = 0.040 |  |  |  |  |  |  |
| weekPORTIONScanned |  |  | -0.001 (-0.012, 0.011) |  |  |  |  |  |
|  |  |  | p = 0.916 |  |  |  |  |  |
| weekMINScleaning |  |  |  | -0.00004 (-0.0002, 0.0001) |  |  |  |  |
|  |  |  |  | p = 0.615 |  |  |  |  |
| weekPORTIONSpcps |  |  |  |  | 0.001 (-0.0001, 0.002) |  |  |  |
|  |  |  |  |  | p = 0.071 |  |  |  |
| perfwc |  |  |  |  |  | 0.001 (-0.007, 0.010) |  |  |
|  |  |  |  |  |  | p = 0.741 |  |  |
| weekPORTIONSdeo |  |  |  |  |  |  | 0.0001 (-0.009, 0.009) |  |
|  |  |  |  |  |  |  | p = 0.988 |  |
| weekPORTIONScosm |  |  |  |  |  |  |  | 0.003 (0.001, 0.006) |
|  |  |  |  |  |  |  |  | p = 0.010 |
| Constant | 5.023 (4.868, 5.177) | 5.056 (4.905, 5.207) | 5.029 (4.854, 5.204) | 5.057 (4.883, 5.232) | 4.942 (4.719, 5.165) | 5.027 (4.843, 5.211) | 5.051 (4.868, 5.235) | 4.903 (4.710, 5.095) |
|  | p = 0.000 | p = 0.000 | p = 0.000 | p = 0.000 | p = 0.000 | p = 0.000 | p = 0.000 | p = 0.000 |
|  | | | | | | | | |
| Observations | 198 | 197 | 183 | 175 | 159 | 196 | 189 | 191 |
| R2 | 0.137 | 0.146 | 0.117 | 0.117 | 0.127 | 0.117 | 0.109 | 0.149 |
| Adjusted R2 | 0.110 | 0.119 | 0.087 | 0.086 | 0.092 | 0.089 | 0.079 | 0.121 |
| Residual Std. Error | 0.204 (df = 191) | 0.200 (df = 190) | 0.213 (df = 176) | 0.215 (df = 168) | 0.215 (df = 152) | 0.207 (df = 189) | 0.211 (df = 182) | 0.206 (df = 184) |
| F Statistic | 5.047\*\*\* (df = 6; 191) | 5.432\*\*\* (df = 6; 190) | 3.876\*\*\* (df = 6; 176) | 3.714\*\*\* (df = 6; 168) | 3.681\*\*\* (df = 6; 152) | 4.173\*\*\* (df = 6; 189) | 3.693\*\*\* (df = 6; 182) | 5.362\*\*\* (df = 6; 184) |
|  | | | | | | | | |
| *Note:* | p<0.1; p<0.05; p<0.01 | | | | | | | |

**5to95 - Determinants of ClxBPA exposure - linear regression analysis - Table S5 - ClxBPA**

|  | | | | | | | | |
|  | Pooled population - questionnaire exposures (adj. for study site, disease status, age, BMI | | | | | | | |
|  |  | | | | | | | |
|  | Spot ClxBPA (ng/L, log transformed) | | | | | | | |
|  | (1) | (2) | (3) | (4) | (5) | (6) | (7) | (8) |
|  | | | | | | | | |
| ln\_creat\_gPERlt\_spot | 0.026 (0.001, 0.051) | 0.030 (0.005, 0.056) | 0.024 (-0.004, 0.051) | 0.019 (-0.009, 0.048) | 0.024 (-0.005, 0.052) | 0.024 (-0.003, 0.050) | 0.026 (-0.001, 0.054) | 0.032 (0.005, 0.058) |
|  | p = 0.043 | p = 0.022 | p = 0.092 | p = 0.180 | p = 0.109 | p = 0.083 | p = 0.061 | p = 0.022 |
| countryRO | 0.123 (0.081, 0.166) | 0.106 (0.064, 0.148) | 0.107 (0.062, 0.153) | 0.117 (0.069, 0.164) | 0.106 (0.051, 0.161) | 0.111 (0.068, 0.153) | 0.105 (0.061, 0.149) | 0.112 (0.069, 0.155) |
|  | p = 0.00000 | p = 0.00001 | p = 0.00001 | p = 0.00001 | p = 0.0003 | p = 0.00000 | p = 0.00001 | p = 0.00000 |
| status011 | -0.020 (-0.061, 0.021) | -0.024 (-0.065, 0.017) | -0.027 (-0.072, 0.018) | -0.036 (-0.081, 0.008) | -0.014 (-0.059, 0.031) | -0.025 (-0.066, 0.017) | -0.021 (-0.064, 0.022) | -0.023 (-0.066, 0.020) |
|  | p = 0.335 | p = 0.253 | p = 0.242 | p = 0.112 | p = 0.540 | p = 0.253 | p = 0.336 | p = 0.296 |
| age | 0.0003 (-0.002, 0.002) | -0.0003 (-0.002, 0.002) | -0.0001 (-0.002, 0.002) | -0.0005 (-0.003, 0.002) | -0.00000 (-0.002, 0.002) | -0.0003 (-0.002, 0.002) | 0.0001 (-0.002, 0.002) | 0.001 (-0.001, 0.003) |
|  | p = 0.753 | p = 0.780 | p = 0.955 | p = 0.643 | p = 1.000 | p = 0.806 | p = 0.921 | p = 0.463 |
| BMI | -0.001 (-0.005, 0.003) | 0.0003 (-0.004, 0.004) | 0.0001 (-0.005, 0.005) | -0.001 (-0.005, 0.004) | -0.001 (-0.006, 0.003) | -0.0004 (-0.005, 0.004) | -0.001 (-0.005, 0.004) | -0.0001 (-0.004, 0.004) |
|  | p = 0.710 | p = 0.900 | p = 0.971 | p = 0.747 | p = 0.571 | p = 0.867 | p = 0.808 | p = 0.965 |
| daily20lglass | 0.013 (0.002, 0.024) |  |  |  |  |  |  |  |
|  | p = 0.017 |  |  |  |  |  |  |  |
| MwaveFrw |  | -0.010 (-0.021, 0.0004) |  |  |  |  |  |  |
|  |  | p = 0.062 |  |  |  |  |  |  |
| weekPORTIONScanned |  |  | -0.004 (-0.012, 0.004) |  |  |  |  |  |
|  |  |  | p = 0.307 |  |  |  |  |  |
| weekMINScleaning |  |  |  | 0.00005 (-0.0001, 0.0001) |  |  |  |  |
|  |  |  |  | p = 0.365 |  |  |  |  |
| weekPORTIONSpcps |  |  |  |  | 0.0001 (-0.001, 0.001) |  |  |  |
|  |  |  |  |  | p = 0.720 |  |  |  |
| perfwc |  |  |  |  |  | -0.002 (-0.008, 0.004) |  |  |
|  |  |  |  |  |  | p = 0.564 |  |  |
| weekPORTIONSdeo |  |  |  |  |  |  | 0.002 (-0.004, 0.008) |  |
|  |  |  |  |  |  |  | p = 0.538 |  |
| weekPORTIONScosm |  |  |  |  |  |  |  | 0.002 (-0.0002, 0.003) |
|  |  |  |  |  |  |  |  | p = 0.092 |
| Constant | 5.069 (4.964, 5.174) | 5.095 (4.989, 5.201) | 5.093 (4.973, 5.213) | 5.111 (4.994, 5.229) | 5.098 (4.948, 5.248) | 5.109 (4.979, 5.240) | 5.080 (4.953, 5.207) | 5.026 (4.890, 5.162) |
|  | p = 0.000 | p = 0.000 | p = 0.000 | p = 0.000 | p = 0.000 | p = 0.000 | p = 0.000 | p = 0.000 |
|  | | | | | | | | |
| Observations | 180 | 179 | 164 | 157 | 143 | 177 | 170 | 172 |
| R2 | 0.206 | 0.196 | 0.170 | 0.184 | 0.157 | 0.176 | 0.165 | 0.190 |
| Adjusted R2 | 0.179 | 0.168 | 0.138 | 0.151 | 0.120 | 0.147 | 0.134 | 0.161 |
| Residual Std. Error | 0.134 (df = 173) | 0.135 (df = 172) | 0.140 (df = 157) | 0.138 (df = 150) | 0.134 (df = 136) | 0.136 (df = 170) | 0.138 (df = 163) | 0.138 (df = 165) |
| F Statistic | 7.490\*\*\* (df = 6; 173) | 6.987\*\*\* (df = 6; 172) | 5.358\*\*\* (df = 6; 157) | 5.634\*\*\* (df = 6; 150) | 4.216\*\*\* (df = 6; 136) | 6.072\*\*\* (df = 6; 170) | 5.371\*\*\* (df = 6; 163) | 6.460\*\*\* (df = 6; 165) |
|  | | | | | | | | |
| *Note:* | p<0.1; p<0.05; p<0.01 | | | | | | | |

## 5.2 Table S10 (additional table)

**Determinants of BPA exposure - linear regression analysis - Table S10 (additional)**

|  | | | | | | | | |
|  | Pooled population (CASES only) - questionnaire exposures (adj. for study site, disease status, age, BMI | | | | | | | |
|  |  | | | | | | | |
|  | Spot BPA (ng/L, log transformed) | | | | | | | |
|  | (1) | (2) | (3) | (4) | (5) | (6) | (7) | (8) |
|  | | | | | | | | |
| ln\_creat\_gPERlt\_spot | 0.584 (0.374, 0.794) | 0.576 (0.358, 0.794) | 0.579 (0.352, 0.805) | 0.562 (0.337, 0.787) | 0.435 (0.206, 0.665) | 0.599 (0.382, 0.815) | 0.608 (0.387, 0.830) | 0.615 (0.395, 0.836) |
|  | p = 0.00000 | p = 0.00001 | p = 0.00001 | p = 0.00001 | p = 0.0004 | p = 0.00000 | p = 0.00000 | p = 0.00000 |
| countryRO | 0.493 (0.161, 0.824) | 0.444 (0.105, 0.782) | 0.364 (0.006, 0.722) | 0.344 (-0.026, 0.713) | 0.759 (0.329, 1.190) | 0.391 (0.055, 0.728) | 0.367 (0.020, 0.714) | 0.390 (0.048, 0.732) |
|  | p = 0.005 | p = 0.012 | p = 0.050 | p = 0.073 | p = 0.001 | p = 0.026 | p = 0.042 | p = 0.028 |
| age | -0.006 (-0.020, 0.009) | -0.009 (-0.024, 0.006) | -0.011 (-0.027, 0.005) | -0.002 (-0.018, 0.013) | -0.007 (-0.023, 0.008) | -0.007 (-0.022, 0.008) | -0.007 (-0.023, 0.008) | -0.006 (-0.023, 0.010) |
|  | p = 0.460 | p = 0.249 | p = 0.185 | p = 0.765 | p = 0.366 | p = 0.355 | p = 0.350 | p = 0.450 |
| BMI | 0.012 (-0.016, 0.041) | 0.011 (-0.019, 0.042) | 0.019 (-0.013, 0.052) | 0.006 (-0.024, 0.036) | 0.012 (-0.021, 0.044) | 0.013 (-0.016, 0.042) | 0.014 (-0.016, 0.043) | 0.018 (-0.013, 0.048) |
|  | p = 0.406 | p = 0.460 | p = 0.251 | p = 0.693 | p = 0.488 | p = 0.396 | p = 0.366 | p = 0.266 |
| daily20lglass | 0.143 (0.026, 0.261) |  |  |  |  |  |  |  |
|  | p = 0.019 |  |  |  |  |  |  |  |
| MwaveFrw |  | -0.004 (-0.101, 0.092) |  |  |  |  |  |  |
|  |  | p = 0.928 |  |  |  |  |  |  |
| weekPORTIONScanned |  |  | -0.026 (-0.100, 0.047) |  |  |  |  |  |
|  |  |  | p = 0.485 |  |  |  |  |  |
| weekMINScleaning |  |  |  | -0.001 (-0.002, -0.0002) |  |  |  |  |
|  |  |  |  | p = 0.017 |  |  |  |  |
| weekPORTIONSpcps |  |  |  |  | 0.008 (0.003, 0.013) |  |  |  |
|  |  |  |  |  | p = 0.003 |  |  |  |
| perfwc |  |  |  |  |  | 0.011 (-0.033, 0.055) |  |  |
|  |  |  |  |  |  | p = 0.638 |  |  |
| weekPORTIONSdeo |  |  |  |  |  |  | 0.015 (-0.028, 0.059) |  |
|  |  |  |  |  |  |  | p = 0.490 |  |
| weekPORTIONScosm |  |  |  |  |  |  |  | 0.008 (-0.007, 0.024) |
|  |  |  |  |  |  |  |  | p = 0.292 |
| Constant | 7.430 (6.554, 8.307) | 7.737 (6.864, 8.610) | 7.687 (6.723, 8.650) | 7.827 (6.925, 8.730) | 6.867 (5.795, 7.939) | 7.567 (6.608, 8.526) | 7.538 (6.561, 8.515) | 7.373 (6.292, 8.454) |
|  | p = 0.000 | p = 0.000 | p = 0.000 | p = 0.000 | p = 0.000 | p = 0.000 | p = 0.000 | p = 0.000 |
|  | | | | | | | | |
| Observations | 100 | 99 | 89 | 84 | 83 | 99 | 95 | 96 |
| R2 | 0.370 | 0.336 | 0.334 | 0.357 | 0.366 | 0.336 | 0.335 | 0.350 |
| Adjusted R2 | 0.336 | 0.300 | 0.294 | 0.315 | 0.325 | 0.301 | 0.297 | 0.314 |
| Residual Std. Error | 0.777 (df = 94) | 0.799 (df = 93) | 0.806 (df = 83) | 0.771 (df = 78) | 0.748 (df = 77) | 0.796 (df = 93) | 0.805 (df = 89) | 0.800 (df = 90) |
| F Statistic | 11.023\*\*\* (df = 5; 94) | 9.411\*\*\* (df = 5; 93) | 8.315\*\*\* (df = 5; 83) | 8.647\*\*\* (df = 5; 78) | 8.904\*\*\* (df = 5; 77) | 9.430\*\*\* (df = 5; 93) | 8.951\*\*\* (df = 5; 89) | 9.701\*\*\* (df = 5; 90) |
|  | | | | | | | | |
| *Note:* | p<0.1; p<0.05; p<0.01 | | | | | | | |

**Determinants of BPA exposure - linear regression analysis - Table S10 (additional)**

|  | | | | | | | | |
|  | Pooled population (CONTROLS only) - questionnaire exposures (adj. for study site, disease status, age, BMI | | | | | | | |
|  |  | | | | | | | |
|  | Spot BPA (ng/L, log transformed) | | | | | | | |
|  | (1) | (2) | (3) | (4) | (5) | (6) | (7) | (8) |
|  | | | | | | | | |
| ln\_creat\_gPERlt\_spot | 0.472 (0.234, 0.711) | 0.468 (0.226, 0.710) | 0.400 (0.147, 0.654) | 0.366 (0.107, 0.625) | 0.392 (0.101, 0.683) | 0.372 (0.128, 0.616) | 0.391 (0.133, 0.648) | 0.473 (0.223, 0.724) |
|  | p = 0.0002 | p = 0.0003 | p = 0.003 | p = 0.007 | p = 0.011 | p = 0.004 | p = 0.004 | p = 0.0004 |
| countryRO | 1.639 (1.210, 2.067) | 1.609 (1.173, 2.044) | 1.607 (1.170, 2.043) | 1.795 (1.356, 2.234) | 1.688 (1.156, 2.221) | 1.665 (1.258, 2.073) | 1.645 (1.217, 2.073) | 1.645 (1.221, 2.070) |
|  | p = 0.000 | p = 0.000 | p = 0.000 | p = 0.000 | p = 0.00000 | p = 0.000 | p = 0.000 | p = 0.000 |
| age | -0.003 (-0.021, 0.015) | -0.001 (-0.019, 0.017) | -0.003 (-0.021, 0.016) | -0.005 (-0.024, 0.013) | -0.005 (-0.026, 0.016) | -0.011 (-0.030, 0.008) | -0.002 (-0.021, 0.018) | -0.002 (-0.022, 0.018) |
|  | p = 0.753 | p = 0.916 | p = 0.755 | p = 0.570 | p = 0.613 | p = 0.253 | p = 0.851 | p = 0.837 |
| BMI | -0.004 (-0.055, 0.047) | 0.006 (-0.043, 0.054) | -0.005 (-0.057, 0.047) | -0.008 (-0.058, 0.042) | 0.003 (-0.051, 0.057) | -0.0001 (-0.048, 0.048) | -0.004 (-0.055, 0.047) | 0.006 (-0.044, 0.056) |
|  | p = 0.889 | p = 0.820 | p = 0.855 | p = 0.750 | p = 0.921 | p = 0.997 | p = 0.888 | p = 0.816 |
| daily20lglass | 0.071 (-0.023, 0.164) |  |  |  |  |  |  |  |
|  | p = 0.142 |  |  |  |  |  |  |  |
| MwaveFrw |  | -0.006 (-0.106, 0.094) |  |  |  |  |  |  |
|  |  | p = 0.903 |  |  |  |  |  |  |
| weekPORTIONScanned |  |  | 0.0005 (-0.065, 0.066) |  |  |  |  |  |
|  |  |  | p = 0.989 |  |  |  |  |  |
| weekMINScleaning |  |  |  | -0.00002 (-0.001, 0.001) |  |  |  |  |
|  |  |  |  | p = 0.965 |  |  |  |  |
| weekPORTIONSpcps |  |  |  |  | -0.003 (-0.011, 0.005) |  |  |  |
|  |  |  |  |  | p = 0.529 |  |  |  |
| perfwc |  |  |  |  |  | -0.076 (-0.139, -0.013) |  |  |
|  |  |  |  |  |  | p = 0.022 |  |  |
| weekPORTIONSdeo |  |  |  |  |  |  | -0.008 (-0.080, 0.065) |  |
|  |  |  |  |  |  |  | p = 0.838 |  |
| weekPORTIONScosm |  |  |  |  |  |  |  | -0.001 (-0.016, 0.015) |
|  |  |  |  |  |  |  |  | p = 0.920 |
| Constant | 7.644 (6.487, 8.801) | 7.428 (6.244, 8.611) | 7.760 (6.499, 9.022) | 7.931 (6.728, 9.134) | 7.904 (6.349, 9.460) | 8.450 (7.115, 9.786) | 7.743 (6.402, 9.085) | 7.469 (6.105, 8.833) |
|  | p = 0.000 | p = 0.000 | p = 0.000 | p = 0.000 | p = 0.000 | p = 0.000 | p = 0.000 | p = 0.000 |
|  | | | | | | | | |
| Observations | 98 | 98 | 94 | 91 | 76 | 97 | 94 | 95 |
| R2 | 0.485 | 0.480 | 0.464 | 0.522 | 0.504 | 0.514 | 0.476 | 0.478 |
| Adjusted R2 | 0.457 | 0.452 | 0.434 | 0.494 | 0.469 | 0.487 | 0.447 | 0.449 |
| Residual Std. Error | 0.984 (df = 92) | 0.986 (df = 92) | 0.990 (df = 88) | 0.963 (df = 85) | 0.994 (df = 70) | 0.951 (df = 91) | 0.982 (df = 88) | 0.997 (df = 89) |
| F Statistic | 17.343\*\*\* (df = 5; 92) | 16.994\*\*\* (df = 5; 92) | 15.251\*\*\* (df = 5; 88) | 18.570\*\*\* (df = 5; 85) | 14.224\*\*\* (df = 5; 70) | 19.254\*\*\* (df = 5; 91) | 16.018\*\*\* (df = 5; 88) | 16.332\*\*\* (df = 5; 89) |
|  | | | | | | | | |
| *Note:* | p<0.1; p<0.05; p<0.01 | | | | | | | |

# 6 BPA and TSH - trends

- Trend model A: univariate (only creatinine adjustment)
- Trend model B: adjustment for creatinine, age and BMI
- Trend model C: adjustment for creatinine, country, age and BMI
- Trend model D: adjustment for creatinine, country, age, BMI and disease status

## 6.1 Table 4

outcome: ln\_TSH dependent variables: quartiles of ln\_BPF, ln\_BPA, ln\_ClxBPA (continuous)

ONLY WORKING ON THE SPOT SAMPLES

For each models the results of the complete dataset are presented first followed by the results of the sensitivity analysis BPA, BPF and ClxBPA where only participants with values between the 5th and the 9th percentile have been included.

1. Univariate trend models and (2) adjustment: age + BMI - quantiles CONT

- Pooled sample

**TSH and BPF, BPA or ClxBPA - trend analysis - Pooled population - Table 4**

|  | | | |
|  | A - Creatinine (g/L, log-transformed) | | |
|  |  | | |
|  | TSH (mIU/L, log-transformed) | | |
|  | (1) | (2) | (3) |
|  | | | |
| ln\_creat\_gPERlt\_spot | -0.149 (-0.266, -0.032) | -0.057 (-0.171, 0.057) | -0.078 (-0.186, 0.030) |
|  | p = 0.014 | p = 0.329 | p = 0.161 |
| SUBquant1234\_ln\_BPA\_ngPERlt\_spot | 0.145 (0.045, 0.246) |  |  |
|  | p = 0.006 |  |  |
| SUBquant1234\_ln\_BPF\_ngPERlt\_spot |  | -0.063 (-0.211, 0.085) |  |
|  |  | p = 0.404 |  |
| SUBquant1234\_ln\_ClxBPA\_ngPERlt\_spot |  |  | 0.095 (-0.443, 0.633) |
|  |  |  | p = 0.730 |
| Constant | -0.780 (-1.581, 0.021) | 0.768 (-0.167, 1.703) | -0.116 (-2.876, 2.643) |
|  | p = 0.058 | p = 0.109 | p = 0.935 |
|  | | | |
| Observations | 206 | 206 | 206 |
| R2 | 0.047 | 0.013 | 0.010 |
| Adjusted R2 | 0.037 | 0.003 | -0.00002 |
| Residual Std. Error (df = 203) | 0.617 | 0.628 | 0.629 |
| F Statistic (df = 2; 203) | 4.988\*\*\* | 1.292 | 0.998 |
|  | | | |
| *Note:* | p<0.1; p<0.05; p<0.01 | | |

**Sensitivity analysis results: TSH and BPF, BPA or ClxBPA - trend analysis - Pooled population - BPA: 5to95 - Table 4**

|  | |
|  | A - Creatinine (g/L, log-transformed) |
|  |  |
|  | TSH (mIU/L, log-transformed) |
|  | |
| ln\_creat\_gPERlt\_spot | -0.145 (-0.270, -0.020) |
|  | p = 0.025 |
| SUBquant1234\_ln\_BPA\_ngPERlt\_spot | 0.125 (0.009, 0.240) |
|  | p = 0.036 |
| Constant | -0.642 (-1.557, 0.272) |
|  | p = 0.171 |
|  | |
| Observations | 184 |
| R2 | 0.036 |
| Adjusted R2 | 0.025 |
| Residual Std. Error | 0.631 (df = 181) |
| F Statistic | 3.337\*\* (df = 2; 181) |
|  | |
| *Note:* | p<0.1; p<0.05; p<0.01 |

**Sensitivity analysis results: TSH and BPF, BPF or ClxBPA - trend analysis - Pooled population - BPF: 5to95 - Table 4**

|  | |
|  | A - Creatinine (g/L, log-transformed) |
|  |  |
|  | TSH (mIU/L, log-transformed) |
|  | |
| ln\_creat\_gPERlt\_spot | -0.077 (-0.198, 0.043) |
|  | p = 0.209 |
| SUBquant1234\_ln\_BPF\_ngPERlt\_spot | -0.079 (-0.240, 0.082) |
|  | p = 0.337 |
| Constant | 0.831 (-0.180, 1.843) |
|  | p = 0.110 |
|  | |
| Observations | 184 |
| R2 | 0.020 |
| Adjusted R2 | 0.009 |
| Residual Std. Error | 0.615 (df = 181) |
| F Statistic | 1.874 (df = 2; 181) |
|  | |
| *Note:* | p<0.1; p<0.05; p<0.01 |

**Sensitivity analysis results: TSH and ClxBPA, BPF or ClxBPA - trend analysis - Pooled population - ClxBPA- Table 4**

|  | |
|  | A - Creatinine (g/L, log-transformed) |
|  |  |
|  | TSH (mIU/L, log-transformed) |
|  | |
| ln\_creat\_gPERlt\_spot | -0.085 (-0.196, 0.026) |
|  | p = 0.137 |
| SUBquant1234\_ln\_ClxBPA\_ngPERlt\_spot | 0.074 (-0.520, 0.667) |
|  | p = 0.809 |
| Constant | 0.017 (-3.024, 3.058) |
|  | p = 0.992 |
|  | |
| Observations | 184 |
| R2 | 0.012 |
| Adjusted R2 | 0.001 |
| Residual Std. Error | 0.624 (df = 181) |
| F Statistic | 1.120 (df = 2; 181) |
|  | |
| *Note:* | p<0.1; p<0.05; p<0.01 |

**TSH and BPF, BPA or ClxBPA - trend analysis - Pooled population - Table 4**

|  | | | |
|  | B - Age, BMI and creatinine (g/L, log-transformed) | | |
|  |  | | |
|  | TSH (mIU/L, log-transformed) | | |
|  | (1) | (2) | (3) |
|  | | | |
| ln\_creat\_gPERlt\_spot | -0.118 (-0.238, 0.003) | -0.041 (-0.159, 0.077) | -0.054 (-0.167, 0.059) |
|  | p = 0.057 | p = 0.499 | p = 0.354 |
| age | 0.003 (-0.004, 0.011) | 0.002 (-0.006, 0.009) | 0.002 (-0.006, 0.010) |
|  | p = 0.399 | p = 0.697 | p = 0.665 |
| BMI | -0.003 (-0.020, 0.015) | -0.002 (-0.019, 0.016) | -0.001 (-0.019, 0.016) |
|  | p = 0.758 | p = 0.860 | p = 0.875 |
| SUBquant1234\_ln\_BPA\_ngPERlt\_spot | 0.144 (0.041, 0.248) |  |  |
|  | p = 0.007 |  |  |
| SUBquant1234\_ln\_BPF\_ngPERlt\_spot |  | -0.033 (-0.186, 0.119) |  |
|  |  | p = 0.669 |  |
| SUBquant1234\_ln\_ClxBPA\_ngPERlt\_spot |  |  | 0.151 (-0.400, 0.703) |
|  |  |  | p = 0.592 |
| Constant | -0.851 (-1.819, 0.116) | 0.556 (-0.551, 1.662) | -0.442 (-3.314, 2.430) |
|  | p = 0.087 | p = 0.327 | p = 0.764 |
|  | | | |
| Observations | 200 | 200 | 200 |
| R2 | 0.042 | 0.007 | 0.007 |
| Adjusted R2 | 0.023 | -0.014 | -0.013 |
| Residual Std. Error (df = 195) | 0.618 | 0.629 | 0.629 |
| F Statistic (df = 4; 195) | 2.158\* | 0.331 | 0.358 |
|  | | | |
| *Note:* | p<0.1; p<0.05; p<0.01 | | |

**Sensitivity analysis results: TSH and BPF, BPA or ClxBPA - trend analysis - Pooled population - BPA: 5to95- Table 4**

|  | |
|  | B - Age, BMI and creatinine (g/L, log-transformed) |
|  |  |
|  | TSH (mIU/L, log-transformed) |
|  | |
| ln\_creat\_gPERlt\_spot | -0.123 (-0.252, 0.007) |
|  | p = 0.066 |
| age | 0.002 (-0.007, 0.010) |
|  | p = 0.713 |
| BMI | -0.004 (-0.022, 0.015) |
|  | p = 0.690 |
| SUBquant1234\_ln\_BPA\_ngPERlt\_spot | 0.124 (0.007, 0.242) |
|  | p = 0.040 |
| Constant | -0.607 (-1.673, 0.459) |
|  | p = 0.266 |
|  | |
| Observations | 179 |
| R2 | 0.032 |
| Adjusted R2 | 0.010 |
| Residual Std. Error | 0.634 (df = 174) |
| F Statistic | 1.430 (df = 4; 174) |
|  | |
| *Note:* | p<0.1; p<0.05; p<0.01 |

**Sensitivity analysis results: TSH and BPF, BPF or ClxBPA - trend analysis - Pooled population - BPF: 5to95 - Table 4**

|  | |
|  | B - Age, BMI and creatinine (g/L, log-transformed) |
|  |  |
|  | TSH (mIU/L, log-transformed) |
|  | |
| ln\_creat\_gPERlt\_spot | -0.051 (-0.177, 0.074) |
|  | p = 0.426 |
| age | 0.003 (-0.005, 0.012) |
|  | p = 0.416 |
| BMI | 0.002 (-0.017, 0.021) |
|  | p = 0.834 |
| SUBquant1234\_ln\_BPF\_ngPERlt\_spot | -0.045 (-0.211, 0.121) |
|  | p = 0.594 |
| Constant | 0.412 (-0.792, 1.616) |
|  | p = 0.504 |
|  | |
| Observations | 179 |
| R2 | 0.018 |
| Adjusted R2 | -0.005 |
| Residual Std. Error | 0.616 (df = 174) |
| F Statistic | 0.788 (df = 4; 174) |
|  | |
| *Note:* | p<0.1; p<0.05; p<0.01 |

**Sensitivity analysis results: TSH and BPF, BPF or ClxBPA - trend analysis - Pooled population - ClxBPA: 5to95 - Table 4**

|  | |
|  | B - Age, BMI and creatinine (g/L, log-transformed) |
|  |  |
|  | TSH (mIU/L, log-transformed) |
|  | |
| ln\_creat\_gPERlt\_spot | -0.063 (-0.181, 0.054) |
|  | p = 0.292 |
| age | 0.003 (-0.005, 0.012) |
|  | p = 0.417 |
| BMI | -0.003 (-0.022, 0.015) |
|  | p = 0.719 |
| SUBquant1234\_ln\_ClxBPA\_ngPERlt\_spot | 0.036 (-0.570, 0.641) |
|  | p = 0.909 |
| Constant | 0.134 (-3.027, 3.295) |
|  | p = 0.934 |
|  | |
| Observations | 181 |
| R2 | 0.014 |
| Adjusted R2 | -0.009 |
| Residual Std. Error | 0.628 (df = 176) |
| F Statistic | 0.606 (df = 4; 176) |
|  | |
| *Note:* | p<0.1; p<0.05; p<0.01 |

3. Adjustment: country + age + BMI and (4) adjustment: country + age + BMI and + status (only for the pooled sample) - quantiles CONT

- Pooled sample

**TSH and BPF, BPA or ClxBPA - trend analysis - Pooled population - Table 4**

|  | | | |
|  | 3. Age, BMI, study siteand creatinine | | |
|  |  | | |
|  | TSH (mIU/L, log-transformed) | | |
|  | (1) | (2) | (3) |
|  | | | |
| ln\_creat\_gPERlt\_spot | -0.118 (-0.240, 0.003) | -0.048 (-0.167, 0.071) | -0.055 (-0.168, 0.058) |
|  | p = 0.058 | p = 0.435 | p = 0.344 |
| countryRO | -0.008 (-0.206, 0.190) | 0.094 (-0.091, 0.278) | 0.091 (-0.107, 0.290) |
|  | p = 0.939 | p = 0.322 | p = 0.370 |
| age | 0.003 (-0.005, 0.011) | 0.002 (-0.006, 0.010) | 0.002 (-0.006, 0.010) |
|  | p = 0.413 | p = 0.555 | p = 0.550 |
| BMI | -0.003 (-0.020, 0.015) | -0.003 (-0.021, 0.015) | -0.003 (-0.021, 0.015) |
|  | p = 0.775 | p = 0.713 | p = 0.735 |
| SUBquant1234\_ln\_BPA\_ngPERlt\_spot | 0.146 (0.032, 0.260) |  |  |
|  | p = 0.013 |  |  |
| SUBquant1234\_ln\_BPF\_ngPERlt\_spot |  | -0.024 (-0.177, 0.129) |  |
|  |  | p = 0.759 |  |
| SUBquant1234\_ln\_ClxBPA\_ngPERlt\_spot |  |  | 0.047 (-0.550, 0.644) |
|  |  |  | p = 0.879 |
| Constant | -0.864 (-1.885, 0.157) | 0.462 (-0.660, 1.584) | 0.064 (-3.014, 3.141) |
|  | p = 0.099 | p = 0.421 | p = 0.968 |
|  | | | |
| Observations | 200 | 200 | 200 |
| R2 | 0.042 | 0.012 | 0.011 |
| Adjusted R2 | 0.018 | -0.014 | -0.014 |
| Residual Std. Error (df = 194) | 0.619 | 0.629 | 0.629 |
| F Statistic (df = 5; 194) | 1.719 | 0.462 | 0.448 |
|  | | | |
| *Note:* | p<0.1; p<0.05; p<0.01 | | |

**Sensitivity analysis results: TSH and BPF, BPA or ClxBPA - trend analysis - Pooled population - BPA: 5to95 - Table 4**

|  | |
|  | 3. Age, BMI, study site and creatinine |
|  |  |
|  | TSH (mIU/L, log-transformed) |
|  | |
| ln\_creat\_gPERlt\_spot | -0.124 (-0.255, 0.006) |
|  | p = 0.064 |
| countryRO | -0.026 (-0.234, 0.182) |
|  | p = 0.809 |
| age | 0.001 (-0.007, 0.010) |
|  | p = 0.746 |
| BMI | -0.003 (-0.022, 0.016) |
|  | p = 0.737 |
| SUBquant1234\_ln\_BPA\_ngPERlt\_spot | 0.130 (0.004, 0.255) |
|  | p = 0.045 |
| Constant | -0.643 (-1.751, 0.465) |
|  | p = 0.257 |
|  | |
| Observations | 179 |
| R2 | 0.032 |
| Adjusted R2 | 0.004 |
| Residual Std. Error | 0.636 (df = 173) |
| F Statistic | 1.150 (df = 5; 173) |
|  | |
| *Note:* | p<0.1; p<0.05; p<0.01 |

**Sensitivity analysis results: TSH and BPF, BPA or ClxBPA - trend analysis - Pooled population - BPF- Table 4**

|  | |
|  | 3. Age, BMI, study site and creatinine |
|  |  |
|  | TSH (mIU/L, log-transformed) |
|  | |
| ln\_creat\_gPERlt\_spot | -0.054 (-0.183, 0.075) |
|  | p = 0.414 |
| countryRO | 0.020 (-0.180, 0.220) |
|  | p = 0.848 |
| age | 0.004 (-0.005, 0.012) |
|  | p = 0.405 |
| BMI | 0.002 (-0.018, 0.022) |
|  | p = 0.869 |
| SUBquant1234\_ln\_BPF\_ngPERlt\_spot | -0.043 (-0.211, 0.125) |
|  | p = 0.618 |
| Constant | 0.392 (-0.833, 1.616) |
|  | p = 0.532 |
|  | |
| Observations | 179 |
| R2 | 0.018 |
| Adjusted R2 | -0.010 |
| Residual Std. Error | 0.617 (df = 173) |
| F Statistic | 0.634 (df = 5; 173) |
|  | |
| *Note:* | p<0.1; p<0.05; p<0.01 |

**Sensitivity analysis results: TSH and BPF, BPA or ClxBPA - trend analysis - Pooled population - ClxBPA- Table 4**

|  | |
|  | 3. Age, BMI, study site and creatinine |
|  |  |
|  | TSH (mIU/L, log-transformed) |
|  | |
| ln\_creat\_gPERlt\_spot | -0.064 (-0.182, 0.053) |
|  | p = 0.285 |
| countryRO | 0.056 (-0.153, 0.264) |
|  | p = 0.602 |
| age | 0.004 (-0.005, 0.012) |
|  | p = 0.373 |
| BMI | -0.004 (-0.023, 0.014) |
|  | p = 0.652 |
| SUBquant1234\_ln\_ClxBPA\_ngPERlt\_spot | -0.034 (-0.695, 0.627) |
|  | p = 0.919 |
| Constant | 0.471 (-2.940, 3.882) |
|  | p = 0.787 |
|  | |
| Observations | 181 |
| R2 | 0.015 |
| Adjusted R2 | -0.013 |
| Residual Std. Error | 0.629 (df = 175) |
| F Statistic | 0.538 (df = 5; 175) |
|  | |
| *Note:* | p<0.1; p<0.05; p<0.01 |

**TSH and BPF, BPA or ClxBPA - trend analysis - Pooled population - Table 4**

|  | | | |
|  | 4. Age, BMI, study sitedisease status[case] (and creatinine) | | |
|  |  | | |
|  | TSH (mIU/L, log-transformed) | | |
|  | (1) | (2) | (3) |
|  | | | |
| ln\_creat\_gPERlt\_spot | -0.101 (-0.221, 0.019) | -0.043 (-0.159, 0.073) | -0.047 (-0.157, 0.063) |
|  | p = 0.101 | p = 0.470 | p = 0.406 |
| countryRO | 0.026 (-0.169, 0.222) | 0.113 (-0.068, 0.293) | 0.112 (-0.082, 0.306) |
|  | p = 0.794 | p = 0.224 | p = 0.259 |
| age | 0.005 (-0.003, 0.012) | 0.004 (-0.004, 0.012) | 0.004 (-0.004, 0.012) |
|  | p = 0.250 | p = 0.320 | p = 0.317 |
| BMI | 0.001 (-0.016, 0.019) | 0.001 (-0.017, 0.019) | 0.001 (-0.017, 0.019) |
|  | p = 0.900 | p = 0.922 | p = 0.908 |
| status011 | -0.268 (-0.443, -0.093) | -0.295 (-0.470, -0.119) | -0.295 (-0.471, -0.119) |
|  | p = 0.004 | p = 0.002 | p = 0.002 |
| SUBquant1234\_ln\_BPA\_ngPERlt\_spot | 0.121 (0.008, 0.234) |  |  |
|  | p = 0.037 |  |  |
| SUBquant1234\_ln\_BPF\_ngPERlt\_spot |  | -0.014 (-0.164, 0.135) |  |
|  |  | p = 0.853 |  |
| SUBquant1234\_ln\_ClxBPA\_ngPERlt\_spot |  |  | 0.019 (-0.564, 0.602) |
|  |  |  | p = 0.950 |
| Constant | -0.704 (-1.710, 0.302) | 0.353 (-0.744, 1.449) | 0.161 (-2.842, 3.164) |
|  | p = 0.172 | p = 0.530 | p = 0.917 |
|  | | | |
| Observations | 200 | 200 | 200 |
| R2 | 0.085 | 0.064 | 0.064 |
| Adjusted R2 | 0.056 | 0.035 | 0.035 |
| Residual Std. Error (df = 193) | 0.607 | 0.614 | 0.614 |
| F Statistic (df = 6; 193) | 2.985\*\*\* | 2.205\*\* | 2.200\*\* |
|  | | | |
| *Note:* | p<0.1; p<0.05; p<0.01 | | |

**Sensitivity analysis results: TSH and BPF, BPA or ClxBPA - trend analysis - Pooled population - BPA- Table 4**

|  | |
|  | 4. Age, BMI, study site, disease status[case] (and creatinine) |
|  |  |
|  | TSH (mIU/L, log-transformed) |
|  | |
| ln\_creat\_gPERlt\_spot | -0.112 (-0.241, 0.016) |
|  | p = 0.090 |
| countryRO | 0.018 (-0.189, 0.226) |
|  | p = 0.864 |
| age | 0.003 (-0.006, 0.011) |
|  | p = 0.500 |
| BMI | -0.001 (-0.019, 0.018) |
|  | p = 0.957 |
| status011 | -0.254 (-0.445, -0.063) |
|  | p = 0.011 |
| SUBquant1234\_ln\_BPA\_ngPERlt\_spot | 0.113 (-0.011, 0.237) |
|  | p = 0.076 |
| Constant | -0.543 (-1.635, 0.550) |
|  | p = 0.332 |
|  | |
| Observations | 179 |
| R2 | 0.069 |
| Adjusted R2 | 0.036 |
| Residual Std. Error | 0.625 (df = 172) |
| F Statistic | 2.117\* (df = 6; 172) |
|  | |
| *Note:* | p<0.1; p<0.05; p<0.01 |

**Sensitivity analysis results: TSH and BPF, BPA or ClxBPA - trend analysis - Pooled population - BPF- Table 4**

|  | |
|  | 4. Age, BMI, study site, disease status[case] (and creatinine) |
|  |  |
|  | TSH (mIU/L, log-transformed) |
|  | |
| ln\_creat\_gPERlt\_spot | -0.044 (-0.171, 0.082) |
|  | p = 0.493 |
| countryRO | 0.044 (-0.153, 0.241) |
|  | p = 0.664 |
| age | 0.005 (-0.003, 0.014) |
|  | p = 0.215 |
| BMI | 0.003 (-0.016, 0.023) |
|  | p = 0.735 |
| status011 | -0.259 (-0.440, -0.077) |
|  | p = 0.006 |
| SUBquant1234\_ln\_BPF\_ngPERlt\_spot | -0.031 (-0.195, 0.134) |
|  | p = 0.718 |
| Constant | 0.312 (-0.891, 1.514) |
|  | p = 0.613 |
|  | |
| Observations | 179 |
| R2 | 0.061 |
| Adjusted R2 | 0.028 |
| Residual Std. Error | 0.606 (df = 172) |
| F Statistic | 1.847\* (df = 6; 172) |
|  | |
| *Note:* | p<0.1; p<0.05; p<0.01 |

**Sensitivity analysis results: TSH and BPF, BPA or ClxBPA - trend analysis - Pooled population - ClxBPA- Table 4**

|  | |
|  | 4. Age, BMI, study site, disease status[case] (and creatinine) |
|  |  |
|  | TSH (mIU/L, log-transformed) |
|  | |
| ln\_creat\_gPERlt\_spot | -0.054 (-0.170, 0.062) |
|  | p = 0.365 |
| countryRO | 0.079 (-0.127, 0.285) |
|  | p = 0.454 |
| age | 0.005 (-0.003, 0.013) |
|  | p = 0.250 |
| BMI | -0.0003 (-0.019, 0.018) |
|  | p = 0.979 |
| status011 | -0.255 (-0.441, -0.068) |
|  | p = 0.009 |
| SUBquant1234\_ln\_ClxBPA\_ngPERlt\_spot | -0.091 (-0.741, 0.560) |
|  | p = 0.786 |
| Constant | 0.719 (-2.638, 4.076) |
|  | p = 0.676 |
|  | |
| Observations | 181 |
| R2 | 0.054 |
| Adjusted R2 | 0.021 |
| Residual Std. Error | 0.618 (df = 174) |
| F Statistic | 1.659 (df = 6; 174) |
|  | |
| *Note:* | p<0.1; p<0.05; p<0.01 |

## 6.2 Additional trend analysis for fT4

### 6.2.1 Table S9

Linear regression outcome: ln\_ft4 dependent variables: quartiles of ln\_BPF, ln\_BPA, ln\_ClxBPA (continuous)

```
#CORRECTION 160822 - REMOVED THE ARGUMENT FOR "EXTERNAL" OUTPUT ADDED THE ", report = "vcsp""
stargazer(c(univ_trendCONT_ft4, adjustment1CONT_ft4), type="html", ci=TRUE, single.row = TRUE, title="fT4 and BPF, BPA or ClxBPA - trend analysis - Pooled population - Table S9", report = "vcsp")
```

**fT4 and BPF, BPA or ClxBPA - trend analysis - Pooled population - Table S9**

|  | | | | | | |
|  | *Dependent variable:* | | | | | |
|  |  | | | | | |
|  | ln\_ft4 | | | | | |
|  | (1) | (2) | (3) | (4) | (5) | (6) |
|  | | | | | | |
| ln\_creat\_gPERlt\_spot | 0.001 (-0.026, 0.029) | 0.016 (-0.011, 0.043) | 0.011 (-0.014, 0.036) | 0.004 (-0.024, 0.033) | 0.018 (-0.010, 0.045) | 0.015 (-0.012, 0.041) |
|  | p = 0.930 | p = 0.241 | p = 0.392 | p = 0.756 | p = 0.202 | p = 0.278 |
| age |  |  |  | 0.00002 (-0.002, 0.002) | -0.0003 (-0.002, 0.002) | -0.0002 (-0.002, 0.002) |
|  |  |  |  | p = 0.980 | p = 0.774 | p = 0.823 |
| BMI |  |  |  | 0.003 (-0.001, 0.007) | 0.004 (-0.001, 0.008) | 0.004 (-0.001, 0.008) |
|  |  |  |  | p = 0.105 | p = 0.096 | p = 0.093 |
| SUBquant1234\_ln\_BPA\_ngPERlt\_spot | 0.023 (-0.001, 0.047) |  |  | 0.025 (0.001, 0.049) |  |  |
|  | p = 0.061 |  |  | p = 0.046 |  |  |
| SUBquant1234\_ln\_BPF\_ngPERlt\_spot |  | -0.011 (-0.046, 0.024) |  |  | -0.006 (-0.042, 0.029) |  |
|  |  | p = 0.530 |  |  | p = 0.723 |  |
| SUBquant1234\_ln\_ClxBPA\_ngPERlt\_spot |  |  | 0.058 (-0.068, 0.184) |  |  | 0.063 (-0.065, 0.191) |
|  |  |  | p = 0.368 |  |  | p = 0.338 |
| Constant | 2.425 (2.235, 2.615) | 2.678 (2.458, 2.897) | 2.309 (1.661, 2.957) | 2.326 (2.099, 2.553) | 2.574 (2.316, 2.833) | 2.208 (1.539, 2.876) |
|  | p = 0.000 | p = 0.000 | p = 0.000 | p = 0.000 | p = 0.000 | p = 0.000 |
|  | | | | | | |
| Observations | 204 | 204 | 204 | 198 | 198 | 198 |
| R2 | 0.023 | 0.007 | 0.009 | 0.043 | 0.024 | 0.028 |
| Adjusted R2 | 0.013 | -0.003 | -0.001 | 0.024 | 0.004 | 0.008 |
| Residual Std. Error | 0.146 (df = 201) | 0.147 (df = 201) | 0.147 (df = 201) | 0.145 (df = 193) | 0.146 (df = 193) | 0.146 (df = 193) |
| F Statistic | 2.315 (df = 2; 201) | 0.717 (df = 2; 201) | 0.928 (df = 2; 201) | 2.186\* (df = 4; 193) | 1.184 (df = 4; 193) | 1.389 (df = 4; 193) |
|  | | | | | | |
| *Note:* | p<0.1; p<0.05; p<0.01 | | | | | |

```
stargazer(c(adjustment2CONT_ft4, adjustment3CONT_ft4), type="html", ci=TRUE, title="fT4 and BPF, BPA or ClxBPA - trend analysis - Pooled population - Table S9", single.row = TRUE, report = "vcsp")
```

**fT4 and BPF, BPA or ClxBPA - trend analysis - Pooled population - Table S9**

|  | | | | | | |
|  | *Dependent variable:* | | | | | |
|  |  | | | | | |
|  | ln\_ft4 | | | | | |
|  | (1) | (2) | (3) | (4) | (5) | (6) |
|  | | | | | | |
| ln\_creat\_gPERlt\_spot | 0.013 (-0.014, 0.039) | 0.009 (-0.016, 0.035) | 0.013 (-0.011, 0.037) | 0.013 (-0.013, 0.040) | 0.009 (-0.016, 0.035) | 0.013 (-0.011, 0.037) |
|  | p = 0.345 | p = 0.471 | p = 0.297 | p = 0.333 | p = 0.469 | p = 0.293 |
| countryRO | 0.123 (0.080, 0.166) | 0.121 (0.081, 0.160) | 0.131 (0.089, 0.173) | 0.124 (0.080, 0.167) | 0.121 (0.082, 0.161) | 0.131 (0.089, 0.174) |
|  | p = 0.00000 | p = 0.000 | p = 0.000 | p = 0.00000 | p = 0.000 | p = 0.000 |
| age | 0.001 (-0.001, 0.002) | 0.001 (-0.001, 0.003) | 0.001 (-0.001, 0.003) | 0.001 (-0.001, 0.003) | 0.001 (-0.001, 0.003) | 0.001 (-0.001, 0.003) |
|  | p = 0.385 | p = 0.365 | p = 0.360 | p = 0.371 | p = 0.353 | p = 0.347 |
| BMI | 0.001 (-0.003, 0.005) | 0.001 (-0.003, 0.005) | 0.001 (-0.003, 0.005) | 0.001 (-0.003, 0.005) | 0.001 (-0.003, 0.005) | 0.001 (-0.003, 0.005) |
|  | p = 0.536 | p = 0.511 | p = 0.565 | p = 0.511 | p = 0.489 | p = 0.541 |
| status011 |  |  |  | -0.006 (-0.046, 0.033) | -0.006 (-0.044, 0.033) | -0.006 (-0.044, 0.033) |
|  |  |  |  | p = 0.750 | p = 0.776 | p = 0.768 |
| SUBquant1234\_ln\_BPA\_ngPERlt\_spot | -0.004 (-0.029, 0.021) |  |  | -0.005 (-0.030, 0.020) |  |  |
|  | p = 0.737 |  |  | p = 0.707 |  |  |
| SUBquant1234\_ln\_BPF\_ngPERlt\_spot |  | 0.006 (-0.027, 0.039) |  |  | 0.006 (-0.027, 0.039) |  |
|  |  | p = 0.732 |  |  | p = 0.722 |  |
| SUBquant1234\_ln\_ClxBPA\_ngPERlt\_spot |  |  | -0.087 (-0.214, 0.041) |  |  | -0.087 (-0.215, 0.040) |
|  |  |  | p = 0.184 |  |  | p = 0.183 |
| Constant | 2.523 (2.301, 2.746) | 2.451 (2.210, 2.692) | 2.932 (2.275, 3.588) | 2.527 (2.303, 2.751) | 2.448 (2.206, 2.691) | 2.933 (2.274, 3.591) |
|  | p = 0.000 | p = 0.000 | p = 0.000 | p = 0.000 | p = 0.000 | p = 0.000 |
|  | | | | | | |
| Observations | 198 | 198 | 198 | 198 | 198 | 198 |
| R2 | 0.178 | 0.178 | 0.185 | 0.178 | 0.178 | 0.185 |
| Adjusted R2 | 0.156 | 0.156 | 0.164 | 0.152 | 0.152 | 0.160 |
| Residual Std. Error | 0.134 (df = 192) | 0.134 (df = 192) | 0.134 (df = 192) | 0.135 (df = 191) | 0.135 (df = 191) | 0.134 (df = 191) |
| F Statistic | 8.296\*\*\* (df = 5; 192) | 8.297\*\*\* (df = 5; 192) | 8.702\*\*\* (df = 5; 192) | 6.898\*\*\* (df = 6; 191) | 6.895\*\*\* (df = 6; 191) | 7.232\*\*\* (df = 6; 191) |
|  | | | | | | |
| *Note:* | p<0.1; p<0.05; p<0.01 | | | | | |

## 6.3 Plots between BPA, TSH and iodine

```
## Warning: Removed 14 rows containing non-finite values (stat_smooth).
```

```
## Warning: Removed 14 rows containing missing values (geom_point).
```

```
## Warning: Removed 15 rows containing non-finite values (stat_smooth).
```

```
## Warning: Removed 15 rows containing missing values (geom_point).
```

```
## Warning: Removed 3 rows containing non-finite values (stat_smooth).
```

```
## Warning: Removed 3 rows containing missing values (geom_point).
```

```
## Warning: Removed 3 rows containing non-finite values (stat_smooth).

## Warning: Removed 3 rows containing missing values (geom_point).
```

```
## Warning: Removed 11 rows containing non-finite values (stat_smooth).
```

```
## Warning: Removed 11 rows containing missing values (geom_point).
```

```
## Warning: Removed 12 rows containing non-finite values (stat_smooth).
```

```
## Warning: Removed 12 rows containing missing values (geom_point).
```

R and package versions used

```
sessionInfo()
```

```
## R version 3.3.1 (2016-06-21)
## Platform: i386-w64-mingw32/i386 (32-bit)
## Running under: Windows 7 (build 7601) Service Pack 1
## 
## locale:
## [1] LC_COLLATE=Greek_Greece.1253  LC_CTYPE=Greek_Greece.1253   
## [3] LC_MONETARY=Greek_Greece.1253 LC_NUMERIC=C                 
## [5] LC_TIME=Greek_Greece.1253    
## 
## attached base packages:
## [1] stats     graphics  grDevices utils     datasets  methods   base     
## 
## other attached packages:
##  [1] knitr_1.14       car_2.1-3        coefplot_1.2.4   irr_0.84         lpSolve_5.6.13  
##  [6] ICC_2.3.0        sjPlot_2.0.2     tableone_0.7.3   corrplot_0.77    plyr_1.8.4      
## [11] psych_1.6.6      stargazer_5.2    Hmisc_3.17-4     Formula_1.2-1    survival_2.39-4 
## [16] lattice_0.20-33  data.table_1.9.6 dplyr_0.5.0      ggplot2_2.1.0   
## 
## loaded via a namespace (and not attached):
##  [1] Rcpp_0.12.6         magrittr_1.5        cluster_2.0.4       MASS_7.3-45        
##  [5] splines_3.3.1       mnormt_1.5-4        colorspace_1.2-6    R6_2.1.3           
##  [9] parallel_3.3.1      broom_0.4.1         e1071_1.6-7         latticeExtra_0.6-28
## [13] TH.data_1.0-7       class_7.3-14        MatrixModels_0.4-1  sjstats_0.3.0      
## [17] sjmisc_1.8          htmltools_0.3.5     modeltools_0.2-21   lme4_1.1-12        
## [21] assertthat_0.1      digest_0.6.10       tibble_1.1          Matrix_1.2-6       
## [25] acepack_1.3-3.3     haven_0.2.1         coin_1.1-2          rmarkdown_1.0      
## [29] scales_0.4.0        stats4_3.3.1        survey_3.31         munsell_0.4.3      
## [33] multcomp_1.4-6      minqa_1.2.4         highr_0.6           stringr_1.1.0      
## [37] tools_3.3.1         pbkrtest_0.4-6      grid_3.3.1          nnet_7.3-12        
## [41] nlme_3.1-128        gtable_0.2.0        mgcv_1.8-12         quantreg_5.26      
## [45] DBI_0.5             lazyeval_0.2.0      yaml_2.1.13         useful_1.2.1       
## [49] gridExtra_2.2.1     formatR_1.4         tidyr_0.6.0         nloptr_1.0.4       
## [53] reshape2_1.4.1      RColorBrewer_1.1-2  effects_3.1-1       codetools_0.2-14   
## [57] stringdist_0.9.4.1  rpart_4.1-10        evaluate_0.9        labeling_0.3       
## [61] sandwich_2.3-4      stringi_1.1.1       SparseM_1.7         mvtnorm_1.0-5      
## [65] foreign_0.8-66      chron_2.3-47        zoo_1.7-13
```
